# Cell‐type‐specific modulation of innate immune signalling by vitamin D in human mononuclear phagocytes

**DOI:** 10.1111/imm.12669

**Published:** 2016-10-03

**Authors:** Rhiannon Kundu, Aikaterini Theodoraki, Carolin T. Haas, Yanjing Zhang, Benjamin Chain, Janos Kriston‐Vizi, Mahdad Noursadeghi, Bernard Khoo

**Affiliations:** ^1^ Division of Infection and ImmunityUniversity College LondonLondonUK; ^2^ EndocrinologyDivision of MedicineUniversity College LondonLondonUK; ^3^ MRC Laboratory for Molecular Cell BiologyUniversity College LondonLondonUK; ^4^ Present address: Department of ImmunobiologyKing's College LondonLondonUK

**Keywords:** dendritic cells, macrophages, mitogen‐activated protein kinase, monocytes, nuclear factor‐*κ*B, vitamin D

## Abstract

Vitamin D is widely reported to inhibit innate immune signalling and dendritic cell (DC) maturation as a potential immunoregulatory mechanism. It is not known whether vitamin D has global or gene‐specific effects on transcriptional responses downstream of innate immune stimulation, or whether vitamin D inhibition of innate immune signalling is common to different cells. We confirmed vitamin D inhibition of nuclear factor‐*κ*B (NF‐*κ*B) and p38 mitogen‐activated protein kinase (MAPK) signalling in monocyte‐derived DC (MDDC) stimulated with lipopolysaccharide (LPS). This was associated with global but modest attenuation of LPS‐induced transcriptional changes at genome‐wide level. Surprisingly, vitamin D did not inhibit innate immune NF‐*κ*B activation in monocyte‐derived macrophages. Consistent with our findings in MDDC,* ex vivo* vitamin D treatment of primary peripheral blood myeloid DC also led to significant inhibition of LPS‐inducible NF‐*κ*B activation. Unexpectedly, in the same samples, vitamin D enhanced activation of both NF‐*κ*B and MAPK signalling in primary peripheral blood monocytes. In a cross‐sectional clinical cohort, we found no relationship between peripheral blood vitamin D levels and LPS‐inducible activation of NF‐*κ*B and MAPK pathways in monocytes of myeloid DC. Remarkably, however, *in vivo* supplementation of people with vitamin D deficiency in this clinical cohort also enhanced LPS‐inducible MAPK signalling in peripheral blood monocytes. Therefore, we report that vitamin D differentially modulates the molecular response to innate immune stimulation in monocytes, macrophages and dendritic cells. These results are of importance in the design of studies on vitamin D supplementation in infectious and immunological diseases.

## Introduction

Vitamin D deficiency is epidemiologically associated with a wide range of autoimmune diseases. Consequently, extensive research interest has focused on the role of vitamin D in immune regulation and tolerance. Vitamin D is synthesized as a pro‐hormone from 7‐dehydrocholesterol in ultraviolet‐B‐irradiated skin followed by thermal isomerization, or is replenished by dietary intake, and 25‐hydroxylated constitutively in the liver to form 25‐hydroxyvitamin D (25[OH]D).^1^ Further 1*α*‐hydroxylation is catalysed by the action of CYP27B1 to generate the active moiety, 1,25[OH]D. Generation of 1,25[OH]D in the kidney is a key step for endocrine regulation of calcium homeostasis, but immune cells can also activate vitamin D as a result of innate immune up‐regulation of CYP27B1, particularly by macrophages^2^ with downstream autocrine and paracrine effects on diverse immune cells that express the vitamin D receptor. A substantial body of data show that 1,25[OH]D can regulate T‐cell responses either by inhibition of dendritic cell (DC) ‐dependent T‐cell activation or by induction of regulatory T cells.^3^, ^4^, ^5^, ^6^ Vitamin D inhibition of DC‐dependent T‐cell activation has been primarily attributed to the inhibition of innate immune up‐regulation of MHC class II and co‐stimulatory molecules such as interleukin‐12 (IL‐12) and CD80/86, which are critical for activation of naive T cells.^3^ The underlying mechanism for these effects of vitamin D on the canonical function of DC remain poorly understood, but the prevailing view is that 1,25[OH]D attenuates innate immune intracellular signalling pathways. *Ex vivo* treatment of peripheral blood myeloid DC with 1,25[OH]D is reported to inhibit lipopolysaccharide (LPS) ‐induced activation of the classical nuclear factor‐*κ*B (NF‐*κ*B) pathway,^7^ and *ex vivo* treatment of peripheral blood mononuclear cells (PBMC) with either 25[OH]D or 1,25[OH]D has been reported to inhibit LPS‐induced activation of the mitogen‐activated protein kinase (MAPK) pathway in monocytes.^8^ Whether these effects of vitamin D are also evident *in vivo* has not been tested in human studies. We aimed to extend the existing data on vitamin D modulation of innate immune cellular activation by addressing two questions. First, given the pivotal role of the NF‐*κ*B and MAPK pathways in initiating transcriptional responses to innate immune stimuli, we sought to test whether vitamin D inhibited all such responses or inhibited targeted genes selectively. Second, in view of the interest in dietary vitamin D supplementation as a countermeasure to autoimmune diseases associated with vitamin D deficiency, we sought to test the hypothesis in a clinical cohort that vitamin D deficiency and *in vivo* vitamin D supplementation modulate innate immune activation of peripheral blood monocytes and myeloid DC consistent with the *ex vivo* studies described above.

## Methods

### Innate immune stimulation of blood‐derived cells

The study was approved by the joint University College London/University College London Hospitals National Health Service Trust Human Research Ethics Committee (ref. 10/H0720/14). The PBMCs were obtained by venepuncture of healthy volunteers and density‐gradient centrifugation. Monocyte‐derived dendritic cells (MDDC) were derived from magnetically sorted CD14‐positive monocytes differentiated in RPMI‐1640 culture media (Sigma, St Louis, MO) with 10% fetal calf serum, 100 ng/ml granulocyte–macrophage colony‐stimulating factor (GM‐CSF) and 50 ng/ml IL‐4 for 4 days.^2^ Monocyte‐derived macrophages (MDM) were derived after adherence to tissue‐culture plastic and culture for 6 days in RPMI‐1640 with 10% autologous serum and 20 ng/ml macrophage colony‐stimulating factor (M‐CSF).^2^ The PBMC, MDDC and MDM were stimulated with either 100 ng/ml ultrapure LPS (Invivogen, San Diego, CA) as Toll‐like receptor 4 (TLR4) ligand, or with 1 μg/ml Pam_2_CSK_4_ (PCSK, Invivogen) as a TLR2 ligand for 30 min to measure phosphorylation of p38 and NF‐*κ*B p65 by flow cytometry, 60 min to undertake quantitative confocal microscopy to evaluate nuclear translocation of NF‐*κ*B p65, and 4 hr to measure transcriptional responses.

### Flow cytometry

Cells were stained with live/dead stain (Invitrogen, Carlsbad, CA) before fixation in Fix I Buffer (BD Biosciences, San Jose, CA) and immunostaining for cell surface CD14 (clone M5‐E2) and CD11c (clone B‐ly6) (BD Biosciences). For intracellular markers, cells were permeabilized with Perm/Wash buffer (BD Biosciences) before staining with antibodies to phospho‐NF‐*κ*B p65 (clone 20/NF‐*κ*B/p65) and phospho‐p38 MAPK (clone 36/p38) (BD Biosciences) as per the manufacturer's instructions, and analysed on an LSR‐II flow cytometer (BD Biosciences). The mean fluorescence intensity of staining of each phospho‐protein was used to calculate an activation index represented by the ratio of stimulated to unstimulated samples.

### Transcriptional analysis by quantitative PCR and genome‐wide expression arrays

RNA samples were collected in RLT buffer and extracted with the RNA Easy Kit (Qiagen, Hilden, Germany). All samples were DNase (Ambion, Foster City, CA) treated per the manufacturer's instructions. For quantitative PCR, first‐strand cDNA was synthesized using the qScript cDNA Supermix kit (QuantaBio, Beverley, MA). Real‐time quantitative RT‐PCR (qPCR) of tumour necrosis factor‐*α* (TNF‐*α*) (Hs00174128_m1), IL‐1*β* (Hs01555410_m1) and IL‐6 (Hs00985639_m1) was performed with Taqman primer probe sets (Applied Biosystems, Foster City, CA). Expression levels of target genes were normalized to that of glyceraldehyde 3‐phosphate dehydrogenase (GAPDH) using established primer probe sets.^9^ Genome‐wide transcriptional profiling was performed using Agilent microarrays as previously described.^10^, ^11^ Principal component analysis was used to compare global gene expression profiles as previously described^10^ and paired *t*‐tests with Welch approximation and > 2‐fold change filter were used to identify significant gene expression differences (*P* < 0·05) between groups using multiexperiment viewer (v4·9). Transcription factor binding site enrichment analysis was performed using the human single site analysis function in opossum.^12^ Upstream regulator analysis was performed with Qiagen's Ingenuity Pathway Analysis (www.ingenuity.com/), filtering for molecule types to include only genes, RNA and protein. Genes identified as target molecules of p38 MAPK in this upstream analysis were used as gene expression module for p38 MAPK activity, and their geometric mean expression was defined as p38 module score. All microarray data used in this study are available in arrayexpress (https://www.ebi.ac.uk/arrayexpress/) under the accession numbers E‐TABM‐1206 and E‐MTAB‐4883.

### Confocal microscopy

Nuclear : cytoplasmic ratios of NF‐*κ*B p65 were derived from image analysis (metamorph v7·17, Molecular Devices, Sunnyvale, CA) of confocal immunofluorescence stains obtained with a spinning disc PerkinElmer Opera LX microscope using rabbit polyclonal anti NF‐*κ*B RelA (C‐20) (Santa Cruz Biotechnology, Dallas, TX) and DAPI staining as previously described.^13^ Cells were seeded into 96‐well, optically clear‐bottomed, tissue‐culture‐treated, sterile Viewplate‐96 (PerkinElmer, Waltham, MA, product number 6005182) microplates. Olympus 40 × LUCPLFLN (Numerical aperture = 0·6) air lens was used to acquire images at two fluorescence channels using 365 nm and 561 nm excitation wavelengths, respectively. Camera binning 2 was used, which resulted in a pixel size of 0·323 μm. Flatfield correction and skew‐crop analysis was performed for optical correction. To compensate the systematic uneven brightness distribution of the optics, reference images were taken for flat‐field correction. The ‘fish‐eye effect’ and offset between channels were compensated using bead images for skew‐crop analysis. A standard Opera Adjustment Plate (PerkinElmer, product number HH10000650) was used for both flat field correction and skew‐crop analysis before image acquisition of each experiment, following the manufacturer's recommended procedures. Twenty‐five image fields were acquired, arranged in a 5 × 5 sub‐layout at the middle of each well.

### *In vivo vitamin D supplementation patient cohort*

Adult patients, aged 16 years or over, were recruited from the Endocrinology clinics at the Royal Free Hospital for suspected or confirmed vitamin D deficiency, secondary hyperparathyroidism, osteoporosis or osteopenia. The study was approved by the UCL/UCLH/Royal Free Biomedical Research Unit and an NHS Local Research Ethics Committee (Reference: 10/H0720/14). Key exclusion criteria were those taking any immunosuppressant or immunomodulator drugs (e.g. carbimazole, methimazole, glucocorticoids, antibiotics), those with currently active infection, recent immunization within 1 month and, pregnancy. Serum 25[OH]D levels were determined by Roche Elecsys total Vitamin D immunoassay (Roche Diagnostics, Burgess Hill, UK) which detects both 25[OH] vitamins D3 and D2, and which has intra‐assay CV of 1·7–7·8% and inter‐assay CV of < 2·2–13·1%. Where patients were shown to have a 25[OH]D > 75 nm, no supplementation was given. Where patients were shown to have a 25[OH]D ≤ 75 nm, they were treated with Colecalciferol (vitamin D3) for 3 months at a dose of 1000–2000 iu/day according to the treating physician's discretion before re‐assessment of 25[OH]D levels. Pre‐ and post‐supplementation blood samples were taken 3 months apart. PBMC were isolated from BD Vacutainer^®^ CPT^™^ Cell Preparation Tubes and cryopreserved for batch testing. Pre‐ and post‐supplementation samples were assessed in the same batch. Thawed PBMCs were washed and stimulated with LPS or PCSK for 30 min and stained for flow cytometry as described above.

## Results

### Vitamin D inhibition of innate immune responses in MDDC

**Figure 1:**
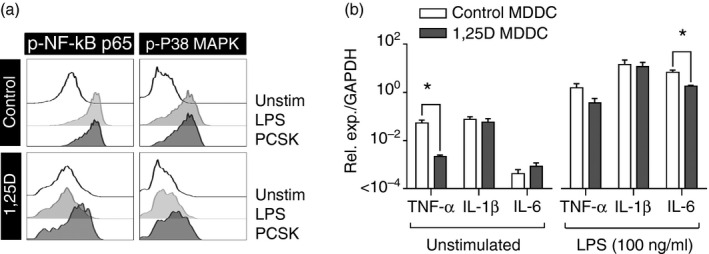
(a) Flow cytometry histograms of phospho‐nuclear factor‐*κ*B (NF *κ*B) p65 or phospho‐p38 mitogen‐activated protein kinase (MAPK) and (b) relative transcript abundance of the pro‐inflammatory cytokines indicated, in monocyte‐derived dendritic cells (MDDC) differentiated in the presence and absence of 100 nm 1,25[OH]D with and without stimulation of Toll‐like receptor 4 (TLR4) (LPS) or TLR2 (Pam_2_ CSK_4_) on day four. *n* = 3 per group. Bars represent mean ± SEM. *P < 0·05 by *t*‐test.

**Figure 2:**
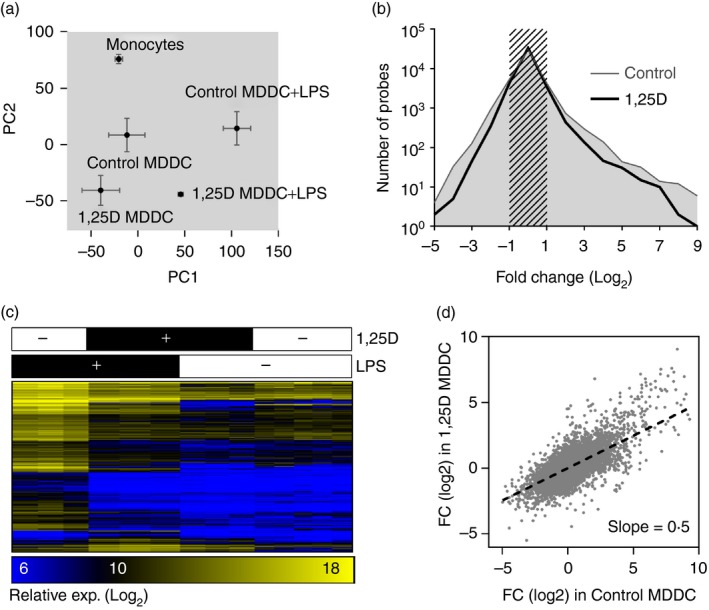
(a) Principal components analysis of genome‐wide transcriptomes of monocytes and monocyte‐derived dendritic cells (MDDC) differentiated in the presence or absence of 100 nm 1,25[OH]D with or without 4 hr of lipopolysaccharide (LPS) stimulation on day 4 (*n* = 3–8 per group. Data points represent mean ± SEM). (b) Frequency distribution of mean fold changes in transcript abundance, (c) heat map of mean gene expression in LPS up‐regulated genes in MDDC differentiated in the presence or absence of 100 nm 1,25[OH]D and (d) comparison of gene specific mean fold changes in response to 4 hr of LPS stimulation in MDDC differentiated in the presence or absence of 100 nm 1,25[OH]D.

Peripheral blood monocytes differentiated with IL‐4 and GM‐CSF for 4 days have been extensively used as an experimental model for myeloid DC,^2^ which respond to innate immune stimulation with widespread transcriptional changes involving both NF‐*κ*B and MAPK signalling pathways. Consistent with the existing literature,^7^, ^8^ we found that MDDC differentiated in the presence of 1,25[OH]D showed attenuated phosphorylation of NF‐*κ*B p65 and MAPK p38 in response to 30 min innate immune stimulation of TLR4 with LPS, or TLR2 with PCSK (Fig. 1a). Previous reports have suggested that this effect of vitamin D may underpin attenuated up‐regulation of T‐cell co‐stimulatory molecule expression by MDDC in response to innate immune stimulation and consequently contribute to vitamin D inhibition of DC‐mediated T‐cell activation.^7^ Counterintuitively, a recent report suggests that vitamin D preconditioning enhanced the expression of canonical pro‐inflammatory cytokines, such as TNF‐*α*, IL‐1*β* and IL‐6 following innate immune stimulation of MDDC with LPS for 4 hr.^14^ We observed that MDDC differentiation in the presence of 1,25[OH]D down‐regulated basal level expression of TNF‐*α* selectively, but transcription of all three cytokines was robustly up‐regulated in response to LPS stimulation. The only significant difference attributable to vitamin D was modest attenuation of IL‐6 transcriptional up‐regulation (Fig. 1b). In view of the fact that innate immune stimuli generate widespread transcriptional changes in DC,^15^ we extended this analysis to genome‐wide transcriptomes of MDDC differentiated in the presence and absence of 1,25[OH]D, with or without 4 hr LPS stimulation. We also compared these to the transcriptomes of monocytes from which the MDDC were derived. Principal component analysis (PCA) of these data showed transcriptomic changes in principal component 2 (PC2), attributable to differentiation of monocytes to MDDC and the effects of vitamin D, and independent changes in PC1 attributable to LPS stimulation (Fig. 2a). We concluded from this analysis that monocyte differentiation in response to GM‐CSF and IL‐4 is preserved in the presence of vitamin D consistent with another previous report.^16^ Interestingly, MDDC differentiated in the presence of 1,25[OH]D exhibited quantitatively smaller transcriptomic differences attributed to LPS stimulation in PC2, suggesting some attenuation of the response to LPS. Comparison of fold changes in gene expression following LPS stimulation, similarly showed narrowing of the frequency distribution in MDDC differentiated in the presence of 1,25[OH]D (Fig. 2b), suggesting that the range of LPS‐induced transcriptional changes is quantitatively attenuated by vitamin D This effect is shown in LPS up‐regulated genes specifically, at the level of individual genes (Fig. 2c, and see Supplementary material, Data S1), and by making genome‐wide comparison of quantitative fold changes in LPS‐inducible transcriptional changes, which gives a regression slope of 0·5 (Fig. 2d), and suggests that at the genome‐wide level, 1,25[OH]D reduced the magnitude of fold responses to LPS by half. In this analysis the *r* ^2^ correlation coefficient also approximated to 0·5, giving a measure of the gene‐specific heterogeneity of 1,25[OH]D attenuation in LPS responses.

### Vitamin D attenuation of NF‐*κ*B and p38 MAPK‐dependent transcriptional responses to LPS

**Figure 3:**
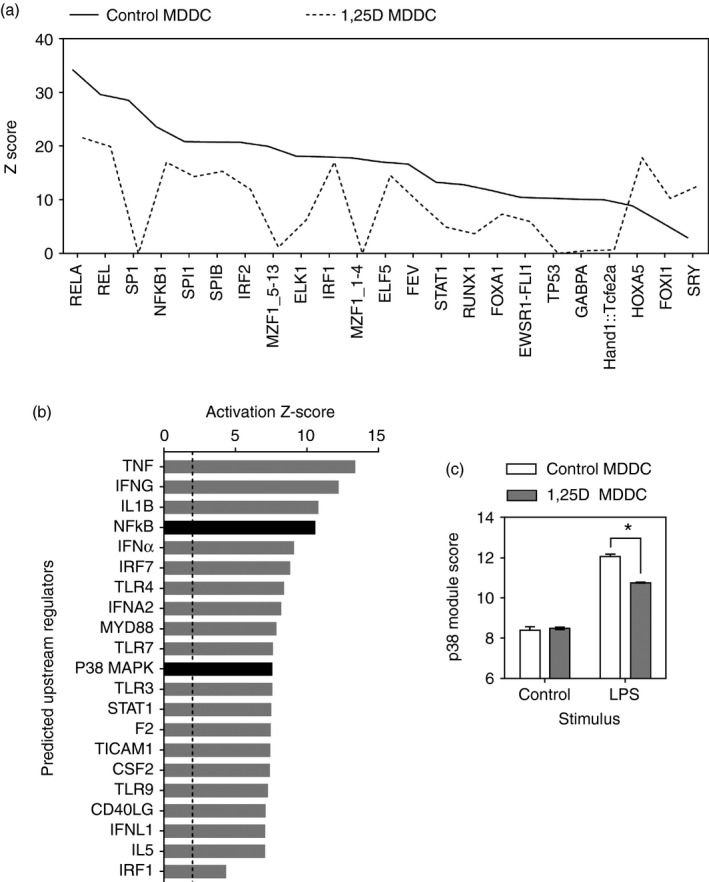
(a) Statistical enrichment (*Z* score) of transcription factor binding sites among genes up‐regulated by 4 hr of lipopolysaccharide (LPS) stimulation in monocyte‐derived dendritic cells (MDDC) differentiated in the absence or presence of 100 nm 1,25[OH]D (*n* = 3 per group). (b) Activation *Z*‐scores for upstream regulators of LPS up‐regulated genes in control MDDC after 4 hr of LPS stimulation. The dashed line denotes a *Z*‐score of 2, above which a regulator is predicted to be activated statistically. (c) Module score representing p38 mitogen‐activated protein kinase (MAPK) activity in control and 1,25[OH]D‐treated (1,25D) MDDC with or without 4 hr of LPS stimulation, derived from the geometric mean expression of genes that were identified as targets of p38 in the upstream analysis in (b). *n* = 3 in each group. Bars represent mean ± SEM. *P < 0·05 by *t*‐test.

Transcription factor binding site enrichment analysis of the genes up‐regulated by LPS can be used to infer the putative upstream transcriptional transactivator pathways.^12^ In control MDDC cells, this analysis was consistent with the predominant role of NF‐*κ*B pathways in LPS responses (Fig. 3a). The NF‐*κ*B transcription factor binding sites were also the most enriched in LPS responses by MDDC differentiated in the presence of 1,25[OH]D. In keeping with vitamin D inhibition of LPS‐induced NF‐*κ*B activation and the attenuation of transcriptional responses, statistical enrichment of NF‐*κ*B transcription factor binding sites in the list of LPS‐inducible genes was also lower in 1,25[OH]D‐exposed MDDC.

Assessment of the effect of vitaminD on MAPK p38‐dependent transcriptional responses by transcription factor binding site enrichment analysis was not possible because the MAPK pathway affects multiple downstream transcription factors. Instead we undertook a different bioinformatics approach using Ingenuity Pathway Analysis to show that MAPK p38 was predicted as an upstream regulator of LPS‐stimulated transcriptional responses in control DC (Fig. 3b). We then used the genes associated with p38 MAPK as an upstream regulator in this analysis to represent a gene expression module for p38 MAPK activity. The geometric mean expression of this module was significantly attenuated in MDDC differentiated in the presence of 1,25[OH]D (Fig. 3c) consistent with the hypothesis that vitamin D attenuated the activity of this pathway also.

### Differential effects of vitamin D on innate immune activation in myeloid cell subsets

Next, we sought to test the hypothesis that vitamin D inhibition of innate immune signalling pathways, which are evident in MDDC, are also observed in MDM, and in circulating blood monocytes and myeloid DC.

**Figure 4:**
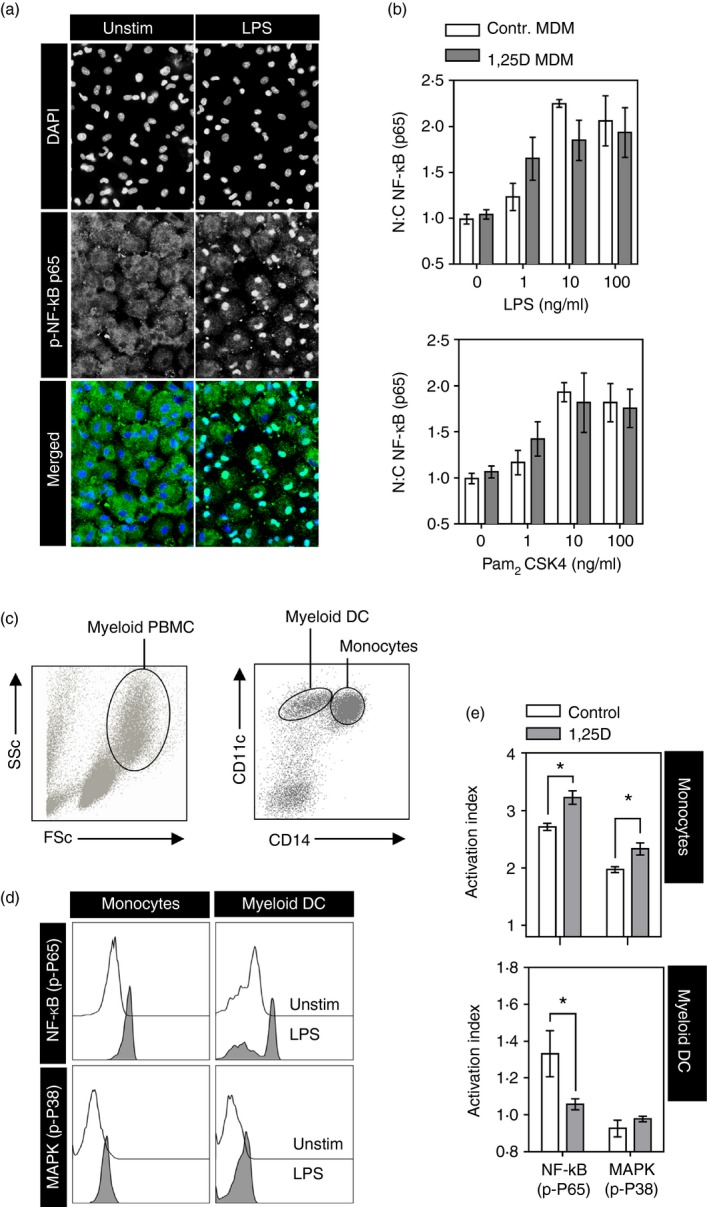
(a) Representative images of monocyte‐derived macrophages (MDM) stained with DAPI or antibody to nuclear factor‐*κ*B (NF‐*κ*B) p65 and (b) summary data from > 500 single cell measurements of nuclear : cytoplasmic ratios of NF‐*κ*B p65 staining in MDM differentiated in the presence and absence of 100 nm 1,25[OH]D following 60 min of stimulation with increasing concentrations of lipopolysaccharide (LPS) or Pam_2_ CSK_4_. Bars represent mean ± SEM. (c) Representative flow cytomeytric dot plots to show gating strategy for identification of myeloid cells on the basis of light scatter and then monocytes and myeloid dendritic cells (DC) on the basis of CD11c and CD14 immunostaining. (d) Representative histograms of flow cytometric measurement of cell‐type‐specific phospho‐NF‐*κ*B p65 or p38 mitogen‐activated protein kinase (MAPK) staining in peripheral blood mononuclear cells (PBMC) with and without 30 min of LPS stimulation. (e) Summary data for activation of NF‐*κ*B and MAPK pathways measured by ratio of phospho‐protein staining in stimulated to unstimulated cells, in PBMC with (grey bars) and without (white bars) prior overnight incubation with 100 nm 1,25[OH]D (1,25D). *n* = 5 per group. Bars represent mean ± SEM. *P < 0·05 by *t*‐test.

The MDM were differentiated with M‐CSF in the presence and absence of 1,25[OH]D for 6 days as previously described.^2^ Canonical NF‐*κ*B pathway activation in response to LPS or PCSK was then assessed in this model using a well‐established microscopy assay to quantify nuclear translocation of NF‐*κ*B p65.^13^, ^17^, ^18^ These cells showed a clear dose response to both stimuli but no attenuation of NF‐*κ*B activation in MDM exposed to vitamin D (Fig. 4a,b).

To investigate the effect of vitamin D on peripheral blood monocytes and myeloid DC, PBMC were incubated with 1,25[OH]D overnight before stimulation with LPS for 30 min. An activation index for NF‐*κ*B and MAPK pathways was then quantified by flow cytometric comparison of phosphorylated (p)‐NF‐*κ*B p65 or p‐p38 MAPK in stimulated and unstimulated cells identified by light scatter properties and immunostaining for CD11c and CD14 (Fig. 4c), where monocytes were identified as CD14^hi^ CD11^hi^ and circulating myeloid DC as CD14^lo^ CD11c^hi^. In this analysis, we detected increased NF‐*κ*B p65 phosphorylation, but no increase in p38 MAPK phosphorylation in myeloid DC following stimulation with LPS. The NF‐*κ*B response was inhibited in peripheral blood myeloid DC after overnight exposure to 1,25[OH]D (Fig. 4d,e), consistent with the inhibitory effect of vitamin D on innate immune stimulation of MDDC. In contrast, phosphorylation of both NF‐*κ*B p65 and p38 MAPK was significantly enhanced in peripheral blood monocytes exposed to 1,25[OH]D (Fig. 4d,e). Hence pre‐incubation with vitamin D had the opposite effect on innate immune response signalling in blood monocytes and myeloid DC.

### Opposing effects of *in vivo* vitamin D supplementation in peripheral blood monocytes and myeloid DC

**Figure 5:**
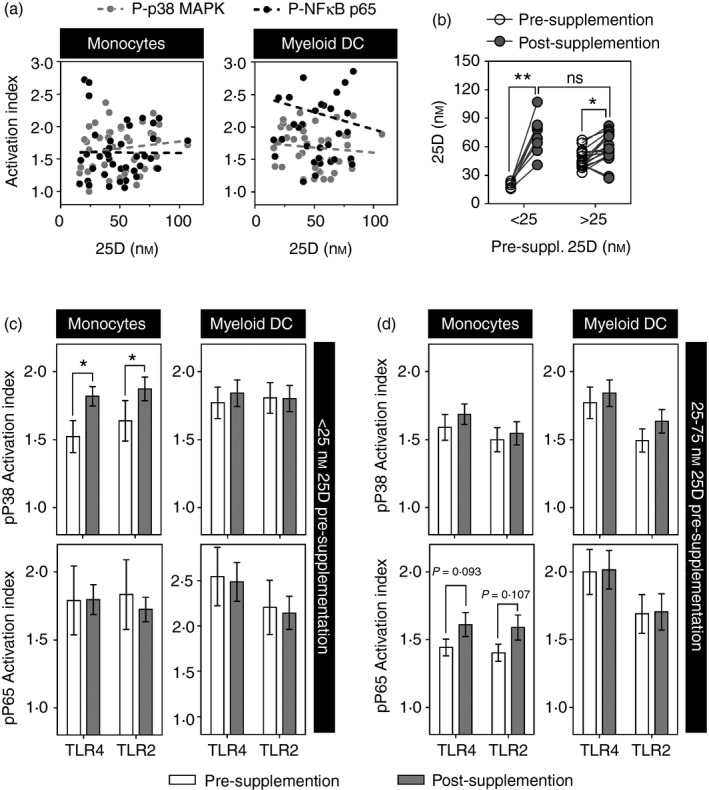
(a) Cross‐sectional relationship between activation index for phosphorylation of nuclear factor‐*κ*B (NF‐*κ*B) p65 and p38 mitogen‐activated protein kinase (MAPK) within monocytes and myeloid dendritic cells (DC) in peripheral blood mononuclear cells (PBMC) stimulated with lipopolysaccharide (LPS), and serum levels of 25[OH]D (25D). All correlation coefficients *P* > 0·05. (b) Serum 25[OH]D levels before and after oral vitamin D supplementation in subjects with < 25 nm (*n* = 8) or ≥ 25 nm (*n* = 18) pre‐supplementation. Paired data points shown for each subject. NS denotes non‐significance, *P = 0·008 and **P = 0·002 by Wilcoxon signed rank test. (c) Activation index for phosphorylation of NF‐*κ*B p65 and p38 MAPK within monocytes and myeloid DC in PBMC stimulated with LPS [Toll‐like receptor 4 (TLR4)] or Pam_2_ CSK4 (TLR2) before and after oral vitamin D3 supplementation in deficient subjects with < 25 nm or (d) insufficient subjects with 25–75 nm pre‐supplementation. *n* = 8 per group. Bars represent mean ± SEM. *P < 0·05 by *t*‐test.

The opposing effects of *ex vivo* exposure of circulating blood monocytes and myeloid DC to 1,25[OH]D has not previously been reported. Therefore, we sought to validate and extend these observations in a more physiological context, by testing the hypothesis that vitamin D levels *in vivo* also show positive correlation with enhanced innate immune signalling in monocytes and attenuated signalling in circulating myeloid DC. Circulating 1,25[OH]D is predominantly protein‐bound and the total levels do not accurately reflect total vitamin D levels *in vivo*. Instead, circulating levels of the precursor, 25[OH]D, are more commonly used as a measure of vitamin D levels in blood. In a cross‐sectional study of patients subjected to vitamin D testing, we found no correlations between variation in serum 25[OH]D levels and variation in innate immune activation indices for phosphorylation of NF‐*κ*B p65 or p38 MAPK in monocytes or myeloid DC within *ex vivo* PBMC stimulated with LPS (Fig. 5a).

Patients with serum 25[OH]D levels below 25 nm (10 ng/ml) are considered deficient, and vitamin D supplementation is mandated in this group. Those with levels between 25 and 75 nm (10–30 ng/ml) are considered vitamin D insufficient, with supplementation optional depending on the patient's co‐morbidities and symptoms. The aim of supplementation is to achieve levels of > 75 nm (30 ng/ml), according to published guidelines.^19^ Vitamin D supplementation in this context, therefore provided an opportunity to assess the effect of vitamin D on innate immune signalling in monocytes and circulating myeloid DC in samples obtained before and after successful oral supplementation. For this, we recruited adults with vitamin D deficiency. We first tested innate immune responses in PBMC from patients in whom pre‐supplementation 25[OH]D levels were < 25 nm, representing vitamin D deficiency. All of these patients showed increased 25[OH]D levels after vitamin D3 supplementation. In keeping with our results from *in vitro* incubation of PBMC to 1,25[OH]D, we found that successful *in vivo* supplementation with vitamin D3 in this group was associated with enhanced LPS‐induced phosphorylation of p38 MAPK in monocytes, but we found no significant effects on LPS‐induced phosphorylation of NF‐*κ*B p65 in monocytes, or on either signalling event in circulating myeloid DC (Fig. 5c). We also tested patients in whom pre‐supplementation 25[OH]D levels were > 25 nm. In this group, we found no differences in the target NF‐*κ*B or MAPK signalling events in either the monocytes or myeloid DC within PBMC (Fig. 5d), suggesting that an effect of vitamin D supplementation is only detectable in those with vitamin D deficiency (< 25 nm) before receiving oral supplements.

## Discussion

Vitamin D has been consistently shown to inhibit maturation of MDDC following innate immune stimulation and consequently their ability to activate T cells. We have confirmed that vitamin D inhibits NF‐*κ*B and MAPK signalling in MDDC following innate immune stimulation. This was associated with a global attenuation of LPS‐induced transcriptional responses. We could not replicate a previous report that pro‐inflammatory innate immune responses were augmented by vitamin D.^14^ The inhibitory effects of *in vitro* vitamin D treatment on innate immune signalling was reproduced in primary peripheral blood myeloid DC, but not in monocytes within the same PBMC samples or in MDM. In fact, we observed increased innate immune activation of both NF‐*κ*B and MAPK pathways in monocytes within PBMC following *in vitro* incubation with vitamin D. At face value, this observation also contrasts with a previous report that *in vitro* incubation of monocytes with vitamin D does inhibit innate immune signalling and transcriptional responses, possibly as a result of down‐regulated expression of innate immune receptors such as TLR2 and TLR4.^20^ Interestingly, however, the effect of vitamin D in this earlier report was most evident with increasing time in culture, raising the possibility that the observations may have been confounded by *in vitro* cellular differentiation of the monocytes in culture. Further paired assessments of peripheral blood monocytes and myeloid DC from vitamin D‐deficient subjects before and after oral vitamin D3 supplementation provided a unique opportunity to assess *in vivo* modulation of innate immune activation pathways by vitamin D. In these experiments, we found increased innate immune activation of the MAPK pathway in monocytes, but no effect on NF‐*κ*B activation and no effect in peripheral blood myeloid DC. These effects were specific to the subgroup of patients with baseline 25[OH]D levels < 25 nm and not evident in those with baseline 25[OH]D levels of 25‐75 nm, suggesting that vitamin D supplementation to modulate innate immune signalling may only be effective in people with severe deficiency.

Taken together, we conclude that although vitamin D undoubtedly can modulate innate immune activation pathways in myeloid cells, its effects are highly cell‐specific. Most importantly, our data highlight that translational applications in which vitamin D supplementation is being suggested on the basis of the existing literature in DC, may actually cause the opposite effect, particularly in diseases in which monocytes or macrophages play a dominant role. Further, immunological assessments before and after vitamin D supplementation are needed to inform context‐specific immunomodulatory interventions with vitamin D specifically targeting those with pre‐existing deficiency with baseline levels < 25 nm.

## Disclosures

The authors declare no competing interests.

## Supporting information


**Data S1.** LPS upregulated genes in vitamin D exposed and control dendritic cells.
