# Supplementary material for: Cell‐type‐specific modulation of innate immune signalling by vitamin D in human mononuclear phagocytes
Source: Immunology. 2016 Oct 3;150(1):55–63. doi: 10.1111/imm.12669 (PMC5167305; doi:10.1111/imm.12669)
Supplement: Supplementary file 1 — Data S1. LPS upregulated genes in vitamin D exposed and control dendritic cells. [file IMM-150-55-s001.pdf]

Agilent probe name	REFSEQ	GENE_SYMBOL	Relative gene expr. Unstimulated control MDDC	Relative gene expr. LPS stimulated control MDDC	Relative gene expr. Unstimulated vitamin D treated MDDC	Relative gene expr. LPS stimulated vitamin D treated MDDC	LPS induced fold change in control MDDC	LPS induced fold change in vitamin D treated MDDC
A_33_P3284933	NM_145659	IL27	6.363339487	15.72255282	8.993049444	12.70494129	9.359213329	3.711891843
A_23_P82868	NM_000930	PLAT	5.438353855	14.61734328	6.753300436	10.82495975	9.178989425	4.071659314
A_23_P49759	NM_002981	CCL1	6.794495464	15.9501778	6.883605873	12.51698761	9.15568234	5.633381737
A_23_P71037	NM_000600	IL6	7.419472754	16.11085089	7.566215937	16.61817509	8.691378132	9.051959153
A_23_P121064	NM_002852	PTX3	8.371675373	17.02900941	8.0071617	14.98847579	8.657334034	6.981314093
A_23_P125278	NM_005409	CXCL11	6.381037431	14.95104689	5.623800401	8.172601018	8.570009455	2.548800617
A_23_P337800	NM_172140	IL29	6.371942634	14.74538307	5.84643548	8.754479564	8.373440439	2.908044084
A_23_P7144	NM_001511	CXCL1	7.694692545	16.05710055	5.460196508	12.77810807	8.362408002	7.317911566
A_23_P72096	NM_000575	IL1A	5.54993371	13.88765459	6.970191829	11.93549712	8.337720877	4.965305295
A_23_P139786	NM_003733	OASL	7.839273783	16.09274851	7.776560445	14.38514296	8.253474727	6.608582515
A_24_P303091	NM_001565	CXCL10	7.683343747	15.92737936	6.18504512	11.43468732	8.244035613	5.249642197
A_23_P7560	NM_002187	IL12B	6.242479321	14.36314816	5.668196021	8.783422359	8.120668836	3.115226338
A_33_P3342628	NM_021170	HES4	7.982766778	16.09088124	7.329548719	13.29960381	8.108114462	5.970055087
A_23_P17065	NM_004591	CCL20	5.655927349	13.6232967	5.655900517	13.25068234	7.967369354	7.594781823
A_23_P165624	NM_007115	TNFAIP6	7.466994621	15.28554188	8.580632981	15.60636831	7.818547259	7.025735329
A_32_P9543	NM_145699	APOBEC3A	6.232091418	13.97789149	6.784102322	10.34364849	7.745800076	3.559546163
A_23_P328740	NR_026875	NEURL3	6.906854054	14.60708943	5.650116323	11.50933729	7.700235376	5.85922097
A_23_P152838	NM_002985	CCL5	8.40839977	16.07708336	10.66945372	14.01038434	7.668683587	3.340930617
A_24_P304071	NM_001547	IFIT2	6.441472255	14.03075882	6.6941106	12.68617966	7.589286565	5.99206906
A_23_P76078	NM_016584	IL23A	7.253063839	14.47678545	8.835331096	16.36084399	7.223721608	7.525512898
A_23_P71774	NM_002176	IFNB1	5.346384551	12.54066309	5.535874351	6.812300146	7.194278535	1.276425795
A_33_P3397763	NM_003811	TNFSF9	6.123604413	13.31565552	8.170397607	10.94945044	7.192051107	2.779052829
A_33_P3619171	NM_021127	PMAIP1	8.032574722	15.21242053	7.571602566	13.47199188	7.179845805	5.90038931
A_24_P28722	NM_080657	RSAD2	7.91174737	15.07594528	6.811746739	12.8376561	7.16419791	6.025909358
A_24_P250922	NM_000963	PTGS2	5.732367344	12.84483989	6.490256428	13.64328028	7.112472549	7.153023855
A_32_P87013	NM_000584	IL8	7.079207839	13.99019399	8.26018103	13.68624246	6.910986154	5.426061427
A_33_P3283611	NM_001549	IFIT3	9.049127928	15.87641288	8.576596909	14.72410915	6.827284956	6.147512237
A_33_P3386547	NM_152386	SGPP2	5.820554664	12.55991371	5.444211732	10.03642376	6.73935905	4.592212027
A_23_P110196	NM_016323	HERC5	7.418715189	14.01212378	7.371603826	12.09855346	6.593408591	4.726949638
A_23_P18452	NM_002416	CXCL9	8.697719559	14.83848712	6.1821261	6.785902626	6.140767564	0.603776526
A_24_P66027	NM_004900	APOBEC3B	7.051800071	13.18621374	7.120449384	9.963360647	6.134413665	2.842911262
A_23_P132159	NM_017414	USP18	7.586152082	13.70079966	6.89141796	10.23613736	6.114647582	3.344719402
A_23_P52266	NM_001548	IFIT1	10.29479476	16.39932283	7.678653497	14.003077	6.104528066	6.324423499
A_33_P3418170	NM_014314	DDX58	8.294803247	14.38601636	6.79132308	10.85436684	6.091213117	4.063043763
A_23_P32404	NM_002201	ISG20	9.870881547	15.94966216	8.099898758	13.53369643	6.078780613	5.433797672
A_23_P386478	NM_024873	TNIP3	5.245294129	11.30615351	5.492325459	11.76807419	6.060859385	6.275748731
A_32_P108156	NR_001458	MIR155HG	6.649903117	12.70254307	6.59731156	9.747613749	6.052639956	3.150302189
A_23_P60146	NM_006207	PDGFRL	5.337493943	11.29622289	5.493513385	8.599293696	5.958728944	3.105780311
A_33_P3352970	NM_001570	IRAK2	7.178387984	13.11985874	6.803961862	11.82858718	5.941470753	5.024625321
A_33_P3319925	XM_001133269	IRG1	5.812731087	11.74281623	5.83467765	12.36246999	5.930085147	6.52779234
A_23_P62647	NM_003037	SLAMF1	8.716046679	14.63706442	7.489481803	10.97757468	5.921017741	3.48809288
A_24_P167642	NM_000161	GCH1	6.438451729	12.32686683	6.798317214	11.29996131	5.888415105	4.501644096
A_23_P131846	NM_005985	SNAI1	6.104934572	11.97415925	6.049551769	9.649028538	5.869224678	3.599476769
A_23_P94754	NM_005118	TNFSF15	5.564606016	11.39827219	5.726821379	9.026819024	5.833666174	3.299997645
A_23_P79518	NM_000576	IL1B	10.8724131	16.69907869	10.4141935	17.2239084	5.826665586	6.809714903
A_23_P122924	NM_002192	INHBA	10.07763743	15.8179495	13.38969674	15.93833806	5.740312063	2.548641323
A_24_P237270	NM_000675	ADORA2A	5.679018851	11.41080277	5.842566638	9.729603445	5.731783916	3.887036807
A_23_P74609	NM_015714	G0S2	10.32870797	16.04694642	12.19840441	16.54898536	5.718238447	4.350580947
A_33_P3243449	NM_001252	CD70	6.541579968	12.24163158	6.599862881	6.977397117	5.700051612	0.377534237
A_23_P356616	NM_145804	ABTB2	8.030981536	13.71977519	6.430670681	10.97178421	5.688793654	4.541113526
A_24_P183150	NM_002090	CXCL3	6.713583647	12.36055121	5.511201636	10.9595575	5.646967566	5.448355861
A_23_P376488	NM_000594	TNF	10.78485103	16.30005347	8.421714964	15.58449492	5.515202443	7.162779956
A_23_P169092	NM_004820	CYP7B1	5.526355314	11.04034355	5.779515768	7.566848083	5.513988232	1.787332315
A_33_P3381671		LOC731424	6.065454566	11.53610906	7.613810935	13.0559667	5.470654494	5.442155765
A_24_P89891	NM_005658	TRAF1	9.229345798	14.69806144	6.873564129	12.97517813	5.468715639	6.101614004
A_23_P23639	NM_153259	MCOLN2	8.106168788	13.54096655	6.351413773	11.06032083	5.434797765	4.708907054
A_23_P112026	NM_002164	IDO1	9.534665773	14.95449556	8.016287195	12.34165345	5.419829784	4.325366255
A_23_P152002	NM_004049	BCL2A1	9.489133135	14.85723471	13.49360081	14.68206037	5.368101578	1.188459567
A_23_P45871	NM_006820	IFI44L	7.670595003	13.01988508	6.131137732	9.415881234	5.349290077	3.284743502
A_24_P759477	NM_002214	ITGB8	5.866851636	11.12920875	7.22877503	11.39008539	5.262357117	4.161310357
A_24_P23625		HS3ST3B1	5.815095104	11.05759539	6.091757316	8.958647674	5.242500286	2.866890358
A_33_P3227899	NR_015361	LOC440896	8.397062813	13.59912436	6.377591093	9.421086156	5.202061551	3.043495063
A_33_P3401826	NM_207315	CMPK2	9.535861259	14.72815345	7.68631437	11.94654745	5.192292187	4.26023308
A_33_P3372742		KRTAP5-11	10.1612611	15.32373988	9.702374704	14.96689695	5.162478777	5.264522249
A_33_P3296181	NM_001001437	CCL3L3	10.9159133	16.07260553	10.57635365	16.341786	5.156692225	5.765432347
A_24_P295010	NM_004155	SERPINB9	8.179335643	13.33120827	7.882216228	11.04515356	5.151872624	3.162937329
A_23_P138680	NM_172200	IL15RA	7.424277103	12.57604775	7.68777694	10.41708819	5.151770647	2.729311254
A_23_P109034	NM_002999	SDC4	9.463488053	14.59712931	10.53242228	12.81289785	5.133641253	2.280475573
A_23_P207456	NM_005623	CCL8	7.623148298	12.7174948	6.620633491	12.71249031	5.094346505	6.091856823
A_24_P320033	NM_005191	CD80	7.350280992	12.40853524	6.093196323	9.530270033	5.058254248	3.43707371
A_23_P62890	NM_002053	GBP1	8.658265757	13.70274496	7.953528173	11.98474404	5.044479203	4.031215863
A_23_P359277	NM_024930	ELOVL7	5.646644878	10.67696016	5.693376543	10.68197812	5.030315285	4.988601581
A_23_P150693	NM_014344	FJX1	7.10713163	12.10683117	6.500988156	8.206524455	4.99969954	1.705536299
A_23_P97402	NM_020439	CAMK1G	5.833907924	10.67131612	5.409870394	6.612928787	4.837408192	1.203058394
A_23_P2283	NM_013251	TAC3	5.297255225	10.11279985	5.550996769	5.718529836	4.81554463	0.167533067
A_33_P3316928	NM_020651	PELI1	10.0338724	14.8069717	9.630141414	13.55182969	4.773099301	3.921688276
A_33_P3381235	NR_024470	LOC100127888	7.919588408	12.67965343	7.763539803	12.11699651	4.760065026	4.353456707
A_33_P3304668	NM_000088	COL1A1	5.511274648	10.23073229	5.690884077	5.711485572	4.719457645	0.020601496
A_24_P114249	NM_004482	GALNT3	5.479704935	10.18996918	5.542561446	6.871592076	4.710264244	1.32903063
A_23_P315364	NM_002089	CXCL2	9.369630573	14.05504809	6.246360751	11.68563968	4.685417521	5.439278929
A_23_P74290	NM_052942	GBP5	10.56359398	15.17775834	9.106272497	12.49143056	4.614164363	3.385158067
A_33_P3286157	NM_003327	TNFRSF4	10.13174681	14.74543148	6.592685463	10.82556428	4.613684664	4.23287882
A_23_P388993	NM_033390	ZC3H12C	7.530215261	12.13387263	7.232258594	11.33078947	4.603657369	4.098530879
A_23_P150018	NM_004419	DUSP5	9.47944797	14.07828369	8.802465003	11.7002709	4.59883572	2.8978059
A_24_P157926	NM_006290	TNFAIP3	9.182865029	13.75781915	9.336251977	12.56135365	4.574954118	3.225101677
A_23_P23947	NM_005204	MAP3K8	9.167811984	13.73570951	8.2388811	10.95901601	4.567897523	2.720134907
A_32_P54553	XM_036729	USP41	5.709988775	10.25880247	5.684750239	7.340254884	4.548813691	1.655504645
A_33_P3329974	NM_020770	CGN	6.128010242	10.66797938	5.675534843	5.764010379	4.539969135	0.088475536
A_24_P45446	NM_052941	GBP4	8.627253544	13.1669708	8.442752768	10.31218111	4.539717256	1.869428346
A_33_P3236881	NM_001204088	C1orf151-NBL1	8.560139102	13.08633874	9.599176074	9.191478391	4.526199635	-0.407697684
A_23_P167328	NM_001775	CD38	8.511438825	13.0327237	7.599483265	10.89509125	4.521284872	3.295607985
A_23_P28334	NM_003853	IL18RAP	5.651618195	10.15059368	5.486537997	5.907041394	4.498975489	0.420503397
A_23_P133408	NM_000758	CSF2	5.251639449	9.749246004	5.398914768	6.415075279	4.497606554	1.016160511
A_23_P68031	NM_003151	STAT4	7.570470136	12.06185834	6.34560395	9.279606967	4.491388204	2.934003016
A_23_P207058	NM_003955	SOCS3	10.00446082	14.49284672	10.33752593	13.2401289	4.488385896	2.902602977
A_33_P3262495	NM_032772	ZNF503	7.464257511	11.94866487	7.148662603	8.451547329	4.484407359	1.302884726
A_33_P3295358	NM_139314	ANGPTL4	7.469591878	11.94713935	10.13085486	13.49754748	4.477547469	3.366692617
A_24_P408047	NM_020904	PLEKHA4	6.780711314	11.25701322	8.497515427	8.259898231	4.476301902	-0.237617196
A_23_P51936	NM_001561	TNFRSF9	5.953079893	10.42671718	5.776820559	7.544084552	4.473637285	1.767263993
A_23_P209394	NM_001127184	CFLAR	10.58625743	15.05592905	10.91559498	13.61216507	4.469671623	2.696570085

A_23_P43273	NM_000127	EXT1	7.270041865	11.73172651	6.536085498	9.527895584	4.461684642	2.991810086
A_23_P157865	NM_002160	TNC	6.840540977	11.29736641	7.192150736	11.29059136	4.456825433	4.098440627
A_23_P127288	NM_000417	IL2RA	5.512819649	9.953390856	5.768812938	7.274382254	4.440571206	1.505569316
A_32_P74409	NM_001145033	C11orf96	5.576921317	10.01645397	5.443739745	9.181571361	4.43953265	3.737831616
A_23_P501754	NM_000759	CSF3	4.956791236	9.396316923	5.320863427	9.587736114	4.439525687	4.266872687
A_23_P119478	NM_005755	EBI3	8.49476932	12.93144989	5.874894572	8.497720452	4.436680573	2.62282588
A_33_P3224070	NM_033027	CSRNP1	7.049946448	11.45908983	7.783715711	9.88663837	4.409143378	2.102922659
A_23_P169257	NM_001244	TNFSF8	6.530356896	10.91771894	6.424967085	8.126077311	4.387362048	1.701110226
A_33_P3375934	NM_005746	NAMPT	9.556306062	13.92282323	8.29278678	11.28404479	4.366517164	2.991258013
A_33_P3245439	NM_001250	CD40	10.0316101	14.38339358	9.377794705	10.76344977	4.351783478	1.385655062
A_23_P46470	NM_018948	ERRFI1	7.290722926	11.60013119	7.198985783	7.641429558	4.309408264	0.442443774
A_23_P320578	NM_002928	RGS16	6.795192795	11.04724344	8.494150962	8.846202994	4.252050645	0.352052032
A_23_P121106	NM_003865	HESX1	6.072283516	10.32341912	5.7708559	6.628388491	4.251135607	0.857532591
A_23_P17663	NM_002462	MX1	11.46342256	15.70903513	9.550162122	13.75822268	4.245612575	4.208060555
A_23_P156687	NM_001710	CFB	8.105378027	12.31706091	8.587749767	13.55367448	4.211682886	4.965924713
A_33_P3226810	NM_003810	TNFSF10	8.981769514	13.18796121	9.352435524	9.544689269	4.206191699	0.192253746
A_23_P251075	NM_005491	MAMLD1	6.380898421	10.57625075	5.760209866	7.170333416	4.195352333	1.41012355
A_23_P57784	NM_021101	CLDN1	8.032704253	12.22216517	5.539205184	5.719849585	4.189460914	0.180644401
A_23_P52647	NM_006795	EHD1	10.99913345	15.181216	10.42817556	14.67293215	4.182082546	4.244756583
A_23_P167599	NM_001034850	FAM134B	6.281874315	10.45114535	7.23257212	8.968389553	4.169271035	1.735817433
A_23_P819	NM_005101	ISG15	12.19757982	16.36021262	10.42648264	15.3898805	4.162632793	4.963397861
A_33_P3415052	NM_001172292	NIPAL4	5.532415205	9.688781559	7.697891556	10.32000096	4.156366354	2.6221094
A_33_P3226832	NM_001993	F3	7.734408153	11.87617787	9.70064704	12.70129411	4.141769714	3.000647067
A_23_P259071	NM_001657	AREG	6.957039678	11.08212697	5.814831201	7.225501124	4.125087287	1.410669923
A_24_P353638	NM_021181	SLAMF7	12.34838086	16.46985864	11.9808575	15.56321347	4.121477787	3.582355963
A_33_P3423941	NM_003641	IFITM1	7.822595884	11.94107372	6.399922797	8.990391648	4.118477836	2.590468851
A_23_P329261	NM_000891	KCNJ2	9.175500429	13.29269534	7.150650433	8.04196722	4.117194908	0.891316787
A_23_P138760	NM_013246	CLCF1	6.498807337	10.605628	8.058880498	10.39509665	4.106820666	2.336216148
A_23_P150394	NM_022003	FXYD6	6.918730962	11.00127146	9.07759811	10.13881865	4.082540494	1.06122054
A_33_P3381513	NM_014143	CD274	7.361693446	11.44382194	7.66474608	10.22631923	4.082128494	2.561573153
A_23_P44724	NM_001321	CSRP2	6.861522867	10.8893747	8.846652077	8.6976715	4.027851833	-0.148980577
A_32_P219368	NM_152858	WTAP	8.593805807	12.59577556	8.698166058	12.09261737	4.001969756	3.394451309
A_23_P134176	NM_001024465	SOD2	12.39065579	16.38957992	13.18734315	16.72585813	3.998924131	3.53851498
A_23_P23074	NM_006417	IFI44	10.75817019	14.75468801	8.965389121	13.01690928	3.996517828	4.051520156
A_23_P25674	NM_001823	CKB	8.414573328	12.4079422	9.870684583	11.62284557	3.993368869	1.752160984
A_23_P6263	NM_002463	MX2	10.60427091	14.58456637	9.530098742	12.07649193	3.980295456	2.546393188
A_24_P286114	NM_004172	SLC1A3	9.869699118	13.84356078	10.48386528	13.50842948	3.973861659	3.024564197
A_23_P151975	NM_016321	RHCG	5.763369939	9.734148344	5.762359686	8.247016882	3.970778405	2.484657196
A_23_P334870	NM_145316	TMEM217	7.046569395	11.01689842	7.107670937	8.956330637	3.970329029	1.8486597
A_23_P131024	NM_014383	ZBTB32	5.621852649	9.592026048	5.84181908	6.136244989	3.970173399	0.294425908
A_32_P44394	NM_004833	AIM2	7.502290834	11.4671767	7.225906601	9.91632482	3.964885863	2.690418219
A_24_P37409	NM_004418	DUSP2	8.551254823	12.51094282	8.655672369	8.32885855	3.959687993	-0.326813818
A_23_P68155	NM_022168	IFIH1	11.1972195	15.15115882	10.44240006	13.05442354	3.953939316	2.612023476
A_33_P3214720	NM_025079	ZC3H12A	9.694936986	13.63949184	10.05219787	13.8956416	3.944554854	3.843443726
A_33_P3314974	NR_028339	LOC100130522	7.460629258	11.37861831	7.621217198	11.06839657	3.917989055	3.44717937
A_23_P409438	NM_172138	IL28A	7.294971177	11.20502555	7.191641504	8.377507603	3.910054372	1.185866099
A_24_P239731	NM_004776	B4GALT5	9.712555355	13.61973149	10.65907761	12.75124411	3.907176132	2.0921665
A_23_P50000	NM_024792	FAM57A	6.200428277	10.10044203	6.145274508	7.693333733	3.900013749	1.548059225
A_23_P250607	NM_005032	PLS3	4.999913347	8.89877367	5.371529443	6.894438104	3.898860323	1.522908661
A_23_P204947	NM_004004	GJB2	6.965194807	10.86214885	8.144034728	11.12318039	3.896954043	2.979145662
A_23_P42353	NM_016135	ETV7	5.857441697	9.754304021	5.905347792	6.481980377	3.896862324	0.576632585
A_23_P167017	NM_022135	POPDC2	7.18783072	11.07794985	6.568095967	7.581782664	3.890119134	1.013686696
A_24_P214598	NM_152542	PPM1K	8.363138208	12.24382575	7.738429036	9.630123783	3.880687545	1.891694747
A_24_P33895	NM_001040619	ATF3	7.727875374	11.59802704	9.502892652	11.67484371	3.870151666	2.171951055
A_32_P133840	NM_014858	TMCC2	8.475579719	12.3185229	7.27811443	7.463619498	3.842943185	0.185505068
A_23_P404494	NM_002185	IL7R	12.05681729	15.89249946	6.516887249	10.38246787	3.835682172	3.865580617
A_33_P3264895	NM_144593	RHEBL1	8.388860199	12.184081	7.899185352	10.26148482	3.795220804	2.362299465
A_33_P3393836	NM_001002010	NT5C3	8.876207449	12.6695396	9.806066864	10.42035946	3.793332155	0.614292595
A_23_P398294	NM_003959	HIP1R	6.475952883	10.267818	6.587472137	7.2981678	3.791865114	0.710695663
A_33_P3272231	NM_001136493	MFSD2A	8.380690559	12.16974738	9.786308973	12.7800979	3.789056821	2.993788923
A_24_P389916	NM_005512	LRRC32	6.229750815	10.00439009	12.47897775	13.15571491	3.77463928	0.676737167
A_23_P500130	NM_153186	KANK1	6.205101358	9.958421325	6.015013405	10.44321013	3.753319967	4.428196724
A_23_P128808	NM_013345	GPR132	9.199020577	12.9481634	8.50891076	11.06000093	3.749142826	2.551090167
A_23_P136405	NM_005018	PDCD1	8.82131871	12.56142162	7.767481595	8.579095246	3.740102907	0.811613651
A_23_P212089	NM_031419	NFKBIZ	6.774780241	10.50057428	6.703029245	9.197099956	3.725794043	2.494070711
A_23_P427703	NR_001447	MT1L	10.67125795	14.38837205	15.80171798	14.27569335	3.717114102	-1.52602463
A_23_P214766	NM_006734	HIVEP2	6.625204717	10.34022273	7.166299462	11.51434641	3.715018015	4.348046944
A_23_P26024	NM_032413	C15orf48	12.35978346	16.07057192	13.01615588	16.53201423	3.710788455	3.51585835
A_23_P30024	NM_003998	NFKB1	11.27429896	14.9848834	9.94270729	14.05121437	3.710584438	4.10850708
A_23_P106844	NM_005953	MT2A	12.49718672	16.20675613	17.2844461	15.92855297	3.709569408	-1.355893133
A_33_P3329013	NM_001050	SSTR2	5.381348868	9.087185446	5.635177971	6.110771934	3.705836578	0.475593963
A_23_P105794	NM_033255	EPSTI1	8.166937097	11.87146109	6.128973853	8.773624712	3.70452399	2.644650859
A_24_P258051	NM_032844	MASTL	6.451169272	10.15509555	7.17050522	8.796399708	3.703926274	1.625894487
A_33_P3354607	NM_002984	CCL4	12.79810121	16.49714766	11.5383254	17.25872928	3.699046449	5.72040388
A_24_P49190	NM_181655	C17orf58	8.11218377	11.78262012	8.093678715	10.28726333	3.670436353	2.193584618
A_33_P3263867	NM_002562	P2RX7	10.4799469	14.14623721	10.87938509	13.0895799	3.666290306	2.210194818
A_23_P98350	NM_001165	BIRC3	11.69043873	15.34755349	6.906880922	11.41766806	3.657114761	4.510787141
A_24_P506977	NR_003697	C7orf40	9.949652592	13.59929286	10.92131534	13.52033318	3.649640272	2.599017833
A_23_P53370	NM_014470	RND1	5.541520789	9.182128005	5.660149365	6.094420807	3.640607217	0.434271442
A_23_P161769	NM_021603	FXYD2	7.458900965	11.09225679	10.39950198	11.31168816	3.633355825	0.912186182
A_23_P96158	NM_000422	KRT17	7.406036601	11.02253592	5.950608199	6.355287702	3.616499319	0.404679503
A_23_P411157	NM_005430	WNT1	6.083276228	9.687337999	6.557943618	6.396839562	3.604061771	-0.161104056
A_23_P122863	NM_001001555	GRB10	7.659615635	11.26195348	7.28169037	8.553797103	3.602337848	1.272106732
A_33_P3404052	NM_014350	TNFAIP8	9.954049597	13.55138774	9.522058385	11.69250257	3.597338146	2.170444188
A_24_P339944	NM_002608	PDGFB	7.895924658	11.49141849	6.820886693	9.358158473	3.595493835	2.53727178
A_23_P65651	NM_004184	WARS	11.29247948	14.86653152	13.09015362	13.82666377	3.574052035	0.736510143
A_23_P210482	NM_000022	ADA	10.78757164	14.35714033	11.52480743	13.01326767	3.569568685	1.48846024
A_33_P3272948	NM_001145536	C17orf107	7.336634245	10.8995073	7.348406781	9.964462955	3.562873055	2.616056173
A_23_P134085	NM_173515	CNKSR3	6.367116198	9.918568263	8.090269587	9.625093075	3.551452065	1.534823488
A_23_P37983	NM_005947	MT1B	10.92193799	14.47243695	15.58135283	14.2906686	3.550498958	-1.29068423
A_33_P3415032	NM_002232	KCNA3	6.264982884	9.795710181	6.03816356	8.38592616	3.530727296	2.347762599
A_23_P64617	NM_012193	FZD4	5.521024995	9.051361013	6.488117647	6.543947185	3.530336017	0.055829538
A_24_P77082	NM_003679	KMO	10.00065459	13.52548887	10.07912453	13.170507	3.524834281	3.091382468
A_24_P335305	NM_006187	OAS3	8.441164508	11.96302322	7.013635153	9.475914495	3.521858712	2.462279342
A_23_P435477	NM_001077263	TMPRSS13	6.353561202	9.874650525	7.213719394	7.550342394	3.521089323	0.336623
A_23_P64721	NM_006018	HCAR3	11.63601761	15.13430751	9.717987967	12.79762301	3.498289895	3.079635039
A_23_P77859	NM_203411	TMEM88	7.076026334	10.57071334	7.091728704	8.839878494	3.494687006	1.748149789
A_23_P156788	NM_003764	STX11	8.950024581	12.44132458	10.44016623	11.84414447	3.491299996	1.403978237
A_19_P00317053		XLOC_005810	5.374702855	8.860445139	5.497979721	8.095305371	3.485742284	2.59732565
A_24_P411899	NM_183419	RNF19A	9.44851922	12.92143299	9.615355606	11.94180891	3.47291377	2.326453301
A_23_P206724	NM_175617	MT1E	10.03238714	13.50119246	15.09305043	13.34604462	3.468805324	-1.747005807

A_24_P215765	NM_024490	ATP10A	7.703454206	11.1644834	6.602884143	6.499170501	3.461029194	-0.103713642
A_23_P308603	NM_005417	SRC	9.339246508	12.77787816	10.78600743	11.68557731	3.438631656	0.89956988
A_32_P120895	NM_153374	LYSMD2	9.80538692	13.23833042	9.662551792	11.54852542	3.432943504	1.885973625
A_23_P123853	NM_006274	CCL19	9.195869551	12.62341554	6.954612118	7.972064885	3.427545985	1.017452767
A_33_P3411628	NM_000077	CDKN2A	5.857009652	9.281145063	6.342388278	7.671780447	3.424135411	1.329392169
A_24_P30194	NM_012420	IFIT5	8.969050796	12.38624214	7.913304559	10.55072946	3.417191348	2.637424905
A_32_P47107	XR_108889	LOC100129104	8.888538654	12.30353174	8.723547208	11.70072539	3.414993083	2.977178179
A_23_P29953	NM_172175	IL15	9.472078806	12.86329938	8.802776176	9.926712425	3.391220574	1.123936248
A_33_P3350056	NM_005952	MT1X	10.91514767	14.30308183	15.93539333	14.31282916	3.387934165	-1.622564177
A_33_P3315314	NM_005951	MT1H	10.68586852	14.05286946	15.57951953	13.91118616	3.367000936	-1.668333373
A_23_P128974	NM_006399	BATF	11.71380574	15.07669271	10.27435968	11.36305035	3.362886966	1.08869067
A_33_P3400374	NM_001037335	PRIC285	11.30993789	14.67026937	9.121352206	12.35006991	3.36033148	3.228717707
A_23_P170857	NM_002182	IL1RAP	10.03747692	13.37665807	8.717938914	9.580950242	3.339181152	0.863011328
A_23_P69573	NM_000856	GUCY1A3	6.045198036	9.36822652	6.197535796	8.192066699	3.323028485	1.994530903
A_23_P148047	NM_000958	PTGER4	11.82931755	15.13727287	9.427406176	11.89539471	3.307955318	2.467988534
A_33_P3390950		LOC100128338	8.570264944	11.8684992	8.011463063	10.24909693	3.298234259	2.237633864
A_23_P50349	NM_004240	TRIP10	8.317622673	11.61486191	9.856393631	11.16631205	3.297239237	1.309918419
A_23_P112241	NM_012266	DNAJB5	10.11099446	13.40145821	11.18413457	13.39102156	3.290463754	2.206886993
A_33_P3268555	NM_001005176	SP140	7.574425007	10.85427696	7.756609823	8.389321777	3.27985195	0.632711954
A_33_P3298139	NM_001012288	CRLF2	6.179930135	9.457885846	6.139322249	6.346786238	3.277955712	0.207463989
A_24_P208567	NM_003855	IL18R1	6.038170815	9.303771864	5.425346325	5.97665418	3.265601048	0.551307855
A_23_P18598	NM_018323	PI4K2B	8.867958946	12.13089977	8.589161875	11.22511787	3.262940828	2.635955991
A_23_P56938	NM_002908	REL	9.746005509	12.99995435	9.481236207	11.94612539	3.253948838	2.464889186
A_23_P256948	NM_005098	MSC	7.431061214	10.67344697	8.108513879	9.921277025	3.242385752	1.812763146
A_23_P144916	NM_005110	GFPT2	5.19066558	8.429271377	5.432900421	6.384716239	3.238605797	0.951815818
A_23_P158880	NM_181900	STARD5	7.886311655	11.12449157	8.393449692	9.089722774	3.238179911	0.696273082
A_23_P398566	NM_173200	NR4A3	12.72573192	15.9636902	10.53301902	14.17293196	3.237958281	3.639912943
A_33_P3230269	NM_198182	GRHL1	6.281029707	9.515377503	5.58539136	6.307069916	3.234347796	0.721678556
A_33_P3264846	NM_152703	SAMD9L	10.01546965	13.24424381	7.310535029	10.00304028	3.22877416	2.692505247
A_23_P41470	NM_017631	DDX60	8.168114428	11.39372377	7.643544102	10.79357668	3.225609339	3.150032582
A_24_P942630	NM_001080424	KDM6B	5.505232158	8.723802646	6.263537686	7.688682649	3.218570488	1.425144963
A_33_P3225522	NM_001032731	OAS2	9.189680772	12.40256459	8.212328443	11.32857115	3.212883818	3.116242704
A_23_P420196	NM_003745	SOCS1	10.07308575	13.28370369	9.450716919	11.02246408	3.210617944	1.571747164
A_23_P41765	NM_002198	IRF1	8.554955171	11.75732525	8.609522281	9.982788985	3.202370082	1.373266704
A_23_P38346	NM_024119	DHX58	11.16770924	14.36290356	10.6508098	11.78401143	3.195194322	1.133201633
A_33_P3254320	NM_005490	SH2D3A	6.226640956	9.420198181	7.173520767	8.981922228	3.193557225	1.808401461
A_33_P3319870	NM_001191323	GREM1	5.365637751	8.54940601	6.075688464	9.529390721	3.183768259	3.453702257
A_33_P3299416	NM_181711	GRASP	9.731184288	12.91450532	9.559878828	9.987846925	3.183321032	0.427968097
A_24_P11506	NM_001032998	KYNU	10.27805654	13.4597099	11.05978824	13.47218935	3.181653352	2.41240111
A_23_P202156	NM_001077493	NFKB2	8.145293209	11.31549241	8.188597561	10.76342801	3.170199204	2.574830445
A_23_P168951	NM_014943	ZHX2	7.405352335	10.57342541	6.72127977	8.630742851	3.168073079	1.909463082
A_33_P3265504	NM_001099692	EIF5AL1	8.584725137	11.7420131	7.787709767	11.09382527	3.157287963	3.306115503
A_23_P250358	NM_017912	HERC6	5.602759792	8.755809268	5.577453665	7.062925651	3.153049477	1.485471986
A_24_P161018	NM_017554	PARP14	9.978017869	13.12314512	9.21340868	11.67689921	3.145127248	2.463490527
A_24_P239606	NM_015675	GADD45B	11.72419419	14.868005	11.66545891	12.44692474	3.14381081	0.781465823
A_24_P97405	NM_003965	CCRL2	7.150173434	10.29349648	8.249594751	10.86043117	3.143323041	2.610836422
A_23_P23266		BLZF1	8.577118025	11.71564011	7.599169835	8.922495404	3.138522082	1.323325569
A_23_P13094	NM_002425	MMP10	5.765055559	8.90309309	5.457015288	5.781896876	3.138037531	0.324881588
A_24_P317907	NM_001034954	SORBS1	5.844582097	8.979317355	5.799184025	7.431570052	3.134735258	1.632386027
A_23_P17053	NM_019618	IL36G	5.507504539	8.641198359	6.756300477	8.771975268	3.13369382	2.015674791
A_23_P323761	NM_025228	TRAF3IP3	9.568570165	12.68902704	10.11675598	10.52910105	3.120456878	0.412345074
A_23_P315122	NM_004097	EMX1	10.24510912	13.36312704	9.874168715	12.90589902	3.118017919	3.031730302
A_23_P14124	NM_206827	RASL11A	6.049292952	9.161824734	6.196006613	6.688450172	3.112531782	0.492443559
A_23_P104318	NM_019058	DDIT4	8.507521043	11.61965802	12.56550901	14.29728971	3.112136977	1.731780697
A_24_P406060	NM_182757	RNF144B	10.09287095	13.19847847	8.503754274	12.06666728	3.105607521	3.562913009
A_33_P3373364	NM_013943	CLIC4	10.76556792	13.86857932	10.28391809	12.65441496	3.103011404	2.370496873
A_23_P352870	NM_002856	PVRL2	8.091601909	11.19422338	8.310474394	9.271233589	3.102621471	0.960759194
A_23_P21560	NM_030797	FAM49A	10.3397853	13.4375267	11.85886128	11.00552281	3.097741397	-0.853338467
A_23_P110212	NM_001995	ACSL1	10.15883188	13.25394479	11.88175362	14.01910398	3.095112912	2.13735036
A_23_P345118	NM_002648	PIM1	9.68375564	12.77379023	11.74798201	12.62238159	3.09003459	0.874399579
A_23_P73097	NM_170587	RGS20	5.678761334	8.750283719	6.467343884	6.869092831	3.071522385	0.401748947
A_23_P252106	NM_003821	RIPK2	11.00979034	14.07685619	11.9670524	14.06100105	3.067065846	2.093948653
A_23_P47614	NM_003311	PHLDA2	9.955336097	13.02205377	8.33675574	10.74379012	3.066717673	2.40703438
A_23_P56213	NM_020895	GRAMD1A	6.501872866	9.562614719	7.617594516	10.03073591	3.060741854	2.413141397
A_23_P210886	NM_138578	BCL2L1	7.780802229	10.84042438	8.099580016	8.572131718	3.059622147	0.472551703
A_24_P411121	NM_148901	TNFRSF18	6.621515691	9.677903031	6.068471353	6.592066784	3.05638734	0.523595431
A_33_P3252196	NM_004456	EZH2	8.858182751	11.90690773	8.682723513	11.11313146	3.048724982	2.430407951
A_23_P12343	NM_000849	GSTM3	9.222322069	12.27047916	8.163126909	9.267317395	3.048157088	1.104190486
A_23_P329924	NM_177551	HCAR2	7.033386535	10.07761097	6.593950658	7.232872748	3.044224434	0.63892209
A_24_P941167	NM_030641	APOL6	7.31486269	10.35587499	7.717613255	8.372228196	3.0410123	0.654614941
A_24_P943997	NM_178815	ARL5B	6.351786638	9.390602096	6.338368736	7.832609234	3.038815458	1.494240498
A_33_P3360072	NM_001161429	RANBP3L	5.252491706	8.28964849	5.544834836	7.024442201	3.037156784	1.479607365
A_23_P319423	NM_003740	KCNK5	7.411195959	10.43758117	9.553823506	10.34916993	3.026385208	0.795346428
A_32_P225854	NM_181784	SPRED2	6.831193018	9.857124189	7.903921424	8.69117654	3.02593117	0.787255116
A_33_P3297415	NM_201264	NRP2	6.653262062	9.672649144	8.908677085	9.775665334	3.019387083	0.866988249
A_33_P3338121	NM_001017402	LAMB3	8.623052723	11.64175606	9.971801062	12.00300939	3.018703337	2.031208324
A_33_P3345534	NM_000526	KRT14	7.206552838	10.21376831	5.829998247	6.038632183	3.007215469	0.208633936
A_33_P3384835	XR_110561	LOC100505719	8.130908677	11.13341638	7.226968005	7.378229498	3.002507703	0.151261493
A_23_P99163	NM_018370	DRAM1	11.33208346	14.32093575	12.33916277	13.81691048	2.988852295	1.477747713
A_23_P6151	NM_002657	PLAGL2	8.78600525	11.77435882	8.840942343	10.72790414	2.988353573	1.886961797
A_32_P26969	NM_207332	ERICH1	10.14693469	13.12458545	9.037347157	9.567521796	2.977650755	0.530174639
A_23_P5903	NM_016354	SLCO4A1	7.678957063	10.64607635	10.72826576	12.05985425	2.96711929	1.331588493
A_23_P128215	NM_003877	SOCS2	9.008786053	11.97311361	7.047593305	6.913370063	2.964327554	-0.134223242
A_23_P390097	NM_152574	TTC39B	8.361561077	11.32345997	8.085307429	9.048380131	2.961898896	0.963072702
A_33_P3258392	NM_001955	EDN1	5.82105855	8.772440431	8.294968085	12.63356912	2.951381881	4.338601039
A_23_P33326	NM_000679	ADRA1B	5.579176635	8.515649118	5.75058745	6.264209525	2.936472484	0.513622075
A_24_P237613	NM_182488	USP12	6.886092881	9.820676375	6.978869549	8.629754575	2.934583494	1.650885026
A_33_P3219090	NM_005542	INSIG1	12.46911417	15.40344853	13.39813974	14.58008813	2.934334365	1.181948393
A_23_P30069	NM_001012967	DDX60L	8.961663841	11.88742476	9.489696446	11.30070002	2.925760923	1.811003571
A_23_P80068	NM_006806	BTG3	10.17894336	13.08818385	11.77998308	13.47582057	2.90924049	1.695837493
A_23_P207367	NM_003152	STAT5A	10.39447307	13.30224292	9.95175506	11.25755594	2.907769849	1.305800883
A_23_P48414	NM_003914	CCNA1	6.744345017	9.645614785	5.987851346	8.072306763	2.901269768	2.084455417
A_23_P386364	NM_144657	HDX	5.711114237	8.600250808	5.662575041	7.093488827	2.889136572	1.430913786
A_24_P207139	NM_033238	PML	7.706057951	10.59296998	7.888103063	8.61353532	2.886912026	0.725432257
A_24_P335620	NM_003486	SLC7A5	7.725456282	10.60159224	7.933943143	10.38199875	2.876135961	2.448055607
A_23_P166797	NM_022147	RTP4	10.13289618	13.00811026	8.809707222	10.18316885	2.875214076	1.373461632
A_24_P263910		C14orf182	6.095351706	8.966182196	6.280449596	8.947535122	2.87083049	2.667085526
A_32_P96000	NM_001004341	ETV3L	5.305383779	8.175377851	5.35613244	5.631512812	2.869994072	0.275380371
A_33_P3306024	NR_002825	SIGLEC16	9.335847084	12.20473324	9.53662307	9.978188832	2.868886159	0.441565762
A_33_P3224803	NM_000265	NCF1	8.269672918	11.13632093	9.317308779	9.571216186	2.866648008	0.253907408
A_33_P3259393	NM_178232	HAPLN3	9.407493115	12.27071111	8.150845693	8.77982795	2.863217998	0.628982257

A_33_P3734384		LOC285957	6.567653841	9.4228954	6.328607587	8.192906014	2.855241559	1.864298427
A_23_P29773	NM_014398	LAMP3	12.73379229	15.5867191	6.177028956	10.00656106	2.852926817	3.829532105
A_33_P3219591	NM_020954	RNF213	8.442332356	11.29439082	8.097538217	8.708330261	2.852058467	0.610792044
A_23_P119337	NM_012068	ATF5	11.15959488	13.98683654	9.071006398	10.3278403	2.827241659	1.256833898
A_23_P153320	NM_000201	ICAM1	11.31779189	14.14068294	11.18171044	13.63763318	2.822891051	2.45592274
A_23_P316381	NM_003501	ACOX3	9.16841232	11.9910875	9.551201577	11.19613191	2.822675177	1.644930333
A_23_P18078	NM_002888	RARRES1	7.292560595	10.11464357	6.038071234	8.784211922	2.822082975	2.746140688
A_33_P3249185		SAR1A	9.538209809	12.35927442	10.48915632	10.83122879	2.821064611	0.342072466
A_32_P83784	NM_015230	ARAP2	10.10406034	12.91949053	9.253310824	8.971473388	2.815430194	-0.281837436
A_23_P344568	NM_145019	FAM124A	5.587150862	8.400087885	6.414038021	8.649228467	2.812937023	2.235190446
A_23_P133095	NM_014247	RAPGEF2	10.87264565	13.68349642	10.4897593	12.4833319	2.81085077	1.9935726
A_33_P3343120	NM_002163	IRF8	11.20558715	14.01557332	13.00554908	13.69810544	2.809986166	0.692556357
A_23_P124642	NM_005739	RASGRP1	6.829314399	9.639110171	6.336135978	9.507479886	2.809795772	3.171343908
A_33_P3380056	NM_001198807	C9orf30	6.006294141	8.810428203	6.124515242	9.35036268	2.804134063	3.225847439
A_23_P51646	NM_004073	PLK3	10.99026678	13.77000169	13.05070674	14.87200667	2.779734911	1.821299933
A_33_P3397418	NM_020119	ZC3HAV1	11.47564199	14.25494513	11.86049705	13.84907686	2.779303138	1.988579807
A_32_P56249	NR_038996	LOC100131733	8.097585319	10.86453064	7.848299388	9.327644559	2.766945318	1.479345171
A_23_P359043	NM_001004065	AKAP2	6.044918714	8.810313998	5.908650185	8.641213535	2.765395284	2.73256335
A_23_P12572	NM_033338	CASP7	9.999689876	12.76434964	10.02938767	11.42793978	2.764659768	1.398552113
A_23_P259594	NM_016377	AKAP7	8.201604111	10.95741694	8.641623631	9.38142712	2.755812829	0.739803489
A_33_P3370404	NM_015368	PANX1	9.286564469	12.03722632	8.458780813	10.17279841	2.750661851	1.714017594
A_33_P3356210	NM_001202439	B7H6	8.315324028	11.06477425	8.089203024	9.756061651	2.749450222	1.666858627
A_24_P278126	NM_002485	NBN	8.317155984	11.06452311	8.143578524	11.18480619	2.74736713	3.041227662
A_23_P132057	NR_024027	LINC00158	5.667515284	8.41197587	5.567403447	6.63572047	2.744460586	1.068317023
A_33_P3372257	NM_004272	HOMER1	6.899804309	9.644115416	6.951802405	8.467572918	2.744311107	1.515770513
A_23_P45851	NM_033055	HIAT1	10.91412071	13.65247864	10.79613675	11.5554857	2.738357929	0.759348943
A_23_P257043	NM_005261	GEM	7.866706895	10.60165787	10.33440862	12.63177558	2.734950974	2.29736696
A_23_P126735	NM_000572	IL10	8.688046707	11.41571222	7.269943955	10.85162156	2.72766551	3.581677601
A_32_P179740	NM_020725	ATXN7L1	7.984900165	10.71124646	7.40160218	8.345705541	2.726346291	0.944103361
A_33_P3878772	NM_004972	JAK2	9.977969641	12.70103679	9.487239046	10.34266271	2.723067145	0.855423667
A_33_P3296940	NM_022763	FNDC3B	8.586559185	11.30334405	8.144422826	10.46924688	2.716784868	2.32482405
A_24_P30923	NM_003498	SNN	9.917154829	12.63031	10.62066232	11.92076838	2.713155174	1.300106058
A_33_P3393766	NM_001130677	C17orf96	12.7185064	15.41749889	12.71671746	15.8133028	2.698992493	3.096585333
A_23_P19624	NM_001718	BMP6	6.346782754	9.043685552	9.777521108	10.56205102	2.696902799	0.784529909
A_33_P3641714	NM_018381	C19orf66	10.74581772	13.4347662	10.89060257	13.22161408	2.688948483	2.331011517
A_23_P200096	NM_025106	SPSB1	7.748754753	10.43717541	9.177563546	9.707621496	2.688420657	0.530057949
A_23_P156683	NM_000595	LTA	6.034318534	8.719894077	5.942154574	6.897271736	2.685575543	0.955117162
A_32_P181297	NR_002330	ST7-AS1	6.579691491	9.262867095	6.240726638	7.285182122	2.683175604	1.044455484
A_33_P3343428	NM_147686	TRAF3IP2	7.43616814	10.11576012	10.31206636	12.41490097	2.679591984	2.102834613
A_32_P75581	NM_152414	BHLHE22	5.458754879	8.126786628	5.134164463	5.795963815	2.668031749	0.661799352
A_33_P3387796	NM_015157	PHLDB1	6.617007038	9.284923189	6.82035478	7.915817198	2.667916151	1.095462418
A_33_P3222451		AKT1S1	6.207221015	8.869287603	5.864341454	6.773515424	2.662066587	0.909173969
A_23_P321388	NM_153341	RNF19B	8.114199123	10.77369903	8.003020103	10.11304254	2.659499904	2.110022441
A_23_P79094	NM_006865	LILRA3	9.24599155	11.90503085	12.16585093	13.20464413	2.659039304	1.038793203
A_24_P11061	NM_153478	CSAG1	5.762839327	8.420199589	6.040943309	6.046043482	2.657360262	0.005100172
A_32_P41496	NR_027761	LOC100132831	8.400423687	11.05400928	8.119170724	10.08225212	2.653585589	1.963081391
A_24_P126628	NM_015257	TMEM194A	9.716736168	12.35769718	9.119509805	11.23100694	2.640961009	2.111497132
A_24_P125839	NM_017447	C21orf91	7.288403732	9.921192704	6.672607334	7.173228367	2.632788971	0.500621033
A_23_P30435	NM_006058	TNIP1	12.6088192	15.24099816	12.66296835	15.21326976	2.632178964	2.55030141
A_23_P23221	NM_001924	GADD45A	9.693825553	12.32166917	9.610945763	10.9314919	2.627843614	1.32054614
A_32_P76035	NM_033109	PNPT1	6.40077166	9.026609681	6.571048044	7.592988113	2.62583802	1.021940069
A_23_P66432	NM_032646	TTYH2	6.58838614	9.213718122	6.958820827	7.954856319	2.625331982	0.996035492
A_24_P314179	NM_005239	ETS2	7.288110018	9.911635918	5.946534973	7.879990231	2.6235259	1.933455258
A_23_P110712	NM_004417	DUSP1	11.05354835	13.67120569	9.774070632	11.92396016	2.617657338	2.149889531
A_23_P67980	NM_003709	KLF7	6.705145124	9.321668324	8.070520149	7.935086821	2.6165232	-0.135433329
A_23_P142750	NM_002759	EIF2AK2	10.50593843	13.11652226	9.404825021	11.14302962	2.61058383	1.738204602
A_23_P256716	NM_003601	SMARCA5	8.847889178	11.45187094	8.985176613	10.67120125	2.603981758	1.686024633
A_23_P6963	NM_003341	UBE2E1	8.561053137	11.16459498	8.514309703	9.364602473	2.60354184	0.850292769
A_23_P137423	NM_052868	IGSF8	7.869059467	10.46819024	9.947630085	8.626556776	2.599130775	-1.321073309
A_33_P3398912	NM_017585	SLC2A6	12.14608654	14.738028	12.32935032	14.8227066	2.591941455	2.493356273
A_23_P310	NM_023009	MARCKSL1	8.64480938	11.23495721	6.798510008	8.522414084	2.59014783	1.723904076
A_32_P26376	NM_152305	POGLUT1	8.859504125	11.44933553	7.940942242	7.907320834	2.589831408	-0.033621408
A_23_P120002	NM_004510	SP110	10.255776	12.84538589	9.592860648	11.21496118	2.589609892	1.622100536
A_33_P3245348	NM_033060	KRTAP4-1	6.340693128	8.927027994	6.165146178	8.639795542	2.586334865	2.474649364
A_23_P16469	NM_001005377	PLAUR	11.58372489	14.16897355	12.9167992	14.28730329	2.585248657	1.370504093
A_24_P557479	NM_017523	XAF1	9.576888572	12.16126903	7.822420105	9.765397323	2.584380458	1.942977217
A_32_P95067	NM_001005353	AK4	7.374146073	9.956434229	9.47472735	10.03254765	2.582288156	0.5578203
A_23_P112774	NM_032611	PTP4A3	6.589885186	9.172168212	6.78231779	6.962847326	2.582283026	0.180529536
A_33_P3331641	NR_024421	ZNF503-AS2	5.812899907	8.383840036	5.872166883	6.155741198	2.57094013	0.283574315
A_24_P273143	NR_024204	LINC00152	10.79547063	13.3623064	12.24697238	14.19084559	2.566835773	1.94387321
A_33_P3332156	NM_001003665	C1orf95	6.313455248	8.876743973	6.131976543	6.13225215	2.563288725	0.000275607
A_23_P135271	NM_001497	B4GALT1	8.277433691	10.83891799	9.102475203	10.11041036	2.561484299	1.007935155
A_24_P378019	NM_004031	IRF7	12.36720183	14.92688621	11.5882037	14.23443469	2.559684377	2.646230987
A_24_P23034	NM_021035	ZNFX1	8.892917073	11.45220127	9.031186017	10.2742838	2.559284194	1.243097787
A_23_P258418	NM_024309	TNIP2	8.374787547	10.93120597	8.134254367	10.00990558	2.55641842	1.875651215
A_24_P229531	NM_001031716	OBFC2A	9.150379595	11.70542567	7.946312941	9.35066536	2.555046078	1.404352419
A_24_P294408	NM_015900	PLA1A	5.607334356	8.15989326	5.546512561	5.99234234	2.552558904	0.445829778
A_33_P3228460	NM_001136007	FXYD3	8.140744496	10.69100148	6.572169905	9.511275784	2.550256984	2.939105879
A_23_P113701	NM_002607	PDGFA	7.132500592	9.680560161	7.840691305	8.463848003	2.548059569	0.623156697
A_23_P421423	NM_006291	TNFAIP2	14.06449889	16.60796103	14.05792773	16.78642184	2.543462135	2.728494113
A_32_P53524	NM_004822	NTN1	6.081867747	8.621811503	5.473851801	6.267880575	2.539943756	0.794028774
A_24_P12435	NM_181782	NCOA7	11.07254294	13.61201929	9.993813904	11.89724303	2.539476349	1.903429126
A_33_P3341499	NM_003392	WNT5A	8.461036854	10.99423593	8.938236486	10.5520348	2.533199076	1.613798314
A_23_P94703	NM_014506	TOR1B	9.582588358	12.11553983	9.046035126	10.16548055	2.532951475	1.119445423
A_23_P19291	NM_001069	TUBB2A	12.1436696	14.66073123	9.368413022	12.29919717	2.517061633	2.930784145
A_23_P123672	NM_014290	TDRD7	10.92263114	13.43797867	10.10393188	11.66792516	2.51534753	1.563993285
A_23_P90172	NM_014330	PPP1R15A	9.660145066	12.17120042	10.96984308	11.5321457	2.511055358	0.562302617
A_23_P112159	NM_012154	EIF2C2	9.116653428	11.62724083	10.31849283	10.91586383	2.510587399	0.597370997
A_23_P32414	NM_016542	MST4	9.673863451	12.18382214	10.62937637	12.90169679	2.509958689	2.272320423
A_23_P161152	NM_014317	PDSS1	9.287674354	11.79671962	9.85617503	12.45665105	2.509045262	2.600476024
A_23_P25150	NM_006897	HOXC9	5.353264363	7.859840249	5.31912997	5.430160233	2.506575885	0.111030263
A_23_P218626	NM_080741	NEU4	5.317667355	7.821564896	5.641135457	6.167373821	2.503897541	0.526238364
A_33_P3371089	NM_019072	SGTB	10.69679066	13.19777423	9.705584944	11.60629553	2.500983567	1.900710586
A_23_P71530	NM_002546	TNFRSF11B	7.581980324	10.08262147	5.629701372	5.892845454	2.500641148	0.263144082
A_23_P36397	NM_000785	CYP27B1	7.415604035	9.905588622	10.3406765	13.60940261	2.489984588	3.268726102
A_33_P3364646	NM_152545	RASGEF1B	8.208830938	10.69838512	9.749878721	9.594689495	2.489554182	-0.155189226
A_23_P54840	NM_005946	MT1A	12.29628986	14.78090315	16.17104156	14.83672013	2.484613288	-1.33432143
A_23_P51487	NM_018284	GBP3	8.975877357	11.46046942	7.634152072	10.45146954	2.484592067	2.817317463
A_23_P85693	NM_004120	GBP2	10.45509017	12.9378724	11.49019178	12.1804216	2.48278223	0.690229817
A_23_P62901	NM_006763	BTG2	12.73443599	15.21519258	12.31821691	14.47045212	2.480756595	2.152235207
A_33_P3241541		FSD1L	5.814123193	8.293044945	5.830018252	6.681694998	2.478921752	0.851676747

A_23_P4714	NM_006533	MIA	7.447827534	9.926257651	6.316944865	6.182476037	2.478430117	-0.134468828
A_23_P18824	NM_006999	PAPD7	9.385716117	11.86399582	8.977867128	10.20723955	2.478279699	1.229372426
A_24_P60845	NM_000665	ACHE	7.180950705	9.656259154	7.072871754	9.168610394	2.475308448	2.095738639
A_24_P241183	NM_013269	CLEC2D	6.525753098	8.998251263	6.242442285	6.474877526	2.472498165	0.232435241
A_23_P127565	NM_178834	LAYN	7.747641791	10.21775355	7.115020438	7.180344277	2.470111755	0.065323838
A_24_P158089	NM_000602	SERPINE1	5.710352796	8.17941519	8.339577413	10.51123483	2.469062394	2.171657417
A_33_P3333317	NM_001008211	OPTN	8.021402184	10.48898049	7.143752158	8.904083867	2.467578308	1.76033171
A_33_P3216714	NM_014787	DNAJC6	5.556829881	8.021195452	6.350204481	7.382333043	2.464365571	1.032128562
A_23_P116653	NM_138722	BCL2L14	5.505868775	7.958257427	5.472232259	5.802753533	2.452388652	0.330521274
A_24_P379104	NM_006875	PIM2	9.806887663	12.25545866	9.872947903	11.47455839	2.448571001	1.601610487
A_32_P60459	NM_001145373	OTUD1	10.30525204	12.74600437	9.896016228	11.01261057	2.440752331	1.116594338
A_33_P3336925	NM_001011516	PDLIM5	8.968815665	11.40754428	9.164348016	10.94676855	2.438728615	1.782420531
A_23_P214950	NM_022121	PERP	5.730442669	8.161447855	5.674635954	7.286008414	2.431005186	1.61137246
A_23_P22350	NM_023927	GRAMD3	6.415229956	8.833223762	6.104539225	8.511516452	2.417993806	2.406977227
A_23_P352266	NM_000633	BCL2	7.144491383	9.562437539	7.42489521	9.7530918	2.417946156	2.32819659
A_23_P42718	NM_004289	NFE2L3	9.829525188	12.24452869	9.376000684	10.13253114	2.415003499	0.756530456
A_33_P3387155	NR_002814	LOC374443	7.828013157	10.23554586	6.790999247	7.731953839	2.4075327	0.940954591
A_23_P88069	NM_005780	LHFP	6.988506641	9.389401579	7.243470904	10.84067389	2.400894939	3.597202986
A_24_P351283	NM_018000	MREG	8.468442911	10.86729641	8.944623676	11.1682467	2.398853499	2.223623024
A_24_P576174	NM_018403	DCP1A	9.85456111	12.25302099	9.799719038	10.98025593	2.398459884	1.180536895
A_33_P3289356	NM_001779	CD58	10.85582697	13.25408893	10.3799612	11.70208405	2.39826196	1.322122843
A_23_P94216	NM_152271	LONRF1	7.528050367	9.90933261	7.626693881	9.734227074	2.381282242	2.107533193
A_24_P175187	NM_017654	SAMD9	6.908025165	9.288249771	6.432631229	7.868573918	2.380224606	1.43594269
A_32_P175934	NM_001778	CD48	8.472572422	10.85241203	10.81368298	12.75797839	2.379839608	1.944295407
A_33_P3286536	NM_002033	FUT4	9.707295657	12.08688408	9.092910393	11.38906501	2.37958842	2.296154614
A_23_P160720	NM_018664	BATF3	11.3132818	13.69257692	9.222056496	10.87989912	2.379295118	1.657842628
A_23_P351275	NM_181597	UPP1	10.41631844	12.7896981	11.48311024	13.24831481	2.373379668	1.76520457
A_23_P370682	NM_138456	BATF2	8.572847573	10.94371328	8.405377979	10.62713454	2.37086571	2.221756558
A_33_P3390521	NM_023079	UBE2Z	7.133140925	9.502816852	7.633513619	8.229995881	2.369675927	0.596482262
A_23_P364465	NM_022913	GPBP1	11.33226214	13.69426673	11.42639368	11.64793585	2.362004583	0.221542177
A_24_P53353	NM_001025300	RAB12	7.486093824	9.837871271	7.482571309	8.263987163	2.351777447	0.781415854
A_24_P322353	NM_024430	PSTPIP2	9.096929549	11.44619806	8.385928956	11.04179306	2.349268514	2.6558641
A_23_P26583	NM_032206	NLRC5	6.436135388	8.783757601	6.778292156	7.060283872	2.347622213	0.281991716
A_33_P3324004	NM_032965	CCL15	7.120328947	9.463460227	7.139609875	8.588372122	2.34313128	1.448762248
A_32_P183904	NM_138356	SHF	6.916490994	9.257336519	6.999243794	6.676591585	2.340845526	-0.322652209
A_24_P406986	NM_199329	SLC43A3	9.085630445	11.42368139	11.10225559	10.90754558	2.338050942	-0.19471001
A_33_P3879161	NM_152309	PIK3AP1	7.843390775	10.18120114	10.29550805	10.2228368	2.337810368	-0.072671244
A_23_P69109	NM_021105	PLSCR1	9.444977967	11.77953034	9.269542653	10.50365431	2.334552373	1.234111653
A_23_P322704	NM_001079519	FAM177A1	10.42737897	12.76070588	9.50011886	10.25980605	2.333326905	0.759687193
A_23_P94422	NM_014791	MELK	6.554648351	8.887712331	5.645848262	7.341832494	2.33306398	1.695984232
A_23_P69383	NM_031458	PARP9	9.7786706	12.10773513	8.446288605	11.07841223	2.32906453	2.632123625
A_33_P3342126	NM_020226	PRDM8	7.12352537	9.451346413	5.593888717	6.539642998	2.327821043	0.945754281
A_23_P152782	NM_005533	IFI35	10.01145661	12.33558197	9.489213553	10.29461433	2.324125363	0.805400773
A_23_P309619	NM_001145206	KIAA1671	7.604777714	9.920248673	5.713503692	5.65701961	2.315470959	-0.056484082
A_23_P91943	NM_000882	IL12A	5.112085045	7.425713593	5.363142405	6.228330758	2.313628548	0.865188353
A_23_P41344	NM_001432	EREG	6.385160114	8.695253373	6.069639582	9.013248583	2.310093259	2.943609001
A_23_P30547	NM_005565	LCP2	12.14902276	14.45568265	11.83238613	13.47347781	2.306659891	1.641091677
A_23_P59005	NM_000593	TAP1	13.1910748	15.49030032	13.05915183	14.20771078	2.299225522	1.148558947
A_24_P257108	NM_133484	TANK	8.199860266	10.49871247	8.611438461	9.048268769	2.298852208	0.436830308
A_24_P104119	NM_019034	RHOF	7.529281412	9.825708702	8.088067788	9.138337459	2.29642729	1.050269671
A_23_P134935	NM_001394	DUSP4	8.470488316	10.76268016	8.276479461	8.639863305	2.292191847	0.363383844
A_24_P941912	NM_138287	DTX3L	8.168224328	10.4591044	7.68472189	9.061353398	2.290880077	1.376631508
A_24_P396375	NM_001113347	ECE1	6.231850049	8.522077829	7.724624154	9.412514175	2.29022778	1.687890021
A_23_P64828	NM_002534	OAS1	9.364136192	11.65361844	10.04931009	10.13847305	2.289482252	0.089162959
A_23_P3681	NM_018092	NETO2	9.806304028	12.09336905	8.834585135	10.30324884	2.287065022	1.468663708
A_23_P148785	NM_199344	SFT2D2	9.579874293	11.86533947	8.762747834	9.837619006	2.285465173	1.074871172
A_23_P376096	NM_182919	TICAM1	7.331022734	9.615768965	7.980071886	8.799751627	2.284746232	0.819679741
A_23_P1602	NM_006779	CDC42EP2	6.570598119	8.853930156	6.195303102	8.407930705	2.283332037	2.212627603
A_23_P435521	NM_145041	TMEM106A	8.235920672	10.51834114	7.092761096	8.713667278	2.282420465	1.620906183
A_24_P367602	NR_002834	DUSP5P	5.697732634	7.978433795	5.531482777	6.312100189	2.280701161	0.780617412
A_33_P3264790		GJD4	5.372980437	7.651797041	5.482451825	5.901968275	2.278816604	0.41951645
A_23_P70670	NM_004233	CD83	13.83020967	16.10761534	13.30228219	15.06692694	2.277405672	1.764644757
A_24_P261259	NM_004566	PFKFB3	9.710321596	11.9870262	10.12677993	10.90019246	2.2767046	0.773412525
A_32_P62963	NR_029392	KRT16P2	6.38366682	8.658484193	5.796676555	5.706413016	2.274817373	-0.090263539
A_33_P3246829	NM_173843	IL1RN	10.51919964	12.7874622	13.14480159	14.91699231	2.268262556	1.772190713
A_23_P104109	NM_012424	RPS6KC1	8.400025887	10.66763317	8.180626593	9.92815402	2.267607283	1.747527427
A_19_P00320881		XLOC_012852	5.574903181	7.841203544	5.466875314	5.730918287	2.266300362	0.264042973
A_24_P916378		HNRPLL	6.910237255	9.174573109	6.926088426	7.06314469	2.264335854	0.137056264
A_23_P41424	NM_022154	SLC39A8	11.07586262	13.31772352	10.51833575	13.53852702	2.241860903	3.02019127
A_24_P350759	NM_004171	SLC1A2	5.850178525	8.090512939	6.027015103	8.095077259	2.240334413	2.068062156
A_33_P3321303	NM_001145312	ETV3	5.951630769	8.185516828	5.80525666	6.261726764	2.233886059	0.456470104
A_33_P3308167	NR_003655	POLR2J4	5.880543607	8.113647874	5.954024203	7.381139222	2.233104267	1.427115018
A_24_P171268	NM_182663	RASSF5	10.46689924	12.69851791	10.29588614	10.89337679	2.23161867	0.597490647
A_23_P204801	NM_032148	SLC41A2	8.419384592	10.64959177	7.240054963	8.641551835	2.230207175	1.401496872
A_33_P3367361	NM_031453	FAM107B	9.397239711	11.62610177	10.48230487	11.11790996	2.228862056	0.635605089
A_23_P411851	NM_152592	C14orf49	5.888989688	8.114869398	6.285583394	6.481316743	2.22587971	0.195733349
A_23_P209678	NM_002664	PLEK	12.74399601	14.96852071	13.57158485	15.28195367	2.224524707	1.710368817
A_33_P3405424	NM_172374	IL4I1	14.5025045	16.72579441	14.0412164	15.57173709	2.223289909	1.53052069
A_24_P65616	NM_006505	PVR	7.874747337	10.09467838	7.649768292	8.840298716	2.219931044	1.190530424
A_23_P206077	NM_022767	AEN	6.838685666	9.054943263	7.172484777	7.784330373	2.216257596	0.611845596
A_33_P3713357	NM_001627	ALCAM	11.67938416	13.89461355	11.59724207	12.92495699	2.215229389	1.32771492
A_33_P3315268	NM_173352	KRT78	7.817796632	10.03285197	7.687584231	7.743430677	2.215055338	0.055846446
A_23_P319617	NM_019886	CHST7	8.918719374	11.13342159	10.01880333	12.65883696	2.21470222	2.640033628
A_33_P3243618	NM_003272	GPR137B	9.908219939	12.12202093	8.759381614	9.109984891	2.213800994	0.350603276
A_24_P296698	NM_145109	MAP2K3	13.26754865	15.47699688	13.0290872	14.95714076	2.209448225	1.92805356
A_33_P3383283	NM_032977	CASP10	9.178462953	11.38644458	10.26176892	10.35899833	2.207981627	0.097229416
A_24_P226278	NM_015288	PHF15	7.962805005	10.16361705	8.25589114	8.112119564	2.200812049	-0.143771576
A_33_P3263217	NM_022143	LRRC4	6.303974468	8.49965834	5.82826168	6.0717012	2.195683872	0.243439521
A_23_P61398	NM_001001852	PIM3	13.7341138	15.92738185	13.1839995	15.98651261	2.193268051	2.802513113
A_23_P120822	NM_016327	UPB1	6.48320311	8.667177479	6.46601963	7.977325077	2.183974369	1.511305447
A_24_P411186	NM_022893	BCL11A	7.284771401	9.464897259	7.290753144	8.692584121	2.180125858	1.401830976
A_23_P161338	NM_021129	PPA1	11.22402499	13.40159728	12.16993662	12.57102314	2.177572292	0.40108652
A_33_P3285470	NM_001702	BAI1	8.568838572	10.74478648	10.36021229	10.58792895	2.175947905	0.227716658
A_23_P134663	NM_024812	BAALC	5.236117503	7.40512768	5.586180488	7.336120348	2.169010177	1.749939859
A_23_P10858	NM_015114	ANKLE2	7.471356192	9.63928869	7.872998356	9.547519186	2.167932498	1.67452083
A_23_P343398	NM_001838	CCR7	12.70264466	14.86469176	7.821083787	9.90795663	2.162047095	2.086872844
A_33_P3292478	NM_004590	CCL16	11.1730507	13.33500654	10.54813711	12.2011997	2.161955839	1.653062596
A_32_P315395	NR_026914	MGC16275	6.563593255	8.720795682	6.312477008	7.199207474	2.157202427	0.886730466
A_23_P68922	NM_033386	MICALL1	6.926206844	9.081438364	7.33977275	8.74700951	2.15523152	1.40723676
A_32_P135348	NM_033394	TANC1	5.501827566	7.654456556	5.469290134	5.686660307	2.15262899	0.217370173
A_24_P169092	NM_032427	MAML2	6.514645455	8.661755305	7.136680579	7.781583572	2.147109851	0.644902992

A_23_P144337	NM_012118	CCRN4L	5.383382219	7.521231994	5.882233584	6.553720495	2.137849775	0.671486911
A_24_P374319	NM_021183	RAP2C	8.69083766	10.82440874	8.891968157	10.16491931	2.133571077	1.272951157
A_32_P1173	NM_138441	MB21D1	9.141798186	11.27241386	8.454687977	9.527840911	2.130615674	1.073152933
A_23_P342053	NM_032626	RBBP6	7.029299066	9.159221364	7.105122317	7.609582942	2.129922298	0.504460625
A_24_P89457	NM_078467	CDKN1A	9.325803415	11.45277824	10.54259382	10.3780715	2.126974821	-0.16452232
A_23_P309701	NM_002828	PTPN2	7.385011108	9.510210948	6.995338651	8.373982144	2.12519984	1.378643493
A_24_P365025	NM_003971	SPAG9	10.31773132	12.44277805	10.85296661	11.80091083	2.125046727	0.947944223
A_23_P19619	NM_002114	HIVEP1	7.228334343	9.353259271	7.458106514	10.27572774	2.124924927	2.81762123
A_23_P108751	NM_001039492	FHL2	7.877218693	10.00181014	6.054245731	6.791548766	2.124591443	0.737303035
A_33_P3411075	NM_003088	FSCN1	14.57192176	16.6861402	14.0498868	14.09874649	2.114218437	0.04885969
A_23_P66241	NM_176870	MT1M	6.307588521	8.420722855	11.09514634	7.974676439	2.113134334	-3.120469903
A_23_P215227	NM_005494	DNAJB6	11.59080892	13.70087868	12.02744673	12.58938532	2.110069764	0.561938587
A_23_P142205	NM_006866	LILRA2	7.947957353	10.05769761	10.78995181	10.76690966	2.109740257	-0.02304215
A_33_P3489737	NM_020726	NLN	9.213363433	11.31993874	8.143238328	9.784495501	2.106575307	1.641257174
A_33_P3367247	NM_001114395	CNTLN	7.045783993	9.151840603	6.527761593	7.846141959	2.10605661	1.318380366
A_23_P303455	NM_153832	GPR161	5.920082631	8.023146197	5.543517653	5.836639579	2.103063566	0.293121925
A_33_P3275707	NM_001039569	AP1S3	7.877894285	9.976302814	7.020687666	7.806777134	2.098408529	0.786089468
A_23_P127948	NM_001124	ADM	11.60363011	13.70138201	14.38015922	14.11608569	2.097751896	-0.264073523
A_24_P56240	NM_153634	CPNE8	8.099878176	10.19700839	7.479778738	8.129038056	2.097130216	0.649259318
A_33_P3361891	NM_001042575	TMPRSS7	5.710913303	7.806377043	6.115177189	7.310589242	2.095463739	1.195412053
A_33_P3402329	NR_024607	MGC16121	8.897657534	10.99307375	7.922137251	8.185847697	2.095416219	0.263710446
A_24_P122137	NM_002309	LIF	5.694479094	7.787720257	6.30256038	10.69735332	2.093241163	4.394792939
A_23_P8906	NM_013437	LRP12	8.726838501	10.81907758	9.920041649	11.52484174	2.092239082	1.604800094
A_23_P393425	NM_173797	PAPD4	10.92032026	13.01086526	10.34831692	10.23060573	2.090544997	-0.117711186
A_23_P359647	NM_138714	NFAT5	8.474783066	10.55862833	8.92144096	10.20331074	2.083845261	1.281869778
A_24_P149713	NM_014007	ZBTB43	7.372125514	9.455372111	8.372132035	9.310871393	2.083246596	0.938739357
A_24_P633902	NM_014455	RNF115	8.596196361	10.67254857	8.710650106	9.647218912	2.076352205	0.936568806
A_32_P225355	NM_182485	CPEB2	6.303130002	8.374689737	6.306919433	6.833832791	2.071559735	0.526913358
A_24_P167338	NM_014488	RAB30	6.598483919	8.664488732	5.552495251	5.713122674	2.066004813	0.160627423
A_24_P245379	NM_002575	SERPINB2	5.34314623	7.404140869	5.752081306	12.55669574	2.060994638	6.804614437
A_23_P210330	NM_014181	LGALSL	7.641555511	9.70233723	6.311564085	6.949289081	2.060781718	0.637724996
A_24_P750305	NR_015368	LOC643837	6.847431935	8.906141928	7.744899968	8.930169585	2.058709993	1.185269617
A_33_P3242543	NM_000240	MAOA	10.65349534	12.71133397	12.03782037	12.32848709	2.057838631	0.290666723
A_23_P408768	NM_032482	DOT1L	8.124185532	10.17855151	8.837325725	10.70341679	2.054365977	1.866091065
A_23_P383422	NM_139239	NFKBID	7.565933181	9.61711759	7.80106993	9.389030864	2.051184408	1.587960934
A_33_P3377691	NM_001008393	C4orf46	6.941227745	8.990465865	6.744683962	7.061455567	2.04923812	0.316771605
A_23_P350107	NM_030961	TRIM56	9.704487208	11.75329301	9.951114695	11.19837284	2.048805802	1.247258145
A_24_P168760	NM_004286	GTPBP1	8.998050695	11.04359781	9.197429138	9.824236889	2.045547115	0.62680775
A_23_P3532	NM_004862	LITAF	12.58320607	14.62762132	12.92461263	13.47793628	2.044415257	0.553323653
A_33_P3214314	NM_018999	FAM190B	9.184205044	11.22857876	9.226354023	10.48624187	2.044373712	1.259887847
A_33_P3231414	NM_006669	LILRB1	9.354668268	11.39856058	12.69791453	13.11005869	2.043892312	0.412144153
A_33_P3306192	NM_001101362	KBTBD13	8.985880451	11.02720034	9.092272101	9.621332189	2.041319893	0.529060088
A_33_P3279059	NM_003804	RIPK1	10.47600074	12.51700033	9.646228502	10.88856631	2.040999582	1.242337811
A_23_P76799	NM_013448	BAZ1A	10.59886691	12.63642425	9.98086628	11.57032183	2.037557333	1.589455553
A_23_P162288	NM_005379	MYO1A	7.028235889	9.065126126	6.208140081	6.873240186	2.036890238	0.665100105
A_33_P3273719	NM_001242831	ELOVL5	7.326825426	9.360775172	8.081812853	8.649687727	2.033949746	0.567874874
A_23_P338890	NM_002827	PTPN1	10.92679761	12.95728487	12.28933763	13.70165985	2.030487261	1.41232222
A_23_P90804	NM_145686	MAP4K4	7.540290434	9.567954014	7.20856218	8.13027523	2.02766358	0.92171305
A_23_P63209	NM_181755	HSD11B1	11.43985398	13.46385683	12.81257207	14.65102437	2.02400285	1.838452293
A_33_P3307163	NM_001134363	RBM20	7.873554266	9.896671711	6.963016699	8.204535705	2.023117444	1.241519005
A_23_P408094	NM_002357	MXD1	8.933398893	10.95598021	8.116731042	9.062750843	2.022581317	0.946019801
A_33_P3271111	NM_004148	NINJ1	14.4234432	16.44502116	15.51704128	17.07651093	2.021577965	1.559469643
A_24_P16124	NR_001590	IFITM4P	11.28831309	13.30332294	9.968851715	10.8139079	2.015009842	0.845056188
A_23_P75220	NM_031212	SLC25A28	9.007277914	11.02161247	9.587185258	10.08340116	2.014334556	0.496215905
A_24_P937405	NM_007173	PRSS23	5.399434671	7.411459968	5.525195174	5.862435092	2.012025297	0.337239918
A_23_P11685	NM_024420	PLA2G4A	9.63339307	11.64236871	9.797329471	10.419692	2.00897564	0.622362524
A_23_P116942	NM_002286	LAG3	6.856581402	8.865122776	7.164806108	8.689713003	2.008541374	1.524906895
A_24_P402898	NM_001102653	OTUD4	7.802969357	9.808266361	8.631298345	9.460059257	2.005297004	0.828760912
A_23_P147431	NM_002350	LYN	12.66001183	14.66319377	12.9384513	13.7597845	2.003181935	0.821333193
A_23_P370097	NM_001044385	TMEM237	7.224348963	9.224982445	7.257401571	8.391102105	2.000633482	1.133700534
A_23_P69179	NM_018192	LEPREL1	10.24583807	12.2422871	5.842454283	5.775761417	1.996449038	-0.066692866
A_33_P3371493	NM_003286	TOP1	7.557570222	9.550642777	7.53684304	8.584009586	1.993072554	1.047166547
A_23_P122197	NM_031966	CCNB1	7.755397274	9.746852289	7.948602521	9.393626407	1.991455014	1.445023886
A_24_P206343	NM_033054	MYO1G	8.922206545	10.91029824	9.373146815	9.769867349	1.988091691	0.396720534
A_33_P3320538		NUPL1	8.597989722	10.5858847	8.821364582	9.73711849	1.987894981	0.915753908
A_33_P3341722		COL23A1	9.847226278	11.83385704	10.10554297	10.00990211	1.986630762	-0.095640863
A_23_P321201	NM_015213	DENND5A	11.21384778	13.19816165	12.12554176	13.96762165	1.984313863	1.842079893
A_32_P228775	NM_174907	PPP4R2	8.081805675	10.06036086	8.298620482	9.80620745	1.978555184	1.507586968
A_23_P12526	NM_005426	TP53BP2	9.752925799	11.72809144	11.01276362	12.59953299	1.975165641	1.58676937
A_23_P87560	NM_001731	BTG1	11.40400004	13.37790834	11.86799467	13.1145352	1.973908307	1.246540527
A_24_P829183	XR_108575	FLJ32255	6.857162835	8.830974049	6.930435655	6.578969364	1.973811214	-0.351466291
A_32_P104000	NM_173475	DCUN1D3	10.7417346	12.71523526	9.923941122	10.74874367	1.973500656	0.824802548
A_24_P253251	NM_003045	SLC7A1	8.408793868	10.37649345	8.530454462	10.65249642	1.967699586	2.122041955
A_24_P649624	NM_183416	KIF1B	8.71519132	10.68090342	8.222397847	10.91558116	1.965712103	2.693183313
A_24_P258846	NM_172390	NFATC1	7.381165461	9.344522685	7.603159213	8.231275475	1.963357224	0.628116262
A_24_P941441	NM_006572	GNA13	11.42095561	13.38423518	11.89354755	12.21043216	1.963279563	0.316884617
A_33_P3286151	NR_033651	LOC100132891	7.488494158	9.450347808	7.950888884	9.284973491	1.961853651	1.334084607
A_23_P59950	NM_015359	SLC39A14	7.437905708	9.399498291	6.387323881	8.948558555	1.961592582	2.561234674
A_23_P368711	NM_006864	LILRB3	10.42924901	12.38897185	13.24677823	13.56719798	1.959722839	0.320419757
A_23_P52761	NM_002423	MMP7	7.938032751	9.895305902	9.306020328	11.88522577	1.957273151	2.579205442
A_23_P20275	NM_024613	PLEKHF2	11.19731234	13.15064182	10.23485106	11.52972934	1.953329478	1.29487828
A_33_P3352718		C12orf76	5.77957113	7.732686664	6.254404114	6.957605897	1.953115534	0.703201783
A_24_P567298	NM_004909	CSAG2	5.594950249	7.543194415	5.580429688	5.882012167	1.948244166	0.301582478
A_24_P127663	NM_024081	PRRG4	7.355547437	9.303303573	7.090908501	7.578297546	1.947756137	0.487389045
A_23_P319792	NM_019001	XRN1	8.777862179	10.72411234	8.419928549	8.751953693	1.946250164	0.332025144
A_33_P3316273	NM_002983	CCL3	14.79217842	16.73790375	14.51303264	17.58396462	1.945325326	3.07093198
A_24_P12690	NM_194294	IDO2	5.7076109	7.650528754	5.371931752	6.164226581	1.942917854	0.792294829
A_23_P368909	NR_027701	LINC00346	6.294920178	8.237349829	5.915291999	7.65719564	1.942429651	1.741903641
A_33_P3383871	NM_020177	FEM1C	9.835034309	11.77514452	9.262105213	9.832847522	1.940110211	0.570742309
A_23_P110569	NM_018700	TRIM36	8.593494227	10.53346916	6.576630179	8.530342775	1.939974933	1.953712596
A_23_P90497	NM_012276	LILRA4	9.56055463	11.49960247	12.55117603	12.82029747	1.939047837	0.269121437
A_23_P31873	NM_001002814	RAB11FIP1	11.49313852	13.43165388	11.34330606	10.02310789	1.938515355	-1.320198171
A_32_P19716	NM_001080470	ZNF697	8.238448672	10.17583033	9.25300024	10.97748776	1.937381655	1.72448752
A_24_P286079	NM_014423	AFF4	8.925840349	10.86260321	9.303974076	10.86382486	1.936762865	1.55985078
A_24_P305764	NM_004595	SMS	11.7709142	13.70751124	11.39836145	12.94813864	1.936597033	1.54977719
A_24_P63522	NM_002130	HMGCS1	9.319503122	11.25531318	9.261981157	10.86386251	1.935810058	1.601881357
A_24_P285768	NM_014674	EDEM1	8.60691842	10.54090646	8.64014993	10.37464863	1.93398804	1.734498696
A_24_P329353	NM_032831	ORAI2	7.601564163	9.535264993	7.129484222	8.748039577	1.93370083	1.618555356
A_23_P255444	NM_014395	DAPP1	9.897068857	11.82713988	8.856178853	8.49286097	1.930071023	-0.363317883
A_23_P371824	NM_020127	TUFT1	8.389641028	10.31759837	8.093280504	8.192959236	1.927957339	0.099678732
A_33_P3358342	NM_005067	SIAH2	11.50055743	13.42788516	11.87569856	12.09670121	1.927327732	0.221002643

A_23_P4662	NM_005178	BCL3	13.08748662	15.01376479	11.47923307	13.88496856	1.926278166	2.405735497
A_19_P00803697		XLOC_008632	6.017593901	7.942036211	6.013736204	7.473866184	1.92444231	1.46012998
A_23_P51231	NM_001031680	RUNX3	10.68809641	12.61005001	10.51825624	10.16394057	1.921953599	-0.354315662
A_23_P47691	NM_003141	TRIM21	8.529007287	10.45030539	8.222561371	9.283446946	1.921298103	1.060885575
A_24_P272290	NM_183373	C6orf145	9.328303228	11.24654023	8.614906083	8.733868566	1.918236999	0.118962483
A_23_P98310	NM_001326	CSTF3	9.76447033	11.67802495	9.038398082	9.301659366	1.913554617	0.263261284
A_23_P22433	NM_006915	RP2	11.52025582	13.43137361	11.41578593	12.47944395	1.911117783	1.063658027
A_24_P357465	NM_021202	TP53INP2	6.511863253	8.42188891	6.770262691	8.28743574	1.910025657	1.517173049
A_23_P120845	NM_005080	XBP1	13.33797132	15.24452946	13.37624375	14.60899877	1.906558145	1.23275502
A_23_P213699	NM_013982	NRG2	5.360223127	7.264945747	5.544296808	5.493065486	1.90472262	-0.051231321
A_24_P18146	NM_015310	PSD3	6.97303869	8.875138651	7.793080202	10.15400197	1.902099961	2.360921763
A_23_P89780	NM_198129	LAMA3	5.033709967	6.925471421	5.31817998	5.912239325	1.891761453	0.594059344
A_33_P3415923	NM_175736	FMNL3	9.538790475	11.42898865	9.73413554	10.66408054	1.890198178	0.929945
A_33_P3609431		ERVFRD-2	11.77807968	13.66807948	12.50943145	11.87118547	1.889999803	-0.638245977
A_24_P135276	NM_032172	USP42	10.16836877	12.05739791	9.896460828	10.76565816	1.889029136	0.869197335
A_23_P24176	NM_019084	CCNJ	8.632202394	10.50741158	8.529662774	10.03014984	1.875209183	1.500487067
A_24_P313186	NM_006888	CALM1	9.469610394	11.34386555	9.009515046	10.23388575	1.874255156	1.224370702
A_23_P357546	NM_015488	PNKD	7.272439107	9.146045659	8.66001373	9.042864493	1.873606552	0.382850762
A_24_P303454	NM_012454	TIAM2	7.893567144	9.76320435	7.263916084	9.25972065	1.869637206	1.995804566
A_24_P166663	NM_001259	CDK6	10.11482782	11.98419454	8.790200863	9.546035548	1.869366716	0.755834684
A_23_P143713	NM_021822	APOBEC3G	8.578714797	10.44806115	8.949262487	9.067248636	1.869346354	0.117986149
A_24_P115967	NM_006133	DAGLA	8.124297657	9.993177727	7.789137669	8.883996081	1.86888007	1.094858412
A_24_P274270	NM_139266	STAT1	10.31031037	12.17612439	10.07977776	11.88071526	1.865814023	1.800937493
A_23_P215154	NM_016118	NUB1	10.66755075	12.53111229	9.724699919	9.16030872	1.863561542	-0.564391199
A_23_P143016	NM_212481	ARID5A	11.99598964	13.85900657	11.77113669	12.16091175	1.863016937	0.389775057
A_23_P15414	NM_145352	SCARF1	9.586780633	11.44979251	9.648448814	10.25274977	1.863011881	0.604300954
A_33_P3331085	NM_021982	SEC24A	7.609265819	9.470386791	8.783974906	9.867293399	1.861120972	1.083318494
A_23_P356526	NM_033092	TRIM5	7.130631476	8.988990287	6.788696923	7.727311155	1.85835881	0.938614231
A_23_P427760	NM_152431	PIWIL4	8.05915273	9.916255486	7.598849585	8.84416014	1.857102756	1.245310555
A_24_P362540	NM_003887	ASAP2	6.529773367	8.385582474	9.584388533	10.94097683	1.855809107	1.356588301
A_23_P25566	NM_004951	GPR183	12.72686327	14.5787525	11.62791215	12.47094978	1.851889231	0.84303763
A_33_P3354945	NM_172210	CSF1	6.290558587	8.141116459	7.312802333	9.286135711	1.850557872	1.973333377
A_33_P3215953	NM_024569	MPZL1	8.668689734	10.51652076	8.714891963	10.28209787	1.84783103	1.567205904
A_32_P456318	NM_080743	SRSF12	5.236369262	7.083978085	5.204562961	6.764060745	1.847608823	1.559497784
A_23_P50508	NM_003706	PLA2G4C	10.04679985	11.88978022	8.530380446	9.172886174	1.842980369	0.642505728
A_23_P84565	NM_001722	POLR3D	7.202801487	9.045366916	7.778315971	8.885200993	1.842565429	1.106885022
A_33_P3352578	NM_080387	CLEC4D	6.367840949	8.209291652	6.938752206	6.279801566	1.841450703	-0.658950639
A_23_P84334	NM_018266	TMEM39A	7.66091944	9.502175882	7.416151632	9.135432008	1.841256442	1.719280376
A_19_P00316340		XLOC_006252	6.031374917	7.871812324	6.315304768	7.974976682	1.840437407	1.659671914
A_24_P374382	NR_001283	TOP1P2	8.919842463	10.7591608	8.958188112	10.36904972	1.839318333	1.410861611
A_24_P117029	NM_000527	LDLR	9.958778624	11.79674485	11.12425689	11.95541497	1.83796623	0.831158087
A_23_P14302	NR_026779	LINC00341	11.07200111	12.90989192	10.30151974	10.90828361	1.837890813	0.606763873
A_33_P3231888	NM_020199	C5orf15	9.832479301	11.66940944	9.670473407	9.645703673	1.836930142	-0.024769734
A_24_P252043	NM_003463	PTP4A1	6.527459922	8.360492208	7.538450869	7.681429054	1.833032286	0.142978185
A_24_P241815	NM_002229	JUNB	8.57689474	10.40947757	10.48251114	10.38134326	1.83258283	-0.101167879
A_23_P326204	NM_152621	SGMS2	6.515366934	8.33731605	7.020772226	8.818085312	1.821949117	1.797313086
A_23_P109026	NM_022358	KCNK15	11.91204329	13.7328115	11.5738946	13.4081694	1.820768207	1.834274803
A_33_P3354569	NM_001083112	GPD2	8.801019736	10.62158446	9.231667356	9.768845861	1.820564728	0.537178504
A_23_P213385	NM_006317	BASP1	13.44859605	15.26282421	14.02323465	14.64856043	1.814228161	0.625325777
A_33_P3263232	NM_030891	LRRC3	8.466036466	10.27232074	6.92722855	8.368975732	1.806284271	1.441747182
A_23_P89431	NM_002982	CCL2	11.07232531	12.87480852	9.624621829	14.38882768	1.802483215	4.764205854
A_33_P3514859	XR_108862	LOC100506342	6.603869026	8.404067044	6.541081972	8.210187655	1.800198019	1.669105683
A_33_P3363091		VAC14	6.911363607	8.710713038	7.510433406	7.779055692	1.799349431	0.268622286
A_33_P3228593		WDR45	6.61266957	8.407604051	6.690881211	7.4048225	1.794934481	0.71394129
A_33_P3300308	NM_032514	MAP1LC3A	7.253798685	9.048460107	6.809630283	8.793966602	1.794661422	1.984336319
A_23_P327519	NM_139164	STARD4	8.093550791	9.886911897	8.745176794	9.653797883	1.793361106	0.908621089
A_33_P3267799	NM_006847	LILRB4	12.06669369	13.85626649	14.96784452	15.64038984	1.789572802	0.672545323
A_23_P22682	NM_016608	ARMCX1	9.08452815	10.87237026	8.312553775	9.651813537	1.787842113	1.339259762
A_23_P329870	NM_024599	RHBDF2	10.98774216	12.77522339	12.93812822	13.1679713	1.787481226	0.229843087
A_24_P359545	NM_019610	RBMXL1	8.071513262	9.858308573	7.443816495	8.798797752	1.786795311	1.354981257
A_24_P110799	NM_002340	LSS	9.772417747	11.55505989	8.831993085	10.8161506	1.782642143	1.984157519
A_23_P203299	NM_002901	RCN1	9.624577303	11.40678698	10.18303917	11.2205079	1.782209677	1.03746873
A_23_P315571	NM_015150	RFTN1	12.83245548	14.61129654	12.16733245	13.63218307	1.778841057	1.464850613
A_23_P356330	NM_015704	PPPDE2	9.345778702	11.12452507	9.90323527	10.69035456	1.778746368	0.787119287
A_19_P00327406		XLOC_007052	6.53819831	8.314293033	6.470463253	8.629102193	1.776094723	2.15863894
A_23_P114057	NM_017789	SEMA4C	7.674490173	9.449784506	10.66525672	10.82708264	1.775294333	0.16182592
A_23_P145584	NM_003344	UBE2H	8.186429825	9.961366354	8.668579497	9.231988718	1.774936529	0.563409221
A_33_P3325843		FLJ40039	6.962474603	8.735671511	6.911577327	8.037938706	1.773196908	1.12636138
A_23_P91640	NM_020437	ASPHD2	8.621403428	10.39198118	7.473716197	7.544854068	1.770577756	0.071137872
A_33_P3366053	NM_001125	ADPRH	10.33669562	12.10419852	9.964249163	10.25919339	1.767502906	0.294944223
A_24_P288754	NM_002641	PIGA	8.218059411	9.984600434	8.505636394	9.81129379	1.766541023	1.305657396
A_23_P2181	NM_016229	CYB5R2	6.571426521	8.335043885	9.05239499	10.58436274	1.763617364	1.531967754
A_33_P3325355	NM_006809	TOMM34	11.66098411	13.42430844	10.90459087	11.95126177	1.763324328	1.046670903
A_23_P166408	NM_020530	OSM	10.39717689	12.16040023	6.790955284	11.14722623	1.76322334	4.356270949
A_23_P30972	NM_022091	ASCC3	7.854591401	9.61621664	7.780545252	8.832035864	1.761625239	1.051490612
A_23_P32233	NM_004235	KLF4	9.395200959	11.15588314	9.615134252	9.48719893	1.760682181	-0.127935321
A_23_P8961	NM_000880	IL7	6.691603927	8.451078286	5.951512996	7.302472045	1.75947436	1.350959049
A_23_P432034	NM_173510	CCDC117	9.262135476	11.02044882	9.376762697	9.575140376	1.75831334	0.198377679
A_24_P734720	NM_152523	CCNYL1	7.297964406	9.056052896	6.935404819	7.959205844	1.75808849	1.023801025
A_33_P3587376	NR_024214	SNAR-A3	11.71641874	13.47325823	11.62593202	12.94194136	1.756839483	1.316009337
A_23_P75741	NM_198183	UBE2L6	10.37469841	12.12298617	11.16536222	11.43472152	1.748287759	0.269359303
A_23_P152082	NM_016642	SPTBN5	6.175847487	7.922269504	5.983950013	6.555431561	1.746422017	0.571481548
A_24_P270829	NM_000173	GP1BA	6.274403392	8.017530746	6.266467464	6.278451053	1.743127354	0.011983589
A_24_P405054	NM_015609	C1orf144	9.678032665	11.41852347	9.584983287	10.36897183	1.740490802	0.783988546
A_23_P106362	NM_020980	AQP9	11.38263928	13.12227594	14.17987781	16.02562792	1.73963666	1.845750113
A_32_P98072	NM_007113	TCHH	6.300423069	8.037620258	5.934601205	6.167585802	1.73719719	0.232984597
A_24_P126060	NM_001356	DDX3X	10.10050877	11.8345277	10.2058422	10.21695228	1.734018924	0.011110074
A_33_P3265189	NM_001942	DSG1	5.685420118	7.418815475	5.563239548	6.936456897	1.733395358	1.373217349
A_33_P3376971	NM_024111	CHAC1	7.242696614	8.975279647	7.42405852	7.441757327	1.732583033	0.017698807
A_33_P3267356		GLS	9.393122532	11.12129549	8.56042195	9.605216192	1.728172958	1.044794242
A_33_P3272402	NM_139174	ADAD2	6.375715987	8.103527184	6.756576292	7.771917532	1.727811197	1.015341239
A_23_P373079	NM_152716	PATL1	9.458504985	11.18544977	10.71536959	10.45132428	1.726944785	-0.264045307
A_33_P3209433	NM_021975	RELA	8.610355886	10.33725512	9.054774285	9.667654862	1.726899234	0.612880577
A_33_P3386716	NM_032833	PPP1R15B	11.44791817	13.17328139	11.67571505	12.25675111	1.725363223	0.581036063
A_23_P97457	NM_022831	AIDA	7.291886233	9.015430868	8.258362359	8.822196176	1.723544635	0.563833818
A_23_P25735	NM_002791	PSMA6	14.48314497	16.20218004	14.51992817	15.84429929	1.719035067	1.32437112
A_23_P166	NM_145279	MOB3C	9.511677079	11.22897589	8.788594504	9.493203372	1.717298811	0.704608867
A_23_P9293	NM_004817	TJP2	10.408485	12.12505566	10.12742827	11.55336515	1.716570659	1.425936879
A_23_P214139	NM_002912	REV3L	8.126932027	9.839688177	7.994638607	10.03890834	1.71275615	2.044269736
A_32_P18440	NM_032199	ARID5B	9.390993002	11.10349185	8.528065175	9.626406293	1.712498852	1.098341118
A_33_P3338300	NM_001079843	CASZ1	6.7026108	8.414873047	6.219358864	6.597040407	1.712262247	0.377681543

A_33_P3402035	NM_004295	TRAF4	7.599204929	9.305619978	7.157719332	8.367010576	1.706415049	1.209291243
A_33_P3298105	NM_001166271	SPATA13	6.204860936	7.909223367	7.356225553	8.274637903	1.70436243	0.91841235
A_24_P300302	NR_026867	ZNF300P1	5.412713421	7.116328171	5.673822969	6.665795223	1.70361475	0.991972254
A_23_P106389	NM_003612	SEMA7A	7.178916194	8.882160131	7.123050917	6.232145935	1.703243938	-0.890904982
A_23_P128166	NM_014999	RAB21	11.6537424	13.35512756	11.84098357	12.30608282	1.701385167	0.465099257
A_23_P84929	NM_033518	SLC38A5	6.349509755	8.048645053	7.410997528	8.028239622	1.699135298	0.617242094
A_23_P202117		PCGF5	8.855054119	10.5540948	8.708462705	9.491633025	1.699040681	0.78317032
A_24_P940803	NM_033505	EPT1	7.756952025	9.450344765	7.983877541	9.173580435	1.693392739	1.189702894
A_33_P3268567	NM_003581	NCK2	10.63103424	12.32441798	11.95079912	13.39659336	1.693383743	1.445794243
A_23_P34628	NM_001514	GTF2B	10.69364844	12.38368973	10.47452579	12.01969062	1.690041291	1.54516483
A_23_P160618	NM_003975	SH2D2A	8.208654203	9.896241856	8.733024966	10.08072268	1.687587652	1.347697712
A_24_P393958	NM_007034	DNAJB4	7.099037306	8.78583752	6.464765044	7.534345241	1.686800214	1.069580197
A_23_P34568	NM_017825	ADPRHL2	9.604746857	11.29000569	9.155544542	10.47130232	1.685258833	1.315757777
A_23_P106002	NM_020529	NFKBIA	14.53745283	16.22099953	13.68361021	16.05120278	1.683546698	2.367592567
A_23_P37424	NM_024063	SPATA5L1	9.915175107	11.59510468	9.878259399	10.37977071	1.679929573	0.501511311
A_33_P3349843		MGAT1	6.70840649	8.387965573	8.568519104	9.220526036	1.679559083	0.652006932
A_24_P588897	NM_013272	SLCO3A1	8.677133322	10.35617032	7.699124517	9.444851425	1.679037002	1.745726907
A_23_P30223	NM_001047	SRD5A1	8.429146386	10.10752143	8.022284557	8.193958179	1.678375044	0.171673622
A_23_P252541	NM_177403	RAB7B	12.40143566	14.07919068	9.39778322	11.04389949	1.677755019	1.646116267
A_24_P116805	NM_213662	STAT3	8.217441122	9.893230618	8.661740181	9.110564695	1.675789496	0.448824514
A_23_P54929	NM_020424	LYRM1	8.341889813	10.01472859	8.276754986	9.546351443	1.672838774	1.269596457
A_23_P304171	NM_001145642	KIAA0226	10.88947381	12.55898073	9.834930949	10.78373098	1.669506923	0.948800028
A_33_P3224710	NM_012252	TFEC	12.25063546	13.91927568	11.35753122	11.51859981	1.668640227	0.161068597
A_24_P42501	NM_001037171	ACOT9	10.06735994	11.73432545	10.01842894	10.93421888	1.666965507	0.915789945
A_33_P3346826	NM_001012633	IL32	7.175640204	8.841325632	6.67278218	7.662964833	1.665685428	0.990182653
A_33_P3316539	NM_001008539	SLC7A2	5.578336487	7.243520005	8.185713934	10.37925282	1.665183518	2.193538882
A_24_P184732	NM_173462	PAPLN	6.678841142	8.341962894	6.702145399	7.339210507	1.663121752	0.637065109
A_23_P214222	NM_002356	MARCKS	13.6652118	15.3281146	10.470228	14.47579967	1.662902804	4.005571669
A_24_P305570	NM_018993	RIN2	11.09542697	12.7553134	10.66000363	12.01332826	1.659886427	1.35332463
A_23_P157495	NM_005605	PPP3CC	9.600897874	11.25968599	9.369487784	10.85028054	1.658788112	1.480792756
A_33_P3240702	NM_002894	RBBP8	8.794105128	10.45081028	7.649450914	9.355223576	1.656705149	1.705772662
A_23_P308032	NM_005387	NUP98	9.80300431	11.45838217	8.686750863	9.981102734	1.655377863	1.29435187
A_24_P915196	NM_153045	C9orf91	8.464114869	10.11767591	9.019031016	9.171691428	1.653561046	0.152660412
A_32_P489130	NM_153252	BRWD3	6.417619472	8.068596456	6.650245838	6.692800174	1.650976983	0.042554336
A_23_P42257	NM_003897	IER3	13.49348388	15.14427748	9.336066294	14.51380754	1.650793597	5.177741246
A_23_P132121	NM_173354	SIK1	7.337582818	8.986878644	7.624622415	7.450341594	1.649295827	-0.17428082
A_24_P100996	NM_213599	ANO5	5.810623126	7.458575401	5.761035877	8.036195304	1.647952275	2.275159427
A_33_P3395369	NM_001634	AMD1	10.05634235	11.70369695	9.540018105	10.74610139	1.647354592	1.206083285
A_23_P212545	NM_004162	RAB5A	11.56072131	13.20770562	11.61141595	12.31344212	1.64698431	0.702026177
A_24_P355267	NM_001006641	SLC25A25	10.45408458	12.09967217	10.94956938	11.3934884	1.645587585	0.44391902
A_23_P426663	NM_198159	MITF	10.78403083	12.42589562	12.47517751	12.32190581	1.641864791	-0.1532717
A_23_P409623	NM_003621	PPFIBP2	10.31696051	11.95756526	9.61011259	11.86812718	1.640604749	2.258014593
A_33_P3359061	NM_198537	YJEFN3	5.619759712	7.258430236	5.858371697	7.556426805	1.638670524	1.698055108
A_23_P58132	NM_004310	RHOH	6.760418493	8.398683366	6.60537679	7.987939948	1.638264872	1.382563158
A_23_P14083	NM_181847	AMIGO2	5.909135544	7.54068227	5.439513996	6.825119524	1.631546725	1.385605528
A_24_P53051	NM_171846	LACTB	9.898232534	11.52955516	10.3868721	10.97444412	1.631322622	0.587572027
A_23_P385217	NM_018184	ARL8B	12.61205104	14.24060031	12.80754584	14.29240182	1.628549273	1.48485598
A_24_P248606	NM_004457	ACSL3	9.437391834	11.06454434	8.487276217	9.445907677	1.627152506	0.95863146
A_33_P3362088	NM_002560	P2RX4	12.87999541	14.50670305	13.30403577	14.13023682	1.626707643	0.82620105
A_24_P372134	NM_018295	TMEM140	8.212164302	9.838434258	9.768335918	9.507212432	1.626269957	-0.261123485
A_23_P32785	NM_173824	C3orf38	9.213228458	10.83728165	9.299573302	9.938586172	1.624053192	0.63901287
A_24_P74559	NM_013385	CYTH4	7.549500307	9.170848084	8.56149874	7.893509856	1.621347777	-0.667988884
A_23_P48056	NM_006825	CKAP4	8.719187623	10.33631459	11.63856833	12.09975459	1.617126968	0.461186253
A_23_P214587	NM_003449	TRIM26	9.199713177	10.81572303	8.484691753	9.290822993	1.616009857	0.80613124
A_23_P16944	NM_001006946	SDC1	7.647169426	9.262841422	7.723845059	8.579716099	1.615671996	0.85587104
A_23_P73609	NM_000266	NDP	5.529079776	7.14361655	6.020995914	7.733910205	1.614536774	1.712914291
A_23_P32707	NM_012291	ESPL1	7.528375519	9.141354449	7.535164321	8.390540752	1.61297893	0.855376431
A_23_P205789	NM_002041	GABPB1	8.504861731	10.11622136	8.885665367	10.18175272	1.611359627	1.296087348
A_33_P3264042	NM_001144933	EFCAB3	6.247977566	7.859038382	6.951488866	9.011159527	1.611060816	2.059670661
A_33_P3318292	NM_005066	SFPQ	11.47117379	13.07962537	9.570739889	11.8857792	1.608451576	2.315039308
A_33_P3300680	NM_031448	C19orf12	8.763711917	10.37031916	8.862443488	9.363519296	1.606607243	0.501075808
A_33_P3268304	NM_001161404	LIMS2	10.93015113	12.53360276	10.482843	9.390417504	1.603451626	-1.092425499
A_23_P163458	NM_139265	EHD4	11.49041471	13.08296954	10.63706786	12.04429625	1.592554827	1.407228392
A_23_P418031	NM_001136265	IFFO2	10.01111025	11.60035934	9.622895596	8.398656364	1.589249094	-1.224239232
A_24_P262127	NM_004165	RRAD	5.29514991	6.884300186	5.962022625	10.67345116	1.589150275	4.711428538
A_33_P3313555	NM_001042414	PSPC1	8.511932907	10.10067837	8.408369055	9.361544588	1.58874546	0.953175533
A_23_P371865	NM_152342	CDYL2	6.61901207	8.207051852	6.266578683	6.889025765	1.588039782	0.622447083
A_24_P115774	NM_001166	BIRC2	10.12332244	11.71067591	9.642076705	10.36106109	1.587353466	0.718984386
A_33_P3217393	NM_001024736	CD276	8.400645873	9.987282238	8.136828817	8.673373662	1.586636365	0.536544845
A_23_P131240	NM_181713	UBXN2A	6.664334334	8.249290767	6.901980641	7.927474724	1.584956433	1.025494084
A_24_P923251	NM_198951	TGM2	8.007586313	9.590527395	10.95627619	12.26134701	1.582941082	1.305070823
A_33_P3368452	NM_000247	MICA	9.123211153	10.70494031	9.058210963	9.765625008	1.58172916	0.707414045
A_23_P111132	NM_005345	HSPA1A	13.40329945	14.98333126	12.17427707	14.32283199	1.580031807	2.148554917
A_33_P3407675	NM_001031800	TIPRL	9.428831498	11.00773944	9.271056469	9.935680698	1.578907938	0.664624229
A_23_P160460	NM_003115	UAP1	8.235932342	9.81209389	8.972634084	10.37343294	1.576161549	1.400798856
A_33_P3279831	NM_006813	PNRC1	11.81738757	13.39168261	11.29374211	13.12792118	1.574295044	1.83417907
A_23_P160214	NM_001080494	TTC39A	5.150639199	6.723061668	5.11262455	6.157983255	1.572422469	1.045358705
A_24_P139901	NM_002101	GYPC	8.643584481	10.21226887	10.15739336	10.55322691	1.568684385	0.395833557
A_24_P94054	NM_006282	STK4	9.72479991	11.29258323	8.843636563	9.214398272	1.56778332	0.370761709
A_23_P24244	NM_017782	FAM208B	7.764993868	9.331637444	7.404572709	7.9457448	1.566643575	0.541172092
A_23_P113005	NM_004428	EFNA1	6.509179733	8.073997584	7.253823764	7.422438467	1.564817851	0.168614704
A_23_P68970	NM_014570	ARFGAP3	11.2500433	12.8134008	12.44402988	14.00853893	1.563357498	1.564509047
A_23_P88678	NM_152335	C15orf27	9.021710668	10.58506754	9.862493129	9.220118314	1.563356874	-0.642374816
A_23_P31073	NM_005375	MYB	7.189786783	8.752047472	5.613570279	5.803715337	1.56226069	0.190145058
A_33_P3344477	NM_001134999	FERMT2	5.686130624	7.24438113	5.601965865	7.692216532	1.558250506	2.090250667
A_33_P3408305	NM_178842	CERS3	10.1257014	11.68279303	9.835063465	10.76433191	1.557091635	0.929268442
A_23_P134744	NM_024787	RNF122	9.07647279	10.63096567	10.14090938	10.70329651	1.554492883	0.562387123
A_33_P3227041	NM_197966	BID	13.80460537	15.35726899	13.74950185	15.94210163	1.552663627	2.19259978
A_32_P171328	NM_014501	UBE2S	10.61664194	12.16885675	10.12015703	10.91873306	1.552214809	0.798576033
A_24_P183094	NM_024524	ATP13A3	12.55833632	14.10465739	12.2683925	13.47543474	1.546321075	1.207042247
A_23_P424734	NM_178516	EXOC3L1	6.647341056	8.193005543	6.450755477	6.919544272	1.545664487	0.468788795
A_23_P103110	NM_012323	MAFF	9.562881794	11.10570596	11.82018451	12.19526742	1.54282417	0.375082917
A_33_P3410507	NM_014812	CEP170	9.287112355	10.82521454	9.471750425	9.958527684	1.538102182	0.486777259
A_23_P1782	NM_002231	CD82	10.24463492	11.77646771	12.50637939	13.80899756	1.531832784	1.302618173
A_23_P63190	NM_002524	NRAS	9.319239608	10.84983058	9.178066248	9.860170216	1.530590968	0.682103968
A_24_P349039	NM_020754	ARHGAP31	9.301212087	10.82952433	8.493492368	9.736706039	1.528312243	1.24321367
A_33_P3741059	XR_110492	LOC100506459	7.351226444	8.878197998	6.363268372	6.935715658	1.526971554	0.572447287
A_24_P271696	NM_001097592	XAGE1A	5.88167926	7.408622354	5.860203894	6.187071187	1.526943094	0.326867293
A_24_P23411	NM_016607	ARMCX3	8.564516345	10.0912933	8.431846921	9.842409822	1.52677696	1.410562901
A_33_P3323607		CCDC129	7.814758569	9.33914589	7.300387376	8.815791356	1.524387321	1.51540398
A_23_P351215	NM_005414	SKIL	7.181744816	8.705736322	6.946667179	7.995257744	1.523991506	1.048590565

A_24_P48177	NM_006927	ST3GAL2	10.64266114	12.16480034	10.43402774	10.97141809	1.522139196	0.537390357
A_23_P307502	NR_024274	C9orf53	6.869263891	8.389703194	7.303308023	8.131739522	1.520439303	0.828431499
A_33_P3320772	NM_000332	ATXN1	7.544314455	9.064488704	7.298192001	7.948590708	1.52017425	0.650398707
A_24_P55496	NM_053001	OSR2	5.88713961	7.405479175	5.867382812	6.268359487	1.518339565	0.400976675
A_23_P259413	NM_017548	CDV3	11.25806164	12.77608399	11.97561429	12.26319125	1.518022346	0.287576953
A_23_P200001	NM_144573	NEXN	6.654670577	8.172110063	6.989444787	8.309322187	1.517439486	1.3198774
A_23_P384329	NM_001144823	DENND4A	5.766828751	7.284041011	5.748830971	6.433198055	1.517212261	0.684367085
A_33_P3222788	NR_024467	LOC100188947	7.080133902	8.589370372	7.026287826	7.40508551	1.50923647	0.378797684
A_23_P120153	NM_173647	RNF149	12.46785992	13.97636027	12.60930747	12.44094018	1.508500345	-0.168367287
A_33_P3230219	NM_033504	TMEM54	6.606743585	8.112643119	6.710203251	7.794518006	1.505899534	1.084314755
A_23_P380318	NM_001965	EGR4	6.373783463	7.874295715	5.695328862	5.762525071	1.500512253	0.067196209
A_23_P47790	NM_005371	METTL1	9.873404045	11.37386153	10.41743461	12.35800523	1.500457485	1.940570618
A_32_P193322	NM_152756	RICTOR	8.888601789	10.38514533	9.167852195	9.368161916	1.496543537	0.200309721
A_23_P336198	NM_138426	GLCCI1	6.687622559	8.183813847	6.559291227	5.947145838	1.496191288	-0.612145389
A_23_P123539	NM_002717	PPP2R2A	10.25760521	11.74648395	11.00457299	11.13379614	1.488878741	0.129223153
A_23_P99741	NM_004196	CDKL1	6.093913668	7.582720277	6.899862777	6.753630428	1.488806609	-0.146232349
A_23_P36226	NM_032315	SLC25A33	10.0368964	11.51843063	9.521881232	11.66242039	1.481534238	2.140539158
A_23_P86653	NM_002727	SRGN	14.78077422	16.26148481	15.10213516	16.50168453	1.480710592	1.399549367
A_24_P303647	NM_152556	C7orf60	8.255234439	9.735320139	8.170993298	9.859179215	1.4800857	1.688185916
A_24_P12401	NM_001025366	VEGFA	7.146607804	8.62622293	10.35796331	10.13661861	1.479615126	-0.221344701
A_23_P336992	NM_182491	ZFAND2A	8.337529256	9.81403155	9.317400807	10.53576547	1.476502293	1.218364667
A_24_P147461	NM_001031848	SERPINB8	8.684765831	10.1606736	7.767825816	9.382008	1.475907768	1.614182184
A_23_P300056	NM_044472	CDC42	6.630816499	8.105290603	6.562208019	7.738164792	1.474474104	1.175956773
A_23_P38365	NM_006852	TLK2	9.671274622	11.14472136	9.764489419	10.79903574	1.473446734	1.034546317
A_32_P806841	NM_005738	ARL4A	6.88070551	8.353844704	7.03049668	7.733226094	1.473139193	0.702729414
A_24_P813147	NM_177987	TUBB8	9.429734147	10.9027801	8.606901514	10.04147263	1.47304595	1.434571113
A_33_P3404331	NM_015093	TAB2	8.197005052	9.6694919	9.175381056	8.972242431	1.472486848	-0.203138626
A_33_P3260016	NM_152905	NEDD1	10.76868165	12.24002477	10.5403064	10.45597396	1.471343125	-0.084332433
A_33_P3243069	NM_001162893	KIAA0040	10.07069741	11.54092631	9.793453351	10.14470107	1.470228906	0.351247722
A_23_P330895	NR_040515	NFKBIB	7.829411776	9.298569898	7.754298913	9.046694284	1.469158122	1.292395371
A_19_P00809068		XLOC_014327	6.79409007	8.262610247	6.739258318	7.810098407	1.468520176	1.070840089
A_33_P3399870	NM_203463	CERS6	9.545535948	11.01083317	9.287792055	9.502822894	1.465297222	0.21503084
A_23_P146066	NM_003114	SPAG1	5.675699266	7.139966043	5.550955962	7.768190024	1.464266778	2.217234062
A_23_P75430	NM_020179	C11orf75	13.27472804	14.73819524	13.29378778	14.04968161	1.463467203	0.755893823
A_23_P66543	NM_014308	PIK3R5	7.389284614	8.848826942	7.572608321	7.905760628	1.459542328	0.333152307
A_33_P3807268	NR_033424	DNAJA1P5	11.48539047	12.94439882	10.8180613	11.70729617	1.459008345	0.889234867
A_23_P45087	NM_016220	ZNF107	6.987153152	8.446023971	6.18868759	7.21310601	1.458870819	1.02441842
A_33_P3219475	NM_001198812	C9orf30-TMEFF1	6.153029279	7.611807048	6.770976742	8.297050733	1.458777769	1.526073991
A_23_P7791	NM_024576	OGFRL1	12.08846254	13.54472335	11.85290431	13.11456654	1.456260806	1.26166223
A_24_P82880	NM_003290	TPM4	11.6542789	13.10989441	11.48964383	12.75840978	1.455615514	1.268765943
A_23_P209805	NM_005966	NAB1	9.24461325	10.69763718	7.947756633	8.711559253	1.45302393	0.763802619
A_33_P3237225	NM_152231	FBXO34	9.703391573	11.15527531	9.859308552	10.56785395	1.451883733	0.708545395
A_23_P148484	NM_016120	RLIM	8.954759527	10.40651997	8.556061239	9.207088429	1.451760443	0.65102719
A_33_P3275235	NM_005163	AKT1	11.47492287	12.92650204	11.30100042	12.53987873	1.45157917	1.238878307
A_23_P5611	NM_018151	RIF1	7.674976844	9.124420446	7.670679457	8.92732855	1.449443602	1.256649093
A_23_P415401	NM_001206	KLF9	11.08891804	12.53821447	12.33151259	12.16091693	1.44929643	-0.170595663
A_23_P128174	NM_175623	RAB3IP	7.899697018	9.347958923	8.545281333	9.704578766	1.448261904	1.159297433
A_24_P169688	NM_005931	MICB	7.378115122	8.825889884	8.101879775	8.061602082	1.447774762	-0.040277693
A_24_P233786	NM_052966	FAM129A	13.01188106	14.45847061	12.81163008	13.35000982	1.446589549	0.538379743
A_23_P218111	NM_001002236	SERPINA1	11.58866884	13.03456781	12.12300075	12.05103403	1.445898972	-0.07196672
A_33_P3245415	NM_052818	N4BP2L1	8.693983365	10.13886177	9.050674343	10.98164053	1.444878401	1.930966187
A_33_P3312735	NM_024989	PGAP1	6.111795049	7.553032667	6.157039496	6.942195528	1.441237618	0.785156032
A_33_P3340718	NM_021140	KDM6A	8.784580081	10.2257417	8.483733406	9.10687461	1.441161616	0.623141204
A_23_P54376	NM_004809	STOML1	9.82325817	11.26330156	8.795642811	9.568867206	1.440043387	0.773224395
A_23_P143120	NM_003183	ADAM17	9.472330197	10.91053793	9.512879633	10.42797674	1.438207736	0.915097103
A_23_P250982	NM_016048	ISOC1	10.45885785	11.89423352	10.10272375	10.89396635	1.43537567	0.791242601
A_24_P70002	NM_014572	LATS2	9.292561318	10.72723886	8.983797029	9.810361449	1.434677538	0.82656442
A_33_P3294017	NM_031314	HNRNPC	10.4556638	11.88731455	10.1692702	11.28617144	1.431650749	1.116901233
A_33_P3278941	NM_001048205	REC8	8.85719138	10.28454987	8.870016977	9.794147448	1.427358495	0.924130471
A_33_P3400943	NM_001199054	C16orf5	10.69110123	12.11754622	10.3827741	9.840711184	1.426444987	-0.542062915
A_24_P212481	NM_024717	MCTP1	6.69439854	8.12077916	6.780297712	8.539638469	1.42638062	1.759340757
A_33_P3231277	NM_181054	HIF1A	8.256673808	9.681066956	8.069378858	10.58328178	1.424393148	2.513902925
A_24_P16913	NM_005845	ABCC4	6.337505899	7.759862774	5.998927955	6.329853172	1.422356875	0.330925217
A_33_P3409849		C12orf63	5.24793854	6.666494483	5.394712836	5.710505414	1.418555943	0.315792578
A_23_P15844	NM_032043	BRIP1	5.51991374	6.933562625	5.462014216	5.552079016	1.413648885	0.0900648
A_23_P93524	NM_001017373	SAMD3	5.47611804	6.888018312	5.297959168	6.351924963	1.411900272	1.053965795
A_23_P500956	NM_006577	B3GNT2	7.936446945	9.347812858	8.475389533	8.964888108	1.411365914	0.489498574
A_33_P3409513	NM_002751	MAPK11	8.630541636	10.04090603	6.682618267	7.71663942	1.410364398	1.034021153
A_23_P361569	NM_025181	SLC35F5	9.143657454	10.54962009	8.6120368	10.87767285	1.40596264	2.265636047
A_33_P3238785	NR_024499	FMR1-AS1	5.941658014	7.347465013	6.282263703	6.470697237	1.405806999	0.188433534
A_24_P398130	NM_014688	USP6NL	6.912788333	8.317628163	6.4997399	7.514951289	1.40483983	1.015211388
A_33_P3256680	NM_004225	MFHAS1	6.55654622	7.961077368	7.755722536	9.048778934	1.404531148	1.293056399
A_23_P120316	NM_006636	MTHFD2	12.19214583	13.59446873	12.47005076	13.59084291	1.402322903	1.12079215
A_33_P3325978		TRIB2	7.125359684	8.52455466	7.313245904	6.865470846	1.399194976	-0.447775058
A_24_P409494	NM_015367	BCL2L13	7.695903225	9.093611699	8.386553995	9.047971129	1.397708474	0.661417135
A_23_P115407	NM_146421	GSTM1	7.659585922	9.054930294	8.248709044	8.208695053	1.395344372	-0.04001399
A_23_P63870	NM_144660	SAMD8	7.059111774	8.450994111	7.434093694	8.189081707	1.391882338	0.754988013
A_33_P3281795	NM_007283	MGLL	11.10677946	12.49585623	10.330373	13.06304461	1.389076768	2.732671617
A_23_P111672	NM_152829	TES	7.242859275	8.631912668	6.890005597	7.281320112	1.389053393	0.391314515
A_19_P00321670		XLOC_005730	8.028333827	9.417178564	7.422497706	8.831912808	1.388844738	1.409415102
A_19_P00318443		XLOC_006507	5.826561132	7.212388558	6.163704196	6.501385338	1.385827426	0.337681142
A_23_P62932	NM_001677	ATP1B1	13.56593537	14.94992213	12.27565994	14.84935885	1.383986753	2.573698913
A_23_P92230	NM_014779	TSC22D2	7.362185451	8.745419533	7.817114929	7.598152019	1.383234082	-0.21896291
A_23_P35256	NM_006468	POLR3C	9.207174055	10.58872555	9.57485772	10.48208277	1.381551498	0.907225053
A_33_P3250840	NM_144599	NIPA1	9.519685233	10.8988374	8.366142483	10.25696004	1.379152164	1.890817553
A_24_P48898	NM_145637	APOL2	12.52752254	13.9063915	12.24061581	12.75372416	1.378868965	0.51310835
A_23_P60248	NM_003329	TXN	15.18076102	16.55757391	14.29918069	15.66095098	1.376812887	1.36177029
A_33_P3296687	NM_004138	KRT33A	6.413642684	7.790200197	6.383554367	6.620667375	1.376557513	0.237113008
A_33_P3214849	NM_153705	KDELC2	8.640773982	10.01684673	7.223639427	8.61215504	1.37607275	1.388515613
A_23_P106682	NM_001424	EMP2	6.326546938	7.701951295	6.223863316	6.243300675	1.375404357	0.019437359
A_24_P128312	NM_007135	ZNF79	7.924309687	9.296682614	7.300499805	7.342878428	1.372372926	0.042378623
A_24_P68908	NR_033752	LOC344887	8.367622471	9.738235598	7.170997648	8.721057813	1.370613128	1.550060165
A_32_P98298	NM_004420	DUSP8	6.932469419	8.302626366	7.219652956	8.466701915	1.370156947	1.247048959
A_24_P573533	NM_001024916	CBWD5	6.645040033	8.014333309	6.810954881	7.422352649	1.369293276	0.611397768
A_24_P212539	NM_138801	GALM	9.349160945	10.71823731	9.593574382	9.233223833	1.369076362	-0.360350549
A_23_P120566	NM_001042576	RRBP1	9.458424484	10.82711334	9.465715223	10.18181355	1.368688856	0.716098324
A_23_P125265	NM_002266	KPNA2	12.95193503	14.3198685	12.03513483	13.56019	1.367933475	1.525055173
A_23_P203023	NM_002906	RDX	8.58266823	9.950324924	9.397389906	10.28274004	1.367656694	0.885350138
A_23_P153197	NM_170695	TGIF1	9.649614421	11.01545817	9.718521797	9.881607446	1.365843749	0.16308565
A_33_P3290573	NM_017633	FAM46A	9.023800033	10.38837571	10.22047744	10.44276518	1.364575679	0.222287742
A_33_P3272823	NM_002361	MAG	7.703913022	9.067584325	8.382519172	10.09747807	1.363671303	1.714958902

A_33_P3236646	NM_198551	MIA3	10.14579445	11.50838034	10.39375448	10.72378507	1.362585892	0.33003059
A_24_P119577	NM_001048183	PHACTR4	8.813043358	10.17532192	8.74596951	10.14732704	1.362278562	1.401357531
A_33_P3340105	NR_033774	BASP1P1	7.332931732	8.694242648	7.625107451	8.138068663	1.361310916	0.512961212
A_33_P3367565		FNBP1	12.60477434	13.96565606	12.00331498	11.89983656	1.360881713	-0.10347842
A_33_P3303577	NM_198563	TMEM110	9.926305082	11.28372351	8.993050628	9.411544896	1.357418424	0.418494268
A_33_P3322288	NM_001134433	AZI2	8.176365224	9.532609226	7.433040917	8.910616237	1.356244002	1.47757532
A_24_P29686	NM_031449	ZMIZ2	11.29197007	12.64402727	9.349730973	10.74531826	1.352057198	1.395587287
A_33_P3314500	NM_005961	MUC6	5.797234795	7.148223046	5.837104184	6.932180221	1.350988251	1.095076037
A_23_P258814	NM_080750	DPH3P1	9.761553711	11.11033418	9.604468526	11.17316072	1.348780466	1.568692198
A_23_P403398	NR_002186	DKFZP586I1420	8.538896141	9.886204203	8.38935128	9.27375351	1.347308062	0.88440223
A_33_P3338166	NM_030952	NUAK2	9.692175977	11.03934181	10.60886549	7.866043537	1.347165829	-2.74282195
A_33_P3238315	NM_006462	RBCK1	9.627722008	10.97482713	9.759871975	9.892308109	1.347105119	0.132436134
A_32_P193080	NM_052905	FMNL2	8.469381035	9.816300057	7.493558777	7.471151718	1.346919022	-0.022407059
A_33_P3304252	NM_031903	MRPL32	10.58506601	11.93086504	11.04139328	10.92980205	1.34579903	-0.111591233
A_33_P3332744	NM_004762	CYTH1	8.686430617	10.02856999	9.069867437	8.801809158	1.342139373	-0.268058279
A_24_P133905	NM_005064	CCL23	11.54061849	12.88260171	7.296517342	10.35051406	1.341983216	3.053996715
A_23_P62840	NM_024640	YRDC	10.88874124	12.23069073	11.33712568	13.40419095	1.341949493	2.067065267
A_24_P169343	NM_153698	C9orf21	9.471600585	10.81320905	9.480963572	10.20024973	1.341608461	0.719286156
A_24_P269432	NM_005868	BET1	8.530121886	9.87141696	9.074309944	9.112364823	1.341295075	0.038054878
A_23_P210176	NM_000210	ITGA6	6.322210087	7.6622262	7.000503564	10.48056816	1.340016112	3.480064599
A_24_P18621	NM_153207	AEBP2	9.062666575	10.40065213	8.245819699	8.922226785	1.337985555	0.676407086
A_33_P3227320	NM_152622	MIER3	9.295343914	10.63319697	8.47254278	9.080103391	1.337853056	0.607560611
A_23_P142407	NM_033204	ZNF101	9.567137556	10.90321771	9.470260114	9.308976861	1.336080157	-0.161283253
A_33_P3276678	NM_199425	VSX1	5.944351609	7.279661236	6.04168981	6.975413655	1.335309627	0.933723845
A_24_P217365	NM_015199	ANKRD28	6.657274904	7.991633006	7.870600616	8.528198648	1.334358101	0.657598032
A_19_P00812723		XLOC_012885	7.294695049	8.627985743	6.846038558	7.825761735	1.333290694	0.979723177
A_24_P385611	NM_003113	SP100	9.070142546	10.40160225	9.5759806	9.663661438	1.331459707	0.087680838
A_33_P3413840	NM_001205019	GK	7.641324967	8.971539911	9.64955725	10.34545837	1.330214944	0.695901123
A_33_P3248329	NM_021943	ZFAND3	8.393803752	9.722349213	8.573946378	9.00986107	1.328545461	0.435914692
A_23_P102037	NM_025147	COQ10B	7.015880106	8.340161704	7.739204045	7.993081761	1.324281599	0.253877715
A_33_P3227731	XR_132557	LOC100506390	11.26168639	12.58530718	10.59658716	11.69202546	1.323620789	1.095438293
A_23_P140301	NM_002788	PSMA3	12.89006573	14.21328352	12.64380011	13.44587525	1.323217783	0.802075137
A_33_P3210827	NM_002878	RAD51D	6.81779153	8.137969065	6.457594584	7.695588719	1.320177535	1.237994135
A_23_P259141	NM_030776	ZBP1	5.496372425	6.816337677	5.731869315	6.223626905	1.319965252	0.49175759
A_23_P364517	NM_021237	SELK	13.34160382	14.66071824	12.86154958	13.27754272	1.319114422	0.415993143
A_23_P116435	NR_028044	IGF2-AS	7.47103027	8.788361375	7.368921849	9.002854821	1.317331105	1.633932972
A_23_P29365	NM_012234	RYBP	8.546212258	9.86347038	8.575319	10.01278136	1.317258122	1.437462355
A_24_P38081	NM_004117	FKBP5	9.029502445	10.34651175	9.034799552	9.299802974	1.317009301	0.265003422
A_33_P3356325	NM_001987	ETV6	7.788070293	9.104534285	8.516836633	8.249482764	1.316463992	-0.267353869
A_24_P181055	NM_006278	ST3GAL4	8.195859349	9.511142998	9.24779371	10.41750793	1.315283649	1.169714217
A_33_P3416231	NM_152739	HOXA9	9.038321542	10.35130805	8.347106957	9.339277387	1.312986504	0.99217043
A_23_P59099	NM_013937	OR11A1	8.314184668	9.625523947	8.403389066	8.611196534	1.311339279	0.207807469
A_24_P418637	NM_012090	MACF1	7.361421713	8.672711875	7.273412794	7.525718935	1.311290162	0.252306142
A_23_P133345	NM_014666	CLINT1	10.70490439	12.01535835	10.6490941	11.2577456	1.310453962	0.608651497
A_23_P122579	NM_001350	DAXX	10.1659327	11.47632166	10.47715692	11.27793211	1.310388959	0.800775192
A_23_P154235	NM_004688	NMI	11.19367544	12.50261748	10.58333673	11.42589328	1.308942044	0.842556543
A_23_P76109	NM_145058	RILPL2	12.05235699	13.36098165	13.18690418	13.17893473	1.308624658	-0.007969443
A_24_P348925	NM_001099402	CCNK	9.305832974	10.6134629	9.209756803	9.774640511	1.307629929	0.564883708
A_23_P131208	NM_006186	NR4A2	6.429408629	7.733647407	6.220915507	6.619577049	1.304238778	0.398661542
A_24_P345209	NM_001004023	DYRK3	5.399104174	6.700274095	5.989140345	8.116602532	1.301169922	2.127462187
A_23_P41267	NR_026854	LOC401127	7.528249548	8.827939508	8.233328766	9.036028603	1.29968996	0.802699837
A_24_P280833	NR_033990	LOC100129138	8.227922907	9.524639329	8.415278101	8.749370279	1.296716422	0.334092178
A_23_P397391	NM_005306	FFAR2	5.985329326	7.277084405	6.062034839	8.213343095	1.291755079	2.151308256
A_24_P89872	NM_197977	ZNF189	9.589623203	10.88008163	8.474732008	10.09785257	1.290458423	1.623120563
A_33_P3313617	NR_003133	GBP1P1	4.9829483	6.272371913	5.402084553	5.549205972	1.289423613	0.147121419
A_19_P00321577		XLOC_010390	7.220719676	8.509344675	7.816792605	9.816470463	1.288624999	1.999677858
A_33_P3380493	NM_031286	SH3BGRL3	7.181976423	8.470276033	7.265251451	7.315304745	1.28829961	0.050053294
A_23_P360964	NM_145056	DACT3	6.974038717	8.260820358	6.633326164	6.408024331	1.286781641	-0.225301833
A_24_P172481	NM_006074	TRIM22	10.64243622	11.92649244	11.03328549	11.33712614	1.284056221	0.303840643
A_23_P381714	NM_198584	CA13	6.416224035	7.700027435	5.541496935	6.873435262	1.283803401	1.331938327
A_33_P3325257	NR_024496	LOC442421	6.312620121	7.590695102	6.812256691	6.440181683	1.278074981	-0.372075008
A_33_P3341189	NM_003336	UBE2A	11.0723465	12.3498257	11.22471544	11.54140791	1.277479199	0.31669247
A_24_P942481	NM_180989	GPR180	7.336359747	8.613112533	7.149551725	7.958871171	1.276752786	0.809319446
A_32_P69368	NM_002166	ID2	12.85712147	14.13336026	13.43821067	13.06826222	1.276238791	-0.36994845
A_33_P3338693	NM_003081	SNAP25	6.770318211	8.044607692	7.230428843	8.860436744	1.274289481	1.630007901
A_19_P00322096		XLOC_005285	9.171574748	10.44501943	9.110332357	9.454096347	1.273444682	0.34376399
A_33_P3316522	NM_001037500	DEFB124	6.933191215	8.199959147	7.141295296	7.697248358	1.266767932	0.555953062
A_33_P3867584	NR_038863	LOC285758	5.789018052	7.054493514	5.754188475	6.016656545	1.265475462	0.26246807
A_23_P218807	NM_017590	ZC3H7B	9.637123169	10.90187743	9.178295802	9.136760897	1.264754257	-0.041534905
A_33_P3229863	NR_040082	LOC100128714	11.47745279	12.7408582	10.81894353	11.87282394	1.263405417	1.05388041
A_23_P258944	NM_012328	DNAJB9	10.91173785	12.17488538	11.44503513	12.09035892	1.263147529	0.645323793
A_23_P254079	NM_003943	STBD1	6.238773656	7.498700843	6.473183501	8.110827822	1.259927187	1.637644321
A_33_P3388983		C5orf56	5.582413111	6.840389692	5.825761638	6.040408242	1.257976581	0.214646604
A_23_P37068	NM_145725	TRAF3	7.572348762	8.828472434	7.578611567	8.534392025	1.256123672	0.955780459
A_23_P78750	NM_020309	SLC17A7	8.805440886	10.0608777	7.917494688	9.184558631	1.255436814	1.267063943
A_23_P45699	NM_003902	FUBP1	9.025936447	10.27912796	8.508367763	9.322585281	1.253191517	0.814217518
A_23_P30126	NM_005130	FGFBP1	5.36868025	6.619442191	5.482524689	6.425353078	1.250761941	0.942828389
A_23_P55990	NM_003827	NAPA	9.159040047	10.40698169	9.142562583	9.455137883	1.247941639	0.3125753
A_24_P282309	NM_133337	MYOF	7.399806756	8.645963808	9.376356899	9.494354246	1.246157052	0.117997347
A_23_P65442	NM_006084	IRF9	9.946168984	11.19204025	9.733172567	10.77399041	1.245871266	1.040817843
A_23_P161624	NM_005438	FOSL1	6.437645421	7.683139301	7.114866649	7.772971073	1.245493881	0.658104424
A_23_P90419	NM_025245	PBX4	6.141957173	7.381678591	6.416479395	6.893875077	1.239721418	0.477395682
A_24_P232790	NR_024630	C14orf162	7.620916893	8.859881961	7.049455794	8.261009953	1.238965068	1.21155416
A_23_P151209	NM_030809	CSRNP2	9.115406789	10.35374034	9.254066046	10.54063497	1.238333555	1.286568927
A_23_P31389	NM_013293	TRA2A	7.26713808	8.503709677	7.191734543	6.743090333	1.236571598	-0.44864421
A_24_P342632	NM_174858	AK5	5.280678769	6.515756233	5.263291703	6.336447088	1.235077464	1.073155385
A_23_P79818	NM_016470	C20orf111	10.67134313	11.9058922	11.4923108	11.01384862	1.234549072	-0.47846218
A_23_P147025	NM_004794	RAB33A	11.27907123	12.51302599	9.318836458	12.11271981	1.23395476	2.793883352
A_23_P134109	NM_001431	EPB41L2	9.142887326	10.37683368	9.81263076	9.678738736	1.233946354	-0.133892024
A_33_P3248749		RSPH9	11.52342238	12.75718067	11.39946693	12.5887721	1.233758291	1.189305173
A_33_P3385656	NM_001001343	FNDC9	7.271806613	8.504787965	7.385092148	7.802762673	1.232981351	0.417670525
A_33_P3333337	NM_001164213	LRCH1	6.860063295	8.092943127	7.545778321	7.909043126	1.232879833	0.363264805
A_33_P3213468	NM_001164440	ANKRD33B	6.110496346	7.342397902	5.948144779	6.121308598	1.231901556	0.173163819
A_23_P345710	NM_152531	XXYLT1	8.294393135	9.524377105	8.173785228	8.17974676	1.22998397	0.005961532
A_33_P3232557	NM_001080418	DLGAP3	7.179976312	8.409386975	10.05710359	10.21194286	1.229410663	0.154839274
A_23_P23728	NM_014849	SV2A	7.670682542	8.897622807	7.292410548	7.982706084	1.226940265	0.690295536
A_23_P138975	NM_016090	RBM7	8.694737656	9.921102123	8.613904423	9.777417703	1.226364467	1.16351328
A_32_P139894	NM_007314	ABL2	10.52699742	11.75141806	10.0333001	11.67089184	1.224420644	1.637591738
A_33_P3360426	NM_017491	WDR1	8.411339122	9.630164696	8.748473265	8.730572251	1.218825573	-0.017901014
A_23_P14174	NM_006573	TNFSF13B	12.65787097	13.87469139	11.68320196	12.22555589	1.216820419	0.542353927
A_24_P270814	NM_016823	CRK	7.540539845	8.755963042	7.809427781	8.475654748	1.215423197	0.666226968

A_33_P3410859	NR_003288	LOC729603	6.321291188	7.535272174	7.770513487	8.482913805	1.213980987	0.712400318
A_33_P3398862	NM_004040	RHOB	7.882706742	9.094487934	8.406981789	8.154334717	1.211781192	-0.252647072
A_23_P55706	NM_006509	RELB	13.52712049	14.73748816	12.03053781	14.08747445	1.210367671	2.05693664
A_23_P201808	NM_003713	PPAP2B	10.76104358	11.97133658	10.74978241	12.47711336	1.210293001	1.72733095
A_23_P423389	NM_006368	CREB3	9.087619119	10.29661088	9.372453596	9.968328627	1.208991764	0.595875031
A_23_P167595	NM_003337	UBE2B	8.328706917	9.534004128	8.214170565	8.472263585	1.205297211	0.25809302
A_33_P3367062	NM_017673	SWT1	6.8180522	8.021431867	6.652671455	7.665557267	1.203379667	1.012885812
A_23_P35148	NM_005645	TAF13	7.629724018	8.830436779	7.684755838	7.927620097	1.200712761	0.24286426
A_23_P51699	NM_004723	ARHGEF2	8.921313348	10.12112675	9.759667491	10.60352242	1.199813403	0.843854929
A_33_P3231187	NM_001167	XIAP	6.839130474	8.037166142	7.239478326	7.792798212	1.198035668	0.553319886
A_33_P3260445		DYNLT1	6.052959396	7.249340404	6.490462929	7.204062489	1.196381007	0.71359956
A_23_P111804	NM_022750	PARP12	11.30341691	12.49943421	10.28868857	10.74020369	1.196017293	0.451515128
A_23_P48803	NM_014548	TMOD2	6.11502231	7.310560217	5.399020879	6.496530995	1.195537906	1.097510116
A_23_P258164	NM_001302	CORT	7.800818971	8.993750517	7.464814362	8.34449629	1.192931546	0.879681928
A_33_P3317797	NM_001135585	SLC2A5	6.014094903	7.205390683	6.286548494	6.959308736	1.19129578	0.672760242
A_33_P3377209	NM_207168	ENSA	7.888362878	9.079574164	8.244840609	9.128002458	1.191211286	0.883161848
A_33_P3226985	NM_003885	CDK5R1	6.990934684	8.180833712	7.040711825	7.566324915	1.189899028	0.525613091
A_23_P140035	NM_007187	WBP4	10.19814579	11.38696006	9.5921629	10.73163551	1.188814277	1.139472606
A_33_P3297050	NM_033087	ALG2	8.198704785	9.386455324	7.454840392	8.602305744	1.187750539	1.147465352
A_23_P171054	NM_005229	ELK1	6.125589549	7.310521415	7.161997379	6.843724323	1.184931866	-0.318273056
A_24_P73389	NM_001032296	STK24	7.647644115	8.831502187	8.606573673	7.663501597	1.183858071	-0.943072076
A_23_P306655	NM_198722	AMIGO3	8.134833404	9.31580866	7.472477637	7.845923425	1.180975256	0.373445788
A_23_P97394	NM_003567	BCAR3	10.68807021	11.86859725	9.057623107	11.35903327	1.180527038	2.301410163
A_24_P243776	NM_021994	ZNF277	9.742991159	10.92223115	9.695350152	9.725654831	1.179239991	0.030304679
A_24_P350245	NM_024940	DOCK5	7.546523157	8.724749123	8.313780812	9.642462762	1.178225966	1.32868195
A_23_P304279	NM_000080	CHRNE	6.819145025	7.99709361	7.031376106	7.237401609	1.177948585	0.206025503
A_23_P154938	NM_003325	HIRA	9.784738443	10.96254746	9.247054501	9.558287165	1.177809014	0.311232664
A_19_P00322813		XLOC_007290	7.586508098	8.763847252	7.656634739	8.606914466	1.177339154	0.950279727
A_33_P3283196		SCAF11	7.61072375	8.787715204	7.567565939	9.701539986	1.176991454	2.133974047
A_23_P67829	NM_025076	UXS1	9.064346739	10.2409042	9.492928879	11.53505031	1.176557465	2.042121434
A_19_P00804922		XLOC_003641	6.155274907	7.330301374	6.084908931	6.34543264	1.175026467	0.260523709
A_24_P174341	NM_058241	CCNT2	6.618468604	7.788732261	6.787218571	7.415932807	1.170263657	0.628714235
A_23_P340890	NM_001195753	THAP3	9.917965603	11.08567364	10.29098974	10.27214114	1.167708037	-0.018848597
A_23_P416191	NM_176885	TAS2R31	6.629790451	7.795946591	7.073646231	6.715971813	1.166156141	-0.357674418
A_23_P364766	NM_001009608	C20orf94	8.772584811	9.937786381	8.310510262	9.386447926	1.16520157	1.075937664
A_23_P42241	NM_030876	OR5V1	7.48116813	8.645196777	7.001842089	7.955603037	1.164028647	0.953760948
A_23_P932016	NM_031407	HUWE1	7.127542652	8.291060967	7.108037994	7.499218859	1.163518314	0.391180865
A_24_P287473	NM_021818	SAV1	10.28700402	11.45030561	11.17737857	11.49050504	1.163301593	0.31312647
A_23_P119362	NM_001425	EMP3	13.32805426	14.48996713	13.18353922	13.51213024	1.161912867	0.328591017
A_24_P944253	NM_130446	KLHL6	8.878670721	10.03830704	9.80939934	9.941470971	1.159636315	0.132071631
A_33_P3613000	NM_001105539	ZBTB10	6.16682398	7.325809147	5.775604595	6.052088443	1.158985167	0.276483847
A_33_P3398091	NM_001130031	ZNF562	9.209163497	10.36723393	8.725467989	10.01342348	1.158070429	1.287955487
A_23_P166248	NM_004414	RCAN1	10.16488071	11.32276988	8.687904078	11.03114207	1.15788917	2.343237989
A_23_P201951	NM_016374	ARID4B	8.646525592	9.803272828	8.625547822	8.893473301	1.156747236	0.267925479
A_33_P3405957	NM_182557	BCL9L	6.653250482	7.80946976	7.157795946	8.932335518	1.156219278	1.774539572
A_33_P3332474	NM_001001413	GOLGA6L1	6.097306505	7.253167045	6.136157375	6.995336881	1.155860539	0.859179505
A_23_P21838	NM_033133	CNP	13.53985203	14.69556459	13.00191438	13.3518897	1.155712564	0.34997532
A_23_P72915	NM_133496	SLC30A7	7.273524938	8.427694839	7.53693403	7.96196671	1.154169902	0.42503268
A_33_P3358957	NM_001004318	PAPL	9.279800479	10.43396115	9.158788389	9.239045343	1.154160671	0.080256955
A_24_P136161	NM_001013631	HNRNPCL1	6.659045742	7.811561701	7.06239754	8.126144862	1.152515958	1.063747322
A_23_P203173	NM_001558	IL10RA	14.5588787	15.71050726	14.31712255	15.22561166	1.15162856	0.908489107
A_23_P148273	NM_032121	MAGT1	9.026651572	10.17765727	8.695602932	9.369612571	1.1510057	0.674009639
A_23_P306479	NR_024407	LOC100009676	7.074288093	8.224670383	6.507528251	7.391707948	1.15038229	0.884179697
A_24_P218265	NM_003842	TNFRSF10B	12.0994425	13.24667315	12.09993024	12.3236247	1.147230647	0.223694457
A_33_P3339231	NM_173569	UBN2	8.072697885	9.219311261	8.069554754	7.44256508	1.146613376	-0.626989675
A_23_P362659	NM_002468	MYD88	11.0891423	12.2356218	11.83898377	12.25140257	1.146479499	0.4124188
A_33_P3411885		LOC100128851	8.629720628	9.776018282	9.110702889	9.556591946	1.146297655	0.445889058
A_33_P3372124	NM_001143906	TRAFD1	12.8468823	13.99037072	12.62995821	12.38283259	1.143488422	-0.24712562
A_33_P3378785		FAM99A	8.319736372	9.461285817	8.542667973	9.146934776	1.141549445	0.604266803
A_32_P201773	NM_015365	AMMECR1	7.258493094	8.399398198	6.504522478	6.495773836	1.140905104	-0.008748642
A_23_P7423	NM_017755	NSUN2	10.95123625	12.09115613	10.85298392	11.657941	1.139919879	0.804957073
A_24_P11791	NM_002268	KPNA4	11.63041553	12.76983823	12.00453158	12.73144872	1.139422895	0.726917143
A_33_P3345796		SNX3	5.733227549	6.872189758	6.366060263	6.552025848	1.13896221	0.185965585
A_33_P3369969	NM_001137675	ATXN1L	9.278966507	10.41760534	8.701009458	9.335303351	1.13863883	0.634293893
A_23_P13683	NM_014830	ZBTB39	6.170494943	7.308415276	6.106974683	6.58517444	1.137920333	0.478199758
A_23_P121564	NM_000857	GUCY1B3	5.589481502	6.727270595	5.749397	6.842834582	1.137789093	1.093437582
A_33_P3279545	NM_005734	HIPK3	7.170086732	8.307652047	7.482569008	7.984148708	1.137565315	0.5015797
A_33_P3294986	NM_005357	LIPE	7.686153995	8.822750457	7.368963219	7.955190236	1.136596462	0.586227016
A_33_P3291732	XR_111839	FAM125A	6.961106148	8.096779247	7.140397156	7.64494301	1.135673099	0.504545854
A_23_P327022	NM_199072	MDFIC	10.06924191	11.2027392	10.07280165	10.17031066	1.133497295	0.09750901
A_24_P340066	NM_001421	ELF4	11.84920686	12.98238612	13.03214369	13.38482617	1.133179258	0.352682487
A_23_P58983	NM_017772	TBC1D22B	7.411798979	8.542531397	7.82654591	8.835624021	1.130732419	1.009078111
A_23_P350234	NM_001012989	UBE2NL	8.718839111	9.847348183	8.494275158	9.633839154	1.128509072	1.139563996
A_23_P40295	NM_012261	C20orf103	5.967285273	7.094370271	5.65850642	5.983173308	1.127084998	0.324666888
A_23_P211806	NM_017724	LRRFIP2	9.560627664	10.68025857	9.185760439	10.64630726	1.11963091	1.460546825
A_23_P80827	NM_001011537	FYTTD1	10.57445897	11.6926862	10.30447355	11.08238104	1.118227226	0.777907483
A_32_P45168	NM_002184	IL6ST	8.916552401	10.03394335	8.627785268	9.710254301	1.117390948	1.082469034
A_33_P3267186	NM_001682	ATP2B1	9.145168293	10.26230666	7.842454699	10.97099268	1.117138371	3.128537978
A_33_P3396159	NM_005436	CCDC6	8.694443648	9.81079133	9.509721439	9.152824222	1.116347682	-0.356897217
A_23_P135669	NM_030958	SLCO5A1	6.446634494	7.562715241	5.550202639	5.97108363	1.116080746	0.420880991
A_23_P3204	NM_002748	MAPK6	10.69434661	11.81030735	10.88949554	11.83260094	1.115960739	0.9431054
A_24_P381494	NM_000617	SLC11A2	9.618290742	10.73291597	9.270569222	11.6822299	1.114625231	2.411660675
A_23_P259580	NM_172208	TAPBP	8.162766739	9.276947849	8.235789786	8.523146387	1.11418111	0.287356601
A_23_P215675	NM_018224	C7orf44	9.715137022	10.82920711	10.24994418	10.45039398	1.114070088	0.2004498
A_23_P216766	NM_030940	ISCA1	7.02624702	8.139769243	7.749979854	7.612628258	1.113522223	-0.137351597
A_23_P151497	NM_152307	TRMT61A	7.870193399	8.983252888	7.600657649	8.636834363	1.113059489	1.036176714
A_24_P9671	NM_001539	DNAJA1	12.80102413	13.91239477	12.25503539	12.98195162	1.111370633	0.726916237
A_33_P3321136	NM_014957	DENND3	6.654906921	7.766245292	7.286465016	7.583432473	1.111338371	0.296967457
A_33_P3421571	NM_213589	RAPH1	10.68080943	11.79206174	10.41607031	11.0140822	1.111252312	0.598011897
A_24_P62783	NM_004102	FABP3	11.00477092	12.11577844	11.01505774	10.21863339	1.111007515	-0.796424348
A_23_P373119	NR_002165	HMGB3P1	7.928381337	9.036931535	7.796939631	9.075848928	1.108550198	1.278909297
A_24_P97526	NM_017801	CMTM6	12.993807	14.10165764	12.06993195	12.87450606	1.10785064	0.804574107
A_23_P50646	NM_001193621	LOC390940	7.387285846	8.494398461	8.672224387	8.519492862	1.107112615	-0.152731525
A_23_P154025	NM_003142	SSB	12.61247553	13.71958118	11.85895282	12.29914234	1.107105657	0.440189523
A_33_P3216297	NM_001018077	NR3C1	9.750248256	10.85595393	9.307968307	9.924831781	1.105705678	0.616863474
A_33_P3370364	NM_004248	PRLHR	9.472819198	10.57806286	8.978663791	9.629542004	1.105243662	0.650878213
A_23_P218505	NM_000894	LHB	8.528697652	9.633529283	8.65836346	9.385255898	1.104831631	0.726892438
A_24_P152468	XM_001718823	LOC100134663	9.968396444	11.07305668	9.987972523	10.56036645	1.104660239	0.572393927
A_24_P194714	NM_182565	FAM100B	12.7039789	13.80811718	13.41232994	14.19661723	1.104138283	0.784287293
A_33_P3428642	NM_004943	DMWD	7.869894311	8.97387417	7.402191903	7.774411097	1.103979858	0.372219194
A_23_P137586	NM_006582	GMEB1	8.912185965	10.01325128	8.962173198	10.28171436	1.101065314	1.319541165

A_33_P3247082	NR_003083	SLC6A10P	9.466730327	10.56640444	9.281848253	9.995695177	1.099674109	0.713846924
A_33_P3213169		ITGAX	8.575484173	9.672175413	9.906509548	10.038389	1.09669124	0.131879452
A_24_P548866	NM_001099668	HIGD1A	6.634891199	7.731336001	6.424870502	7.269256629	1.096444802	0.844386127
A_33_P3327852	NM_030808	NDEL1	6.592351995	7.688514915	7.261359993	7.727170894	1.09616292	0.4658109
A_33_P3294404	NM_024595	AKIRIN1	8.808609208	9.904470357	9.282844192	10.30012763	1.095861148	1.017283438
A_33_P3239347	NM_006167	NKX3-1	5.702492042	6.795946394	5.892262957	7.472287907	1.093454352	1.58002495
A_33_P3421695	NM_001098210	CTNNB1	8.445990352	9.53914048	8.49017365	8.42297554	1.093150128	-0.067198111
A_32_P148345	NM_001002857	ANXA2	9.182118507	10.2730127	9.786835238	9.979625533	1.090894193	0.192790295
A_24_P286465	NM_033224	PURB	10.56096532	11.65095537	10.08003495	11.27134163	1.089990055	1.191306679
A_33_P3342957	NM_004226	STK17B	9.76803341	10.85727009	9.319118246	8.40263624	1.08923668	-0.916482005
A_23_P69188	NM_206831	DPH3	11.17800545	12.26715931	11.25456249	12.09099823	1.089153864	0.836435743
A_24_P342096	NR_024060	FAM27A	8.171893406	9.260941216	8.808353742	8.364135307	1.089047811	-0.444218435
A_23_P366328	NM_152415	VPS37A	9.967479333	11.05429779	10.78348228	11.3382307	1.086818453	0.554748417
A_24_P942370	NM_003774	GALNT4	5.908800143	6.994538096	5.648993232	6.475159342	1.085737952	0.82616611
A_33_P3303519	NM_205852	CLEC12B	7.639167433	8.72407651	7.759038027	7.891474931	1.084909077	0.132436904
A_33_P3460043	NR_027071	C8orf56	10.77101697	11.85532161	10.96441405	10.83765655	1.084304639	-0.1267575
A_23_P135778	NM_018461	PPP2R2D	9.621869527	10.70596527	10.10799387	10.37731204	1.084095746	0.26931817
A_33_P3270317	NM_003055	SLC18A3	8.365120485	9.446717354	8.049676059	8.532331692	1.081596868	0.482655632
A_33_P3389967		USP49	6.906640505	7.985333778	7.260777473	8.017225498	1.078693273	0.756448025
A_33_P3234317	NM_012250	RRAS2	5.740107241	6.818263629	5.876908533	6.162423381	1.078156388	0.285514848
A_23_P380298	NM_014731	ProSAPiP1	5.9651416	7.041634827	6.194598966	6.740865745	1.076493228	0.546266779
A_23_P92642	NM_024668	ANKHD1	9.209925655	10.28582054	10.00970151	10.24682413	1.075894885	0.237122621
A_23_P28169	NM_152522	ARL6IP6	9.949310638	11.02511541	8.71690274	10.12409896	1.075804772	1.407196216
A_33_P3349651	NM_015055	SWAP70	10.29849011	11.37397835	8.696869137	9.703118144	1.075488236	1.006249007
A_32_P96807	NM_172071	RC3H1	10.09207863	11.16735579	9.603896465	11.20915307	1.075277159	1.605256605
A_33_P3390177	NR_033929	FLJ34208	6.181592576	7.256301462	6.363805741	6.518216958	1.074708886	0.154411217
A_23_P379026	NM_019096	GTPBP2	10.49667677	11.56603586	10.92369377	12.04980815	1.069359091	1.126114377
A_24_P137376	NM_001001485	ATP2C1	9.118740259	10.18695741	9.247691065	10.03624244	1.068217154	0.788551375
A_23_P500364	NM_001707	BCL7B	8.333966424	9.400835582	8.623297205	9.135647121	1.066869158	0.512349917
A_33_P3337771	NM_024831	TGS1	9.751760824	10.81850408	9.38450828	9.96846087	1.066743256	0.583952589
A_23_P127579	NM_000317	PTS	9.969483787	11.0361806	10.61436762	11.78605638	1.066696816	1.171688767
A_32_P128391	NR_038842	LOC728431	8.302867108	9.36927235	8.944275667	9.632262374	1.066405243	0.687986707
A_23_P407684	NM_178167	ZNF598	8.787444176	9.852240236	8.882941317	9.986074401	1.064796061	1.103133084
A_23_P203743	NM_012296	GAB2	11.00170143	12.06545176	11.44758068	11.66715114	1.063750328	0.219570453
A_23_P391764	NM_198679	RAPGEF1	7.574847159	8.6377323	7.806108476	7.701302137	1.06288514	-0.104806339
A_23_P71148	NM_000712	BLVRA	15.01233127	16.07425588	14.57049732	14.92686293	1.061924617	0.35636561
A_33_P3215288		LOC284757	6.741634513	7.799834874	6.765722548	7.192386527	1.058200362	0.42666398
A_23_P11279	NM_018466	ALG13	9.912157176	10.96931877	8.861858582	10.18110023	1.057161594	1.319241648
A_24_P201739	NM_005475	SH2B3	10.73541409	11.79175192	9.920110738	11.57417116	1.056337834	1.654060419
A_33_P3210647	NM_001856	COL16A1	8.937259331	9.993550692	8.288563354	8.84036916	1.056291361	0.551805805
A_23_P51679	NM_005920	MEF2D	10.75994135	11.81510664	10.28469924	11.01328767	1.055165285	0.728588437
A_33_P3370930		LAMB1	8.097874446	9.152890718	7.85444222	8.308509172	1.055016272	0.454066951
A_33_P3238410	NM_002972	SBF1	6.691898014	7.746790774	7.029243843	7.064003452	1.054892761	0.034759608
A_24_P116535	NM_002428	MMP15	7.974940206	9.029512869	7.284069709	7.625866557	1.054572663	0.341796848
A_23_P408675	NM_003972	BTAF1	8.108487753	9.162511821	8.569932457	9.131950187	1.054024068	0.56201773
A_23_P163195	NM_152447	LRFN5	5.273338165	6.327055401	5.225763714	6.175897412	1.053717236	0.950133698
A_33_P3263851	XR_109667	FLJ46020	8.08784071	9.14132125	7.423990588	8.386087108	1.05348054	0.962096521
A_24_P911607	NM_058238	WNT7B	5.383361298	6.436276725	5.458104952	6.138765326	1.052915427	0.680660374
A_23_P304543	NM_147133	NFX1	9.403002917	10.45511039	9.447704012	9.768353538	1.052107473	0.320649526
A_23_P47991	NM_015335	MED13L	9.614586106	10.66557976	8.946092917	10.12358948	1.050993657	1.17749656
A_33_P3462960	NM_006260	DNAJC3	10.37394592	11.42466407	10.0645104	11.11150351	1.050718155	1.046993114
A_23_P91221	NM_181805	PKIG	6.24471666	7.29518909	7.246760933	8.819910577	1.05047243	1.573149645
A_23_P167559	NM_144726	RNF145	10.3459791	11.39614814	10.38556596	11.09859213	1.050169042	0.713026173
A_23_P311616	NM_015167	JMJD6	9.066865007	10.1169173	9.389162536	10.07249843	1.050052296	0.683335899
A_33_P3629247	NM_017844	ANKMY1	6.483025716	7.531789579	8.022952425	7.02018348	1.048763863	-1.002768946
A_24_P318656	NM_000212	ITGB3	5.770154641	6.818562033	5.803503702	7.825766648	1.048407392	2.022262945
A_23_P8664	NM_021145	DMTF1	9.049454477	10.09775297	8.742214502	10.0317043	1.048298495	1.289489793
A_24_P262738	NM_024050	DDA1	8.670717208	9.717287641	8.9780275	9.606527778	1.046570433	0.628500278
A_23_P328836	NM_032440	LCOR	6.618230966	7.664072442	6.902720989	8.165223997	1.045841477	1.262503007
A_23_P66017	NM_145239	PRRT2	6.087051847	7.13071799	6.51494897	6.314167568	1.043666143	-0.200781402
A_33_P3397603		LOC100130278	5.837429295	6.879895215	6.173596654	5.86965949	1.04246592	-0.303937164
A_23_P42198	NM_003534	HIST1H3G	10.4687849	11.51041225	10.30026153	10.94861585	1.041627346	0.648354316
A_24_P132470	NM_020728	ESYT2	9.878140837	10.91924483	10.04697348	10.19112347	1.041103997	0.144149985
A_23_P255653	NM_003844	TNFRSF10A	7.662943511	8.702656623	8.241423098	8.818394388	1.039713112	0.57697129
A_32_P192474	NM_030651	PRRT1	7.03250241	8.071428627	7.036618709	8.115192549	1.038926217	1.078573841
A_32_P19134	NM_032119	GPR98	5.522278044	6.558865411	5.685641068	6.23124362	1.036587367	0.545602552
A_23_P3221	NM_021199	SQRDL	15.27770627	16.31308844	15.28090589	15.90854025	1.035382172	0.627634353
A_23_P151307	NM_006105	RAPGEF3	5.729885195	6.765095609	7.015588555	6.190744163	1.035210415	-0.824844392
A_24_P237753	NM_015354	NUP188	7.562095451	8.595946265	8.198346058	8.250678103	1.033850814	0.052332045
A_33_P3334037	NM_016312	WBP11	9.619609385	10.65184131	10.5261748	10.419333	1.032231922	-0.106841793
A_33_P3317282	NM_138459	NUS1	7.879833986	8.912064056	8.95332276	9.707430487	1.03223007	0.754107727
A_33_P3832857	NM_018097	HAUS2	8.022228758	9.052336748	8.43169576	9.351709291	1.03010799	0.920013531
A_23_P135857	NM_004836	EIF2AK3	7.28869566	8.318157046	7.405770827	8.955761584	1.029461385	1.549990757
A_24_P276531	NM_024700	SNIP1	7.138701679	8.166897129	7.094466084	7.681169998	1.02819545	0.586703914
A_33_P3278774		ZNF44	6.693781605	7.720612601	6.423808935	7.927848909	1.026830996	1.504039974
A_23_P201596	NM_004037	AMPD2	9.273325677	10.29939692	11.28332645	10.99544245	1.026071243	-0.287884003
A_24_P84880	NR_002929	LOC148709	10.18365947	11.20966883	10.04248517	11.10267373	1.02600936	1.060188562
A_23_P65208	NM_001039650	ZMYM5	7.202206329	8.227686237	7.612389021	8.043904159	1.025479908	0.431515138
A_33_P3382746	NM_005356	LCK	8.76470189	9.788831661	11.05348298	11.15154772	1.024129771	0.098064747
A_23_P107454	NM_031958	KRTAP3-1	6.34769432	7.371519663	6.32447618	7.548402315	1.023825343	1.223926135
A_32_P160896	NM_177478	FTMT	7.307324078	8.330173243	7.822427677	8.462750273	1.022849166	0.640322597
A_24_P130792	NM_199044	NSUN4	7.252280213	8.274938457	7.477983422	7.720348297	1.022658244	0.242364876
A_33_P3287119	NM_001122646	FAM178B	9.006458714	10.02908111	8.843461686	9.585519579	1.022622391	0.742057893
A_33_P3405754		CEP104	7.645609545	8.66439351	7.843232466	8.24059368	1.018783966	0.397361214
A_23_P305723	NM_020948	MIER1	9.454436993	10.47186237	9.033097808	10.32598801	1.01742538	1.292890198
A_33_P3629131		CDRT3	7.710846895	8.728170952	7.679080142	8.341133058	1.017324057	0.662052916
A_23_P91414	NM_080625	C20orf160	5.153058659	6.170212204	5.277083386	6.482328464	1.017153546	1.205245078
A_23_P21134	NM_004083	DDIT3	8.390130375	9.407098592	9.627032469	9.097770368	1.016968217	-0.529262101
A_24_P271527	NM_014876	JOSD1	12.32438661	13.33934741	12.03004537	12.72675906	1.014960802	0.696713693
A_33_P3422991	NM_152313	SLC36A4	8.548147913	9.562214414	9.270302832	10.49532675	1.014066501	1.225023922
A_24_P141019	NM_018412	ST7	6.959533276	7.972944765	6.809395368	7.483230442	1.013411489	0.673835074
A_23_P42935	NM_004333	BRAF	7.62082815	8.633438091	7.720210387	8.161265268	1.012609941	0.441054881
A_23_P426809	NM_198236	ARHGEF11	8.409876933	9.422224509	8.43756314	7.869616887	1.012347576	-0.567946253
A_23_P85171	NM_000117	EMD	9.601144638	10.61340221	9.659389009	10.3969876	1.012257569	0.737598594
A_23_P116829	NM_003348	UBE2N	8.135673365	9.147419698	7.80230056	8.674002709	1.011746333	0.871702149
A_23_P428827	NM_018064	AKIRIN2	9.918812701	10.92863025	9.869622292	9.992865766	1.009817552	0.123243474
A_33_P3419334	NM_006496	GNAI3	9.247312443	10.2562134	8.961726027	9.603168623	1.008900957	0.641442596
A_19_P00317411		XLOC_000787	9.911929563	10.92055611	10.52045238	11.0834461	1.008626543	0.562993723
A_33_P3372563	NM_033450	ABCC10	7.567976753	8.574810996	8.022438443	9.208352522	1.006834243	1.185914079
A_33_P3396389	NM_000877	IL1R1	9.595132077	10.60098923	8.789384705	8.98875687	1.005857153	0.199372165
A_33_P3219303	NM_001807	CEL	8.818274798	9.822265115	8.742849922	9.102772055	1.003990317	0.359922133

A_33_P3319491	NM_015878	AZIN1	11.25562578	12.25913922	11.14072801	11.86640366	1.003513441	0.725675653
A_23_P111240	NM_014721	PHACTR2	8.819868034	9.822835419	7.847011266	8.490131488	1.002967385	0.643120222
A_23_P330262	NM_012398	PIP5K1C	8.452968091	9.455514849	8.151102603	8.021624091	1.002546758	-0.129478512
A_23_P46429	NM_001554	CYR61	5.269014372	6.269638245	5.360088515	5.465538259	1.000623873	0.105449744
A_24_P623782	NM_001005217	FRG2	5.645805053	6.646076845	5.596053947	6.505714075	1.000271792	0.909660128
A_23_P11843	NM_201630	LRRN2	8.300375649	9.300506697	7.888000758	8.584734472	1.000131048	0.696733714
A_23_P88278	NM_020366	RPGRIP1	7.445827054	8.43406707	5.65425097	8.371431308	0.988240016	2.717180338
A_24_P91852	NM_006520	DYNLT3	9.525164153	10.51110932	8.414756668	10.32946763	0.985945171	1.914710962
A_33_P3223923	NM_005313	PDIA3	8.183149211	9.167314753	7.84159242	8.966166575	0.984165543	1.124574155
A_23_P25155	NM_020370	GPR84	7.633507613	8.612469146	9.949964212	11.63014901	0.978961533	1.680184802
A_33_P3236798	NM_080823	SRMS	9.568250025	10.52092622	9.358279362	10.40232717	0.952676198	1.044047811
A_33_P3303215	NM_001191009	SRSF10	9.710047486	10.65765358	9.274583108	10.35918263	0.947606098	1.084599525
A_33_P3257503	NR_003930	LOC387647	9.346340454	10.28941705	8.165565943	9.673011519	0.943076592	1.507445577
A_23_P205686	NM_000021	PSEN1	11.46083065	12.40366883	11.18014452	12.28317312	0.942838177	1.103028593
A_33_P3216694	NM_024503	HIVEP3	6.249736791	7.189800702	6.588580446	7.866865923	0.940063911	1.278285478
A_23_P80648	NM_033540	MFN1	8.524914641	9.460744203	8.669120677	9.819117183	0.935829562	1.149996505
A_23_P97573	NM_004698	PRPF3	8.364812023	9.298515379	8.521385109	9.99959352	0.933703356	1.478208411
A_33_P3334102	NM_174919	ARHGAP27	9.329112383	10.26255038	9.025566453	10.37277172	0.933437997	1.34720527
A_33_P3274397	NM_001145414	CHM	6.540293696	7.46169191	6.070549509	7.206109498	0.921398214	1.135559989
A_23_P6786		TRNT1	7.720456996	8.6413013	7.089784509	8.198809119	0.920844305	1.10902461
A_33_P3422133	NM_006869	ADAP1	10.92816981	11.84535483	9.264562277	10.9241433	0.917185018	1.659581019
A_33_P3271490	NM_016836	RBMS1	9.471653125	10.38039518	8.922799171	9.993115	0.908742058	1.070315829
A_33_P3422170	NM_194247	HNRNPA3	7.375846022	8.280506282	7.022883786	8.27841504	0.90466026	1.255531254
A_24_P329924	NM_025164	SIK3	8.553212681	9.4436464	8.402594659	9.564002126	0.890433719	1.161407467
A_23_P404667	NM_001197	BIK	5.890664007	6.776345596	5.903101148	9.026344993	0.885681589	3.123243846
A_23_P117734	NM_152449	LYSMD4	8.047421512	8.924437235	7.288984904	8.570482054	0.877015723	1.28149715
A_33_P3436316	NM_001164603	ASXL1	9.009199659	9.884020162	9.266428917	10.51923469	0.874820503	1.252805776
A_24_P134392	NM_006948	HSPA13	9.201787995	10.06862366	8.801844584	10.13688222	0.866835666	1.33503764
A_33_P3378047	NM_178123	SESTD1	7.708276026	8.574400319	7.673333981	9.228049727	0.866124292	1.554715746
A_24_P304987	NM_013260	SAP30BP	10.0183281	10.86001622	9.175451478	10.68747028	0.841688119	1.512018806
A_24_P132787	NM_021252	RAB18	9.939422827	10.77047397	9.586900078	10.97200328	0.831051143	1.385103206
A_23_P157449	NM_005034	POLR2K	9.614230955	10.42436702	9.412665344	10.49133649	0.810136068	1.078671142
A_32_P199252	NM_001017963	HSP90AA1	11.15952955	11.94795829	10.61931538	11.70347297	0.788428733	1.08415759
A_23_P56894	NM_177538	CYP20A1	8.721077401	9.500877202	8.1174239	9.36880354	0.7797998	1.25137964
A_32_P90210	NM_001015892	TAF9	10.93535125	11.70678399	11.10357416	12.43258911	0.771432741	1.32901495
A_23_P104798	NM_001562	IL18	8.733217214	9.501667221	8.840793787	11.18258985	0.768450008	2.341796063
A_23_P100704	NM_139033	MAPK7	10.20448247	10.96825859	10.21744609	11.52574852	0.763776119	1.308302434
A_23_P203445	NM_001040697	UEVLD	7.668035267	8.416162421	7.372807669	8.585147115	0.748127153	1.212339446
A_33_P3251289	NM_018445	SELS	10.34514079	11.08383658	10.05110934	11.31585397	0.738695797	1.264744631
A_19_P00809368		XLOC_002749	5.667827877	6.403892747	6.299670244	8.661087309	0.736064871	2.361417065
A_33_P3325439	NM_001001992	USP16	9.485489894	10.21734776	8.442964354	9.791439171	0.731857864	1.348474818
A_32_P174083	NM_018947	CYCS	7.814350443	8.544110968	7.181901439	8.759809473	0.729760525	1.577908033
A_33_P3361393	NM_001412	EIF1AX	11.4312913	12.16074901	11.04422915	12.39808224	0.729457712	1.353853097
A_23_P99452	NM_000059	BRCA2	5.955046654	6.683204677	5.675542739	7.400304614	0.728158023	1.724761875
A_33_P3319905	NM_018643	TREM1	5.900013066	6.621094223	8.822046993	10.87617575	0.721081157	2.054128753
A_24_P272225	NR_027023	LOC645676	7.421081972	8.141093546	7.020909059	8.314534922	0.720011574	1.293625863
A_23_P58390	NM_152400	C4orf32	9.683231269	10.39150622	8.099466499	9.677597607	0.708274955	1.578131108
A_33_P3326634	NM_001164617	GPC3	5.663012162	6.36777786	6.42706427	9.659082028	0.704765698	3.232017758
A_23_P42096	NM_005922	MAP3K4	8.605163919	9.304658799	8.553276674	11.13202234	0.69949488	2.57874567
A_23_P133582	NM_004730	ETF1	10.16593233	10.86343852	10.2936346	11.41941932	0.697506192	1.125784717
A_23_P205646	NM_198794	MAP4K5	8.405216133	9.090135562	8.115661877	9.523976215	0.684919429	1.408314338
A_33_P3260317	NM_033334	NR6A1	5.515737551	6.198479679	5.419937102	6.470722624	0.682742128	1.050785522
A_23_P103201	NM_017761	PNRC2	11.14890784	11.82906554	11.03530103	12.34413046	0.680157699	1.30882943
A_33_P3289536	NM_001199835	SNX10	8.305319761	8.984762938	8.985461511	10.97476529	0.679443177	1.989303779
A_19_P00320780		XLOC_008144	7.532985466	8.211162235	7.337089183	8.733714497	0.678176769	1.396625314
A_23_P128246	NM_007076	FICD	8.616857997	9.294109541	7.813939414	9.678048454	0.677251544	1.864109041
A_23_P357811	NM_021038	MBNL1	13.40988708	14.08598238	12.55248178	14.2988388	0.676095299	1.746357013
A_33_P3238007	NM_178043	LARP1B	7.278425398	7.952719398	7.429381622	8.712946018	0.674294	1.283564396
A_23_P12874	NM_012341	GTPBP4	10.48096256	11.1540337	10.72909709	12.04217048	0.673071144	1.313073393
A_33_P3362353		ARAF	6.979415667	7.650044531	6.947382316	8.156687587	0.670628864	1.209305271
A_24_P288890	NM_181709	FAM101A	5.605284691	6.274199853	5.678052228	6.755418253	0.668915162	1.077366025
A_23_P313389	NM_003358	UGCG	8.430746084	9.098555479	8.08006411	9.760953213	0.667809396	1.680889104
A_33_P3368301	NM_212552	BOLA3	8.090595812	8.755868719	8.771130368	10.33106786	0.665272908	1.559937495
A_23_P41487	NM_015130	TBC1D9	9.883582272	10.54609312	9.537766865	10.62464687	0.662510845	1.086880005
A_23_P78248	NM_015515	KRT23	5.159590798	5.778798338	6.041395487	7.665645835	0.61920754	1.624250348
A_24_P68783	NM_012275	IL36RN	5.391750097	6.009281968	5.548967753	7.083663504	0.617531871	1.534695751
A_33_P3392784		CCDC147	5.408297141	6.025404224	5.649862005	6.851494713	0.617107084	1.201632708
A_23_P256473	NM_006379	SEMA3C	7.971566147	8.576466703	8.590667601	9.594307248	0.604900556	1.003639648
A_24_P43391	NM_018475	TMEM165	9.370784531	9.974292305	8.693104497	10.45808348	0.603507774	1.764978987
A_23_P417331	NM_004586	RPS6KA3	10.1236294	10.7069899	10.06141486	11.56499222	0.583360496	1.503577356
A_33_P3301040	NM_007072	HHLA2	6.525150921	7.099130322	6.031121992	7.98275263	0.573979401	1.951630637
A_23_P125639	NM_003410	ZFX	6.157925151	6.724923071	6.255131139	7.683493569	0.566997919	1.42836243
A_23_P207600	NM_002815	PSMD11	10.09584006	10.65624677	10.06620534	11.55910359	0.560406714	1.492898243
A_24_P76740	NM_032449	CC2D1B	9.546682666	10.08114167	9.018404286	10.1023485	0.534459	1.083944217
A_23_P312718	NM_015723	PNPLA8	10.7001956	11.2307534	10.68897508	12.01404478	0.530557803	1.325069697
A_23_P24716	NM_017870	TMEM132A	6.713108923	7.243666052	8.74344716	10.31130678	0.530557129	1.567859619
A_23_P137578	NM_015176	FBXO28	8.722723491	9.250032361	8.228784916	9.280849825	0.527308871	1.052064909
A_23_P74663	NM_005681	TAF1A	7.128674859	7.655408407	7.283070604	8.840888095	0.526733548	1.557817491
A_33_P3418125	NM_006851	GLIPR1	9.80673938	10.33247259	8.524724074	9.925712959	0.525733205	1.400988885
A_24_P261052	NM_015458	MTMR9	7.984616146	8.498450527	7.283849448	8.44703185	0.51383438	1.163182403
A_33_P3251932	NM_207002	BCL2L11	9.532884692	10.04524811	9.643161145	10.65090637	0.512363417	1.007745228
A_32_P54503	NR_024451	LOC100134229	7.780567563	8.290155455	7.04535504	8.830568124	0.509587892	1.785213084
A_33_P3335935	NM_144570	HN1L	7.031544786	7.531598739	6.285007228	7.626171902	0.500053953	1.341164674
A_23_P324754	NM_018689	KIAA1199	5.093514771	5.592776349	5.869168092	8.338839719	0.499261579	2.469671626
A_33_P3249674	XR_108749	LOC541472	5.480156589	5.955341685	5.520138981	6.614945496	0.475185096	1.094806515
A_23_P61127	NM_024122	APOO	10.95460334	11.42624497	12.69721445	14.17022425	0.471641625	1.473009797
A_23_P406702	NM_203459	CAMSAP2	8.098701557	8.554672801	7.407180465	8.786166663	0.455971243	1.378986197
A_23_P6891	NM_005875	EIF1B	11.01760322	11.46403594	11.49096217	13.45722323	0.446432718	1.966261063
A_23_P211738	NM_014517	UBP1	10.70701077	11.15284817	9.929231977	11.02484515	0.445837399	1.095613176
A_19_P00321687		XLOC_008174	6.159457855	6.576440789	5.981320954	6.988291618	0.416982935	1.006970664
A_24_P103886	NM_004508	IDI1	10.65401259	11.06948989	10.51804416	11.7268724	0.415477301	1.208828233
A_33_P3281905	NR_021493	LOC100144604	9.444642558	9.842514363	9.070280708	10.19023885	0.397871805	1.119958142
A_23_P18372	NM_032047	B3GNT5	10.69241077	11.08577305	9.563901883	11.34617271	0.393362273	1.78227083
A_23_P253932	NM_001033030	FAIM	9.231551604	9.619760794	10.43186521	12.0764416	0.388209189	1.644576387
A_33_P3278573	NM_001099680	MAGIX	7.328725396	7.710344461	7.470466289	8.858485037	0.381619064	1.388018748
A_23_P380815	NM_015634	KIAA1279	9.726678255	10.0900921	8.612875638	9.853974695	0.363413841	1.241099057
A_24_P26554	NM_020408	LYRM4	7.393492875	7.750292005	7.127272615	8.512532575	0.35679913	1.385259959
A_23_P205007	NM_002271	IPO5	8.056574451	8.406743873	7.792579172	9.154424073	0.350169422	1.361844901
A_19_P00801649		XLOC_013837	6.025351597	6.375383619	7.132407978	10.60414237	0.350032021	3.471734392
A_23_P341275	NM_015029	POP1	9.72275851	10.07104449	9.161883802	10.62785058	0.348285983	1.465966775
A_32_P203300	NM_001130679	EIF4E	8.429445795	8.774185149	8.168879897	9.457714687	0.344739354	1.28883479

A_24_P205213	NM_198709	ARSB	8.583573266	8.92040784	8.242685073	9.278952283	0.336834574	1.03626721
A_23_P19592	NM_015599	PGM3	9.428629951	9.762944997	9.624880517	10.84557408	0.334315046	1.22069356
A_23_P27810	NM_032689	ZNF607	9.373540771	9.684349366	9.423812253	10.52557531	0.310808595	1.10176306
A_33_P3242623	NM_014331	SLC7A11	11.19878923	11.50587223	8.25085602	12.13120329	0.307083005	3.880347266
A_23_P111981	NM_177457	LYNX1	9.106165416	9.412075825	9.267680158	10.50951009	0.305910409	1.241829928
A_23_P154849	NM_138983	OLIG1	5.355299576	5.64617699	6.634125283	8.200277338	0.290877414	1.566152055
A_23_P31866	NM_080651	MED30	8.81724755	9.033771481	7.598172276	8.851893532	0.216523931	1.253721257
A_23_P204324	NM_012062	DNM1L	8.859108437	9.062173327	8.46341161	9.716069105	0.20306489	1.252657496
A_33_P3337034	NM_003913	PRPF4B	8.248292457	8.451140025	8.397679981	9.425356684	0.202847568	1.027676704
A_23_P69141	NM_016141	DYNC1LI1	9.786080512	9.985445361	8.602457883	9.734349559	0.199364849	1.131891676
A_24_P149036	NM_001387	DPYSL3	6.738545866	6.932715135	9.157794267	12.55571369	0.194169269	3.397919426
A_23_P20683	NM_014878	KIAA0020	10.10093695	10.27152357	9.913226733	11.01445273	0.170586617	1.101225993
A_24_P47547	NM_006325	RAN	12.34987937	12.51999258	12.08669093	13.1410289	0.17011321	1.054337973
A_24_P385313	NM_002840	PTPRF	5.91372039	6.082956952	5.501235204	6.561524726	0.169236562	1.060289522
A_23_P500400	NM_080284	ABCA6	12.0201117	12.17210207	6.081077264	7.916930937	0.151990374	1.835853672
A_23_P18579	NM_006607	PTTG2	8.238230055	8.382994593	8.111136935	9.146669942	0.144764539	1.035533007
A_23_P96285	NM_022912	REEP1	7.04737844	7.188235669	6.83376782	8.325093079	0.140857229	1.491325259
A_24_P637982	NM_198446	C1orf122	12.75395917	12.86252377	12.82568181	14.51957679	0.108564605	1.693894983
A_24_P920646		CYP19A1	5.096753418	5.184141936	5.935829247	7.265842075	0.087388518	1.330012828
A_24_P163237	NM_020225	STOX2	6.710937087	6.778070835	7.432190675	9.137761839	0.067133748	1.705571164
A_33_P3386835		SLC20A2	7.597601019	7.657747227	6.767005707	7.805965878	0.060146209	1.03896017
A_33_P3274332	NM_016021	UBE2J1	9.423025531	9.462889075	10.39085196	11.48138388	0.039863544	1.090531917
A_33_P3409159	NM_015482	SLC22A23	5.91524566	5.952850669	5.57665545	7.710133292	0.03760501	2.133477842
A_23_P93464	NM_000056	BCKDHB	7.854881638	7.882730026	8.13738904	9.159881341	0.027848388	1.022492301
A_23_P59602	NM_019005	MIOS	10.48448978	10.50343638	10.32133147	11.40358448	0.018946599	1.082253007
A_23_P321511	NM_178450	37681	6.545163623	6.556767461	7.44922284	9.121243059	0.011603838	1.672020219
A_23_P48166	NM_002822	TWF1	10.83770079	10.83706681	9.753664917	10.91723493	-0.000633984	1.16357001
A_23_P201731	NM_004619	TRAF5	8.720853008	8.693732	7.884109915	9.541263358	-0.027121008	1.657153443
A_23_P134809	NM_003580	NSMAF	11.84498109	11.78958576	11.21596336	12.33663568	-0.055395329	1.120672323
A_32_P201979	NM_182765	HECTD2	7.407791495	7.342339773	6.073515053	7.898085276	-0.065451722	1.824570223
A_33_P3402545	NR_015394	LOC285205	5.394115345	5.327643065	5.500142195	7.107275744	-0.066472279	1.607133549
A_33_P3391476	NM_001130042	CRYZ	7.794067406	7.721232184	6.799507505	7.915953375	-0.072835222	1.11644587
A_23_P40847	NM_004267	CHST2	8.740654982	8.644460769	7.105326444	9.385154605	-0.096194213	2.279828161
A_23_P136964	NM_000328	RPGR	7.71683604	7.590584796	7.764303486	9.870592513	-0.126251243	2.106289026
A_33_P3358312	NM_016023	OTUD6B	8.552344225	8.406145877	7.709002189	8.716714296	-0.146198347	1.007712107
A_23_P339098	NM_017515	SLC35F2	6.726553299	6.570997035	6.595532328	7.95559907	-0.155556264	1.360066742
A_24_P54131	NM_022836	DCLRE1B	8.472179562	8.31533464	7.298046249	8.412996385	-0.156844922	1.114950136
A_23_P129466	NM_024997	ATF7IP2	8.55555678	8.384768468	7.100608451	8.506811286	-0.170788313	1.406202835
A_24_P269779	NM_004273	CHST3	5.744182527	5.569902705	5.87035612	6.930841405	-0.174279821	1.060485285
A_33_P3305840	NM_001195446	SRSF7	12.03185187	11.80692111	11.42345003	12.7478882	-0.224930753	1.32443817
A_24_P38276	NM_003505	FZD1	10.87028672	10.63700181	9.487797221	11.82261896	-0.233284913	2.334821742
A_33_P3245183	NM_001098213	HRH1	8.462618908	8.217307766	7.050670953	8.169706044	-0.245311142	1.119035091
A_23_P29684	NM_015873	VILL	6.877891029	6.596030423	7.022192073	8.337584031	-0.281860606	1.315391958
A_23_P43175	NM_144710	40422	10.24417147	9.936624383	8.381044518	9.515537899	-0.307547085	1.134493381
A_32_P162150	NM_152787	TAB3	8.856340734	8.52679202	8.457434093	9.674663811	-0.329548714	1.217229718
A_33_P3829391		LOC641510	6.523726601	6.182339288	5.642856494	8.260145094	-0.341387313	2.6172886
A_33_P3391656	NM_033550	TP53RK	8.519167699	8.130764966	7.963012257	9.28742142	-0.388402733	1.324409163
A_33_P3284557	NM_016653	ZAK	10.91561906	10.50165343	10.61021468	11.90305556	-0.413965627	1.29284088
A_24_P944827	NM_006395	ATG7	9.373458618	8.9558569	8.449900203	9.559576761	-0.417601718	1.109676559
A_33_P3271051	NM_004227	CYTH3	9.423268757	8.770845236	8.902204295	10.60604118	-0.652423521	1.703836881
A_23_P139704	NM_001946	DUSP6	11.04291327	10.29410131	11.40498485	13.00888506	-0.748811964	1.60390021
A_24_P130363	NM_181482	C18orf1	9.669403268	8.685432059	9.154722803	10.84287934	-0.983971209	1.688156537
A_23_P55107	NM_014683	ULK2	8.744987046	7.455939007	6.872870478	8.624117616	-1.289048038	1.751247138
A_33_P3296205	NM_183422	TSC22D1	9.623395455	7.908986989	11.61703627	12.7091836	-1.714408466	1.092147333
A_23_P17345	NM_005461	MAFB	13.43923202	11.20783014	11.40411844	12.48611173	-2.231401878	1.081993287
